# Structure and Role of a Ga-Promoter in Ni-Based Catalysts for the Selective Hydrogenation of CO_2_ to Methanol

**DOI:** 10.1021/jacsau.3c00677

**Published:** 2024-01-03

**Authors:** Nora K. Zimmerli, Lukas Rochlitz, Stefano Checchia, Christoph R. Müller, Christophe Copéret, Paula M. Abdala

**Affiliations:** † Department of Mechanical and Process Engineering, ETH Zürich, Leonhardstrasse 21, CH 8092 Zürich, Switzerland; ‡ Department of Chemistry and Applied Biosciences, ETH Zürich, Vladimir-Prelog-Weg 2, CH 8093 Zürich, Switzerland; § ESRF − The European Synchrotron, 71 Avenue des Martyrs, 38000 Grenoble, France

**Keywords:** bimetallic catalysts, CO_2_ hydrogenation, nickel, gallium, in situ, operando, X-ray absorption spectroscopy, X-ray total scattering, pair distribution function

## Abstract

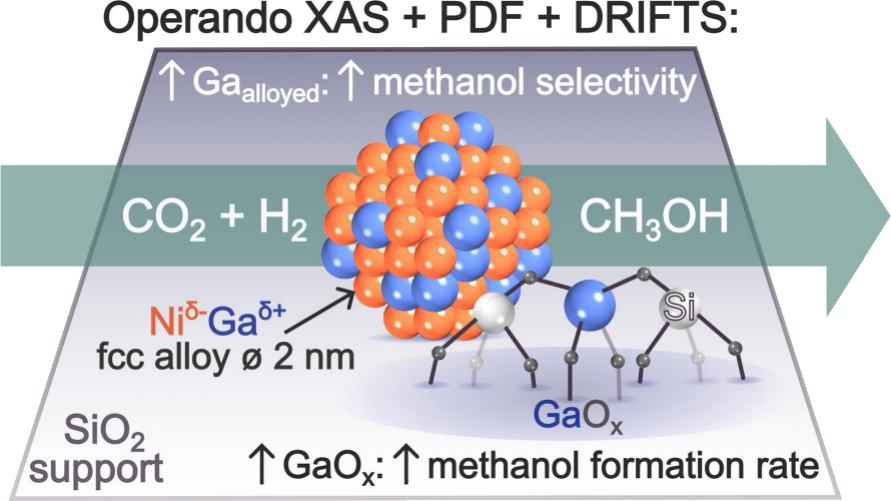

Supported, bimetallic catalysts have shown great promise for the selective hydrogenation of CO_2_ to methanol. In this study, we decipher the catalytically active structure of Ni–Ga-based catalysts. To this end, model Ni–Ga-based catalysts, with varying Ni:Ga ratios, were prepared by a surface organometallic chemistry approach. In situ differential pair distribution function (d-PDF) analysis revealed that catalyst activation in H_2_ leads to the formation of nanoparticles based on a Ni–Ga face-centered cubic (fcc) alloy along with a small quantity of GaO_*x*_. Structure refinements of the d-PDF data enabled us to determine the amount of both alloyed Ga and GaO_*x*_ species. In situ X-ray absorption spectroscopy experiments confirmed the presence of alloyed Ga and GaO_*x*_ and indicated that alloying with Ga affects the electronic structure of metallic Ni (viz., Ni^δ−^). Both the Ni:Ga ratio in the alloy and the quantity of GaO_*x*_ are found to minimize methanation and to determine the methanol formation rate and the resulting methanol selectivity. The highest formation rate and methanol selectivity are found for a Ni–Ga alloy having a Ni:Ga ratio of ∼75:25 along with a small quantity of oxidized Ga species (0.14  mol_Ni_^–1^). Furthermore, operando infrared spectroscopy experiments indicate that GaO_*x*_ species play a role in the stabilization of formate surface intermediates, which are subsequently further hydrogenated to methoxy species and ultimately to methanol. Notably, operando XAS shows that alloying between Ni and Ga is maintained under reaction conditions and is key to attaining a high methanol selectivity (by minimizing CO and CH_4_ formation), while oxidized Ga species enhance the methanol formation rate.

## 1 Introduction

The selective hydrogenation of CO_2_ to methanol (eq 1) enables us to convert a greenhouse gas, CO_2_, into a value-added product of high global demand, making this process a sustainable alternative to the commercial methanol synthesis based on syngas and reforming technology.^1^ Notably, the industrial catalyst used in the commercial process, viz., Cu/ZnO/Al_2_O_3_, suffers from deactivation and low methanol selectivity when used under CO_2_ hydrogenation conditions (eq 2).^1,2^ The main causes for catalyst deactivation include sintering of the active Cu^0^ nanoparticles^3^ and ZnO,^4^ oxidation of Cu,^4^ and poisoning of the active sites by hydroxyl groups at high concentrations of H_2_O or CO_2_.^5^
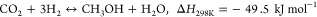
1
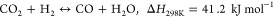
2

In that context, research efforts have been undertaken to improve Cu-based catalysts,^6^ as well as explore alternative metal-based catalysts such as Ni, Pd, and Au,^2,7,8^ focusing on understanding the nature of the active sites and the role of additional metals/metal oxides, which is critical to allow for a knowledge-driven catalyst design. For example, Ga-promoted transition metal catalysts, such as Cu–Ga,^9−12^ Ni–Ga,^13−17^ and Pd–Ga,^18−22^ have shown promising activity for the CO_2_ hydrogenation to methanol. In these catalysts, Ga was present in the form of an alloy that in many cases^9,12,14,18^ coexisted with oxidized Ga species under reaction conditions. The promotional effect of Ga in Ni is particularly noteworthy as Ni-based catalysts are typically very effective in the methanation reaction (eq 3).^23^ The introduction of Ga, forming Ni–Ga intermetallics (ordered alloys), has been shown however to improve the selectivity toward methanol, reaching up to ca. 60%.^14^
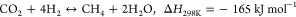
3This finding has triggered research into understanding the role of Ga in the CO_2_ hydrogenation pathway, the relationship between Ni–Ga phases and catalyst performance, and ultimately the structure of the active sites in this promising catalyst family.^13−15,24,25^ Previous works based on density functional theory (DFT) calculations have linked the δ-Ni_5_Ga_3_ phase to high methanol yields,^13^ while more recent studies on Ga-doped Ni(211) surfaces have argued that Ni is the active site and that alloying with Ga modifies the electronic structure of Ni through an electron transfer from Ga, promoting in turn the formation of oxygenates (methanol and CO) over methane.^15,26,27^

Despite these previous research efforts, the structure of the active sites in the Ni–Ga system has not been elucidated unequivocally. Two major challenges have limited progress in answering this central research question, viz., the lack of model catalyst systems with precise control over size, phase, and composition (i.e., Ni:Ga ratio) and the lack of detailed information concerning the catalyst structure (electronic and geometric) under operando conditions. With regard to the synthesis of Ni–Ga catalysts, previous works reported challenges in obtaining well-defined nanoparticles of a single phase as conventional approaches such as impregnation lead typically to catalysts containing multiple phases (e.g., both δ-Ni_5_Ga_3_ and α′-Ni_3_Ga),^14,16,25,28^ or mixtures of alloys and gallium oxide, which makes it impossible to identify the catalytically active motif.^14,25,29,30^ Furthermore, it has been reported that the oxidation of Ga to Ga_2_O_3_ species can promote methanol formation.^14^ Indeed, dealloying under CO_2_ hydrogenation conditions has been reported for related bimetallic M–M′ systems (Cu–Ga,^9,12^ Cu–Zn,^31^ and Pd–Ga^18^), generating a mixture of both metallic M (Cu/Pd) and (partially) oxidic M′ (Ga/Zn) species. Similarly, the ratio of M/M′ in bimetallic catalysts has been shown to have a pronounced influence on the catalyst activity, transitioning from promoting to poisoning effects.^10^ In the specific case of Ni–Ga, the δ-Ni_5_Ga_3_ phase has been proposed to be particularly active for CO_2_ hydrogenation to methanol, putting forward questions related to the optimal Ni:Ga ratio as well as the stability and the role of the alloy and/or the oxide interface on methanol selectivity and formation rate.

To shed light on these questions, we synthesized tailored Ni–Ga-based catalysts with varying Ni:Ga ratios and constant particle sizes (∼2 nm) using surface organometallic chemistry (SOMC)^32,33^ and thermolytic molecular precursor (TMP)^34^ approaches and interrogated (quantitatively) their structure under operando conditions using a combination of X-ray-based characterization techniques. In situ and operando differential pair distribution function (d-PDF) analysis of X-ray total scattering data and X-ray absorption spectroscopy (XAS) revealed that after activation in H_2_, the most selective catalyst contained nanoparticles with an fcc Ni–Ga alloy structure with a ratio Ni:Ga = 75:25 and a small quantity of oxidized Ga species, GaO_*x*_ (0.14  mol_Ni_^–1^). The presence of GaO_*x*_ appreciably increased the methanol formation rate while maintaining a high methanol selectivity when compared with a catalyst that contains an identical Ni–Ga alloy composition (Ni:Ga = 75:25) but a smaller GaO_*x*_ content (0.06  mol_Ni_^–1^). The structure of the catalysts formed after activation was maintained under reaction conditions (20 bar CO_2_:H_2_:N_2_ = 1:3:1, 230 °C), i.e., the quantities of Ga alloyed and GaO_*x*_ species remained constant. Furthermore, operando diffuse reflectance infrared Fourier transform spectroscopy (DRIFTS) provided additional insight into the surface species under reaction conditions whereby only the most active catalyst showed bands due to formate species. Combining these findings, we were able to conclude that the alloying of Ni with Ga is key to attaining a high methanol selectivity, while the presence of oxidized Ga species enhances appreciably the rate of methanol formation.

## 2 Results and Discussion

### 2.1 Catalyst Synthesis

**Figure 1:**
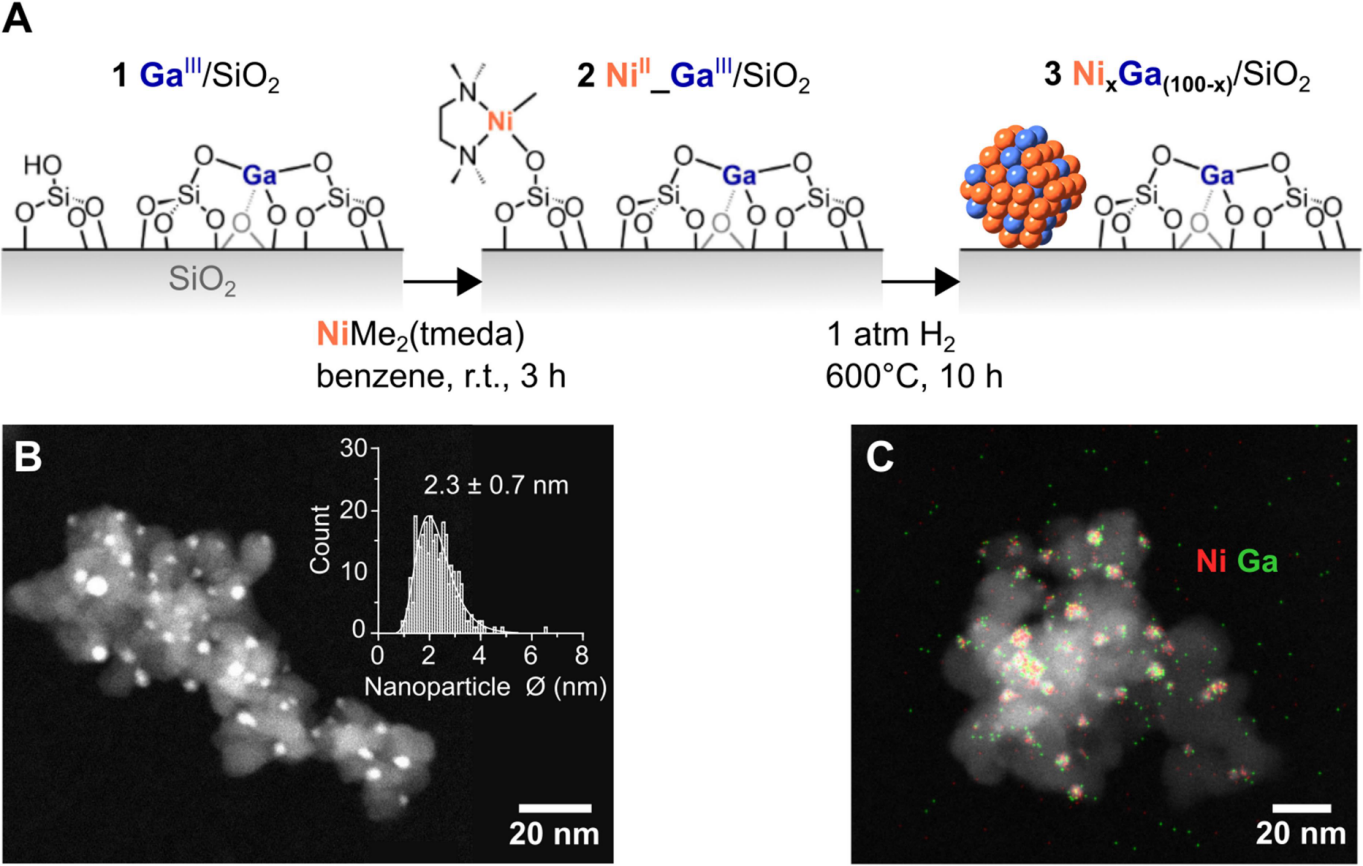
(A) Synthesis of bimetallic Ni_*x*_Ga_(100–*x*)_/SiO_2_ catalysts. (B) STEM image with the corresponding particle size distribution and (C) STEM-EDX image with overlaid Ni and Ga EDX signals of as-reduced Ni_65_Ga_35_/SiO_2_, selected here as a representative example for the TEM images of all Ni_*x*_Ga_(100–*x*)_/SiO_2_ presented in the Supporting Information.

We prepared a series of silica-supported Ni–Ga-based catalysts with varying Ni:Ga ratios via a SOMC-TMP approach (Figure 1A).^35^ Briefly, in all of the Ni-containing materials, Ni was introduced by grafting [Ni(CH_3_)_2_(tmeda)] onto the surface OH groups of Ga^III^/SiO_2_ (or SiO_2_, in case of the monometallic Ni material), whereby Ga^III^/SiO_2_ was produced by grafting [Ga(OSi(OtBu)_3_)_3_(THF)] onto SiO_2_ as reported previously.^36^ All materials were subsequently treated under H_2_ at 600 °C for 12 h, which yielded the as-prepared catalysts, denoted as Ni_*x*_Ga_(100–*x*)_/SiO_2_, where *x* is the nominal catalyst composition. The nominal Ni loading was kept constant at ca. 2 wt %, while the Ga loading was varied to obtain the desired nominal ratios of Ni:Ga (100:0, 75:25, 70:30, 65:35). The reference material Ga_100_/SiO_2_ (ca. 0.9 wt % Ga) was obtained by treating Ga^III^/SiO_2_ under H_2_ at 600 °C for 12 h. The final composition of the as-prepared catalysts was determined by elemental analysis (inductively coupled plasma optical emission spectroscopy, ICP-OES) and will be denoted as *x*_ICP_ (Table 1 and Supporting Information Table S1).

**Table 1:** Catalyst Composition, as Determined by ICP, Molar Ratio of Ni:Ga (−) in the fcc-Ni_*y*_Ga_(100–*y*)_ Alloy, and Molar Amounts of GaO_*x*_ and Ga_alloyed_ Normalized by the Catalysts’ Total Ni Content, as Determined by PDF and ICP Analyses

Catalyst	*x*_ICP_ in Ni_*x*_Ga_(100–*x*)_ (−)	Molar ratio of Ni:Ga in the fcc-Ni_*y*_Ga_(100–*y*)_ alloy (−)	Ga_alloyed_ (mol_Gaalloyed_ mol_Ni_^–1^)	GaO_*x*_ ( mol_Ni_^–1^)
Ni_65_Ga_35_/SiO_2_	67.5	74:26	0.34	0.14
Ni_70_Ga_30_/SiO_2_	71.9	75:25	0.33	0.06
Ni_75_Ga_25_/SiO_2_	76.4	82:18	0.23	0.08

Ex situ high-angle annular dark-field scanning transmission electron microscopy (HAADF-STEM) images of the as-prepared materials recorded under air-free conditions showed well-dispersed nanoparticles on the SiO_2_ support with an average diameter of around 2 nm (Figure 1B and Supporting Information Figures S1, S5, S8, and S12). These STEM images further confirmed that the SOMC-based synthesis approach yielded catalysts of very similar particle size, independent of the Ni:Ga ratio. Hence, any change in product formation rates could not be attributed to changes in the surface area of the nanoparticles. Furthermore, STEM-EDX images of Ni_100_/SiO_2_, Ni_75_Ga_25_/SiO_2_, Ni_70_Ga_30_/SiO_2_, and Ni_65_Ga_35_/SiO_2_ showed a spatial overlap between the Ni and Ga EDX signals in all of the as-prepared materials (Figure 1C and Supporting Information Figures S7, S10, and S14). Turning to the reference Ga_100_/SiO_2_, Ga was found to be highly dispersed with no visible nanoparticle formation (Supporting Information Figures S3 and S4), in line with previous studies.^35^

### 2.2 Structure of the Catalysts after In Situ Activation

**Figure 2:**
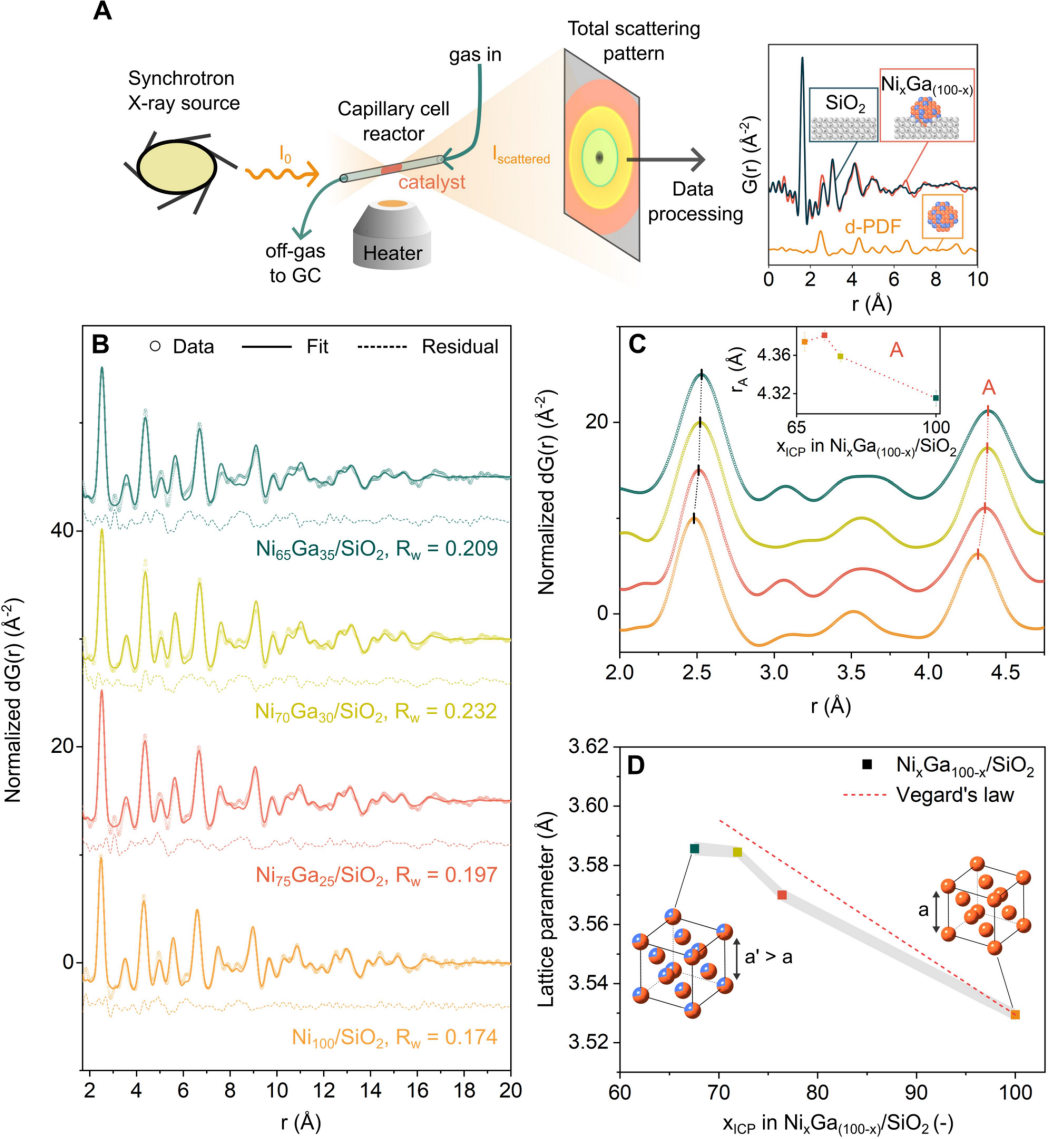
(A) Schematic representation of a X-ray total scattering experiment and the resulting d-PDF data. (B) d-PDF data fitted to a fcc-Ni_*y*_Ga_(100–*y*)_ alloy. (C) Zoom into the region *r* = 2–4.75 Å of the d-PDF data. The inset plots the fitted position of the metal–metal pair correlation labeled A as a function of *x*_ICP_ in the catalysts. (D) Cubic lattice parameter as a function of the fraction of Ni, as determined by elemental analysis. The error bars are represented by the area shaded in gray. Conditions: 1 bar H_2_, 230 °C, after in situ activation.

Prior to CO_2_ hydrogenation experiments, the catalysts were activated in 1 bar H_2_ at 600 °C for 1 h, and this process was monitored by in situ d-PDF and XAS. Here, we focus on the analysis of the catalyst structure obtained after their in situ activation. The evolution of the air-exposed catalysts during activation is described in the Supporting Information. d-PDF was obtained by subtracting the PDF signal of the support (i.e., SiO_2_) from the PDF data of the entire catalyst (Figures 2A and S22).^37,38^ For the d-PDF analysis of in situ activated Ni_100_/SiO_2_, Ni_75_Ga_25_/SiO_2_, Ni_70_Ga_30_/SiO_2_, and Ni_65_Ga_35_/SiO_2_, synchrotron X-ray total scattering data were collected at the reaction temperature of 230 °C in 1 bar H_2_. The SiO_2_-subtracted X-ray total scattering and d-PDF data of the in situ activated catalysts are shown in Figures S21 and 2B (data collected at 230 °C). The reciprocal space data of the activated catalysts showed broad, yet clear Bragg peaks for all of the catalysts that can be indexed, independent of the Ni:Ga ratio, as a face-centered cubic (fcc)-type structure (Supporting Information Figure S21). Modeling of the d-PDF confirmed that all the nanoparticles have an fcc type structure and revealed an average diameter of the nanoparticles of ca. 2 nm that was invariant to the catalysts’ elemental composition, in line with STEM-EDX measurements (Supporting Information Figure S27). In addition, fitting of the d-PDF data revealed an increase in the cubic lattice parameter of the fcc-Ni_*y*_Ga_(100–*y*)_ alloy [where *y*:(100–*y*) is the Ni:Ga ratio in the alloy] with increasing Ga content, which was attributed to the incorporation of Ga into the (nano)alloy structure, causing a tensile strain in the fcc lattice (Figure 2D, Supporting Information Table S3). In line with the formation of Ni–Ga alloys, d-PDF modeling also revealed an increase in the atomic displacement factors (ADFs) with respect to Ni_100_/SiO_2_ due to a broader distribution of interatomic distances in the alloys (Supporting Information Figure S28).^39^ The agreement factors (*R*_w_) in all of the fittings were in the range of 0.17–0.23 which are typical values for the PDF of small nanoparticles.^40,41^ It is possible that defects inside and/or on the surface of the nanoparticles led to some misfit between the experimental data and the calculated PDF (e.g., slight misfit in the intensity of the peaks between 5 and 7 Å, Figure 2B), which are often present in nanoparticles;^42,43^ however, the peak positions of the PDF are well explained by the models. The lattice parameters extracted from the fitting of the d-PDF data were independent of the *r*-range fitted (1.7–25 Å) as shown in Figures 2D and S31. This indicates that the local and midrange structures of the alloy are comparable. Concerning the detection of oxidized species by d-PDF, it has to be stated that the detection of Ga–O pairs by d-PDF is challenging (expected at ca. 1.7–2.0 Å)^44^ due to the presence of termination (noise) ripples arising from the finite *Q*-range,^45^ and the low scattering cross-section of oxygen atoms relative to that of the higher *Z* elements Ni and Ga.^46^ Notably, the fcc Ni–Ga alloys (bulk) follow Vegard’s law, i.e., there is a linear relationship between the Ni:Ga ratio and the cell parameter (Supporting Information Table S4).^47^ However, when plotting the cell parameters extracted from the supported nanoparticles against the catalysts’ composition determined by ICP (Figure 2D) together with the predicted cell parameters using Vegard’s law (dashed curve), we observe that the determined cell parameters were lower than the values expected from Vegard’s law, indicating that not all of the Ga in the catalyst was incorporated into the fcc structure of the (alloy) nanoparticles and hence remained as oxidized Ga species (GaO_*x*_), as confirmed by XAS (vide infra). Thus, we next quantified the amount of alloyed Ga species (Ga_alloyed_) via Vegard’s law, and the amount of GaO_*x*_ as the difference between the catalyst’s total Ga content measured by ICP and Ga_alloyed_ (see Table 1 and the Supporting Information Section 3.1 for details). A key observation from this analysis was that the lattice parameters of the alloy phases in Ni_70_Ga_30_/SiO_2_ and Ni_65_Ga_35_/SiO_2_ were equal within the error of the fitting, corresponding to an fcc alloy of composition Ni:Ga = 75:25 in both materials, despite the fact that Ni_65_Ga_35_/SiO_2_ had a higher total Ga content than Ni_70_Ga_30_/SiO_2_ (Tables 1 and S1 in the Supporting Information). Thus, Ni_65_Ga_35_/SiO_2_ contained a larger quantity of GaO_*x*_ (0.14  mol_Ni_^–1^) than Ni_70_Ga_30_/SiO_2_ (0.06  mol_Ni_^–1^). Furthermore, it is worth noting that the composition of the alloy nanoparticles in Ni_70_Ga_30_/SiO_2_ and Ni_65_Ga_35_/SiO_2_ was the same as the composition of the (intermetallic) α′-Ni_3_Ga phase. From our data, we could not exclude an ordering of the metals in the catalysts as in the α′-Ni_3_Ga intermetallic phase due to the very similar structure of fcc alloys and α′-Ni_3_Ga. The random fcc alloy (with a composition Ni:Ga = 75:25) differs from the ordered α′-Ni_3_Ga only in the occupancy of Ni and Ga in the fcc sites: in the random alloy, Ga and Ni atoms randomly occupy the same sites, whereas in α′-Ni_3_Ga, Ga sits only at the corners and Ni in the center of the faces of the cubic unit cell (see Supporting Information Figure S30). Due to the reduced symmetry of the α′-Ni_3_Ga structure (being a superstructure of the fcc structure) compared to that of the fcc random alloy, additional low-intensity reflections would be expected in its diffraction pattern.^48^ However, since Ni and Ga have very similar scattering properties, it is challenging to detect such weak reflections. Similarly, only tiny differences would be expected in the magnitude of the PDF peaks. However, previous reports have shown that intermetallic phases are only formed above a certain critical nanoparticle size,^49−51^ and thus, we hypothesize that it is unlikely that ordering took place in ∼2 nm-sized (nano)alloys.

To probe the electronic and local structure of Ga and Ni, we performed XAS experiments at the Ni and Ga K-edges. XANES analysis provided insight into the metal oxidation states, while EXAFS yielded quantitative information concerning the local structure of Ni and Ga in the materials. In this context, it is worth noting that Ga K-edge XAS is more sensitive in probing for the presence of GaO_*x*_ species than d-PDF. In the in situ XAS experiments, the same capillary reactor cell was used as in the d-PDF experiments, allowing to directly confront the respective results. Due to limited synchrotron beamtime, the in situ XAS experiments were performed on the catalyst with the highest Ga content (Ni_65_Ga_35_/SiO_2_), one with lower Ga content (Ni_75_Ga_25_/SiO_2_) and the references Ni_100_/SiO_2_ and Ga_100_/SiO_2_ (collected ex situ in airtight capillaries).

**Figure 3:**
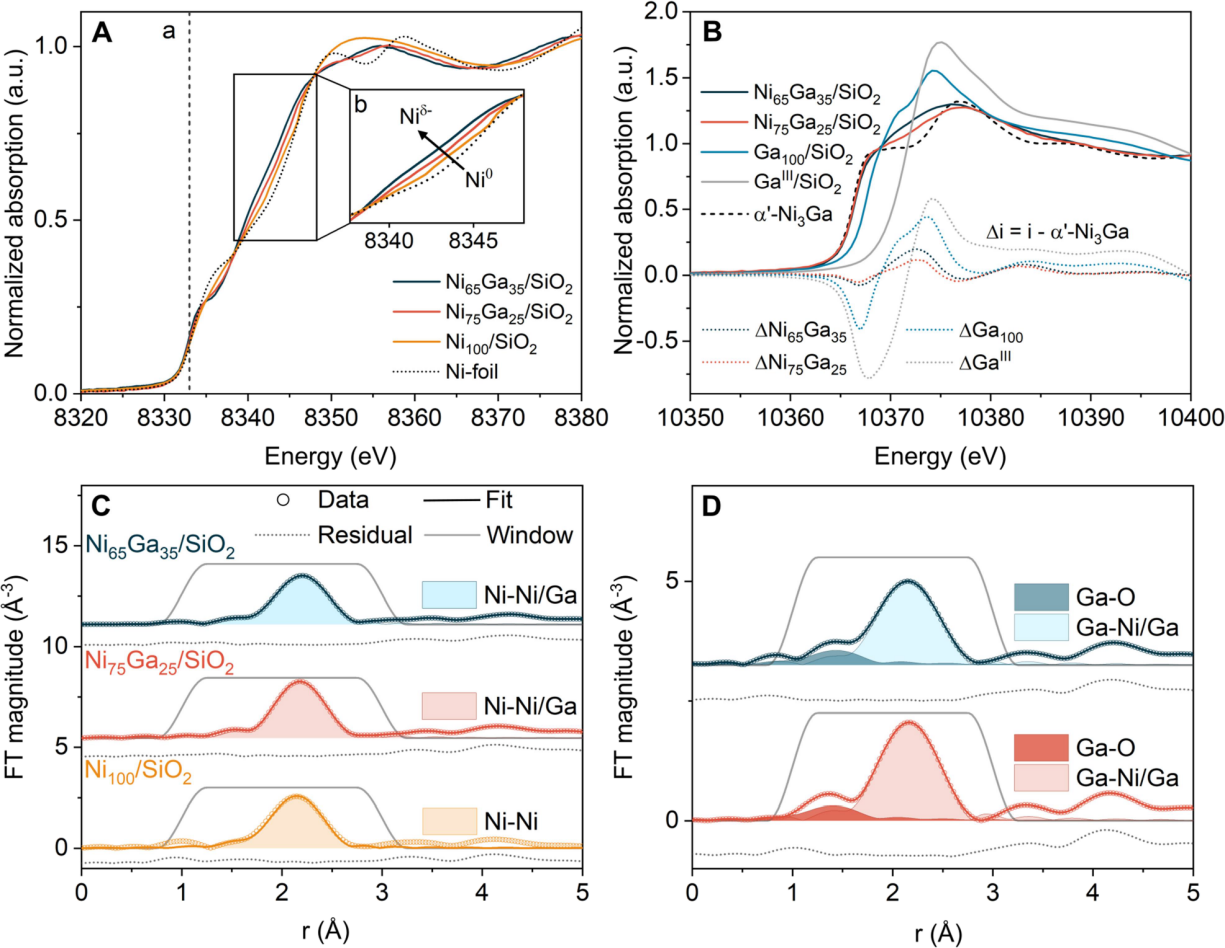
XAS data of the activated catalysts and reference materials: (A) normalized Ni K-edge XANES of Ni_65_Ga_35_/SiO_2_, Ni_75_Ga_25_/SiO_2_, and Ni_100_/SiO_2_ and a Ni-foil reference. Line “a” denotes the position of the maximum of the first derivative of the Ni K-edge XANES of Ni foil (8333 eV). Inset “b” shows a shift of the Ni absorption edge to lower energies with increasing Ga content, indicating the formation of Ni^δ−^ species upon alloying of Ni with Ga. (B) Normalized Ga K-edge XANES of Ni_65_Ga_35_/SiO_2_, Ni_75_Ga_25_/SiO_2_, and Ga_100_/SiO_2_, as well as the α′-Ni_3_Ga and Ga^III^/SiO_2_ references. The dotted lines represent difference XANES (Δ*i* = *i* – α′-Ni_3_Ga). Experimental and fitted Fourier transforms of the (C) Ni K-edge and (D) Ga K-edge EXAFS data (plots not corrected for the phase shift). The contributions of the individual Ni–M, Ga–M (M = Ni,Ga), and Ga–O coordination spheres to the fits are shown as shaded areas. Conditions: 1 bar H_2_, 50 °C, after in situ activation.

The in situ acquired Ni K-edge XANES spectra of the catalysts (after activation and collected at 50 °C) are presented in Figure 3A. The edge position [determined as the maximum of the first derivative of the normalized absorption μ(*E*)] was at ca. 8333 eV in all of the catalysts, indicating that Ni is in its metallic state, and no oxidized Ni species were present in the activated catalysts. Taking a closer look into the second edge feature labeled as “b” revealed a shift to lower energies for Ni_65_Ga_35_/SiO_2_ (by ca. −1.2 eV at μ_norm_ = 0.6 au) and Ni_75_Ga_25_/SiO_2_ (by ca. −0.5 eV at μ_norm_ = 0.6 au) with respect to Ni_100_/SiO_2_. Moreover, the shape of the XANES features of Ni_75_Ga_25_/SiO_2_ and Ni_65_Ga_25_/SiO_2_ in the white line region was different from that of Ni_100_/SiO_2_ and the Ni foil. According to previous studies, the observed XANES features in Ni_75_Ga_25_/SiO_2_ and Ni_65_Ga_25_/SiO_2_ were interpreted as an electron transfer from Ga to Ni in the Ni–Ga alloy (Ga^δ+^/Ni^δ−^), whereby the electron transfer was most pronounced in Ni_65_Ga_35_/SiO_2_ due to the higher content of Ga in the alloy, in line with d-PDF analysis (vide supra).^52,53^

Turning to Ga K-edge XANES, the respective data of the activated catalysts are plotted in Figure 3B together with α′-Ni_3_Ga as a reference for a Ni–Ga alloy and Ga^III/^SiO_2_ as a reference for a material containing only Ga^3+^ (where Ga^3+^ is in tetrahedral coordination with oxygen as reported previously).^36^ In addition, we collected Ga K-edge XANES data of activated Ga_100_/SiO_2_, which also contains Ga^3+^ indicated by the white line feature at ca. 10375.0 eV. However, activated Ga_100_/SiO_2_ also exhibits a feature at ca. 10371.5 eV, which can be assigned to Ga^+^ and/or Ga hydride species ([GaH_2_]^+^ or [GaH]^2+^), that were formed during activation in H_2_.^54,55^ Here, we refer to this mixture of Ga species as GaO_*x*_ (representing a mixture of Ga^3+^/ Ga^+^/ Ga hydride species).^54,55^ The energy positions of the Ga K-edge in Ni_75_Ga_25_/SiO_2_ and Ni_65_Ga_35_/SiO_2_ were within 0.1 eV of that of the bulk α′-Ni_3_Ga reference, indicating that most Ga species were in an alloyed state. Moreover, the Ga K-edge XANES spectra of both catalysts exhibited two main features at 10368.0 and 10377.0 eV, which were due to Ga alloyed with Ni. In fact, these features were present in α′-Ni_3_Ga and have been assigned to transitions to unoccupied Ga 4p states which are hybridized with Ni 3d/non-d bands of α′-Ni_3_Ga above the Fermi level.^53^ However, the white line feature of Ni_75_Ga_25_/SiO_2_ and Ni_65_Ga_35_/SiO_2_ (in the range 10370–10375 eV) had a higher intensity compared to that of α′-Ni_3_Ga, suggesting the presence of a minor quantity of oxidized Ga species in the activated catalysts. Indeed, by evaluating the difference spectra (differences with respect to α′-Ni_3_Ga, Figure 3B), we concluded that Ni_*x*_Ga_(100–*x*)_/SiO_2_ contained predominantly Ga_alloyed_ with some minor quantity of GaO_*x*_ species.^54,56,57^ Next, we performed linear combination fittings (LCFs) of the Ga K-edge XANES data of Ni_75_Ga_25_/SiO_2_ and Ni_65_Ga_35_/SiO_2_ using α′-Ni_3_Ga and Ga_100_/SiO_2_ as references for the Ga_alloyed_ and GaO_*x*_ species, respectively. LCF analysis yielded a ratio GaO_*x*_:Ga_alloyed_ = 31:69 for Ni_65_Ga_35_/SiO_2_ and GaO_*x*_:Ga_alloyed_ = 19:81 for Ni_75_Ga_25_/SiO_2_ (corresponding to 0.15 and 0.05  mol_Ni_^–1^ in Ni_65_Ga_35_/SiO_2_ and Ni_75_Ga_25_/SiO_2_, respectively; Supporting Information Table S6 and Figures S33 and S34) in line with the values obtained in Table 1.

We note that linear combination using the α′-Ni_3_Ga and Ga_100_/SiO_2_ references resulted in the best agreement between the experimental data and the fit when compared to using the combination α′-Ni_3_Ga and Ga^III^/SiO_2_ or α′-Ni_3_Ga and β-Ga_2_O_3_, indicating the presence of GaO_*x*_ in Ni_*x*_Ga_(100–*x*)_/SiO_2_. Supplementary LCF results including an extended selection of Ga K-edge XANES references (Ga-foil, β-Ga_2_O_3_) can be found in the Supporting Information (Table S6, Figures S33 and S34). However, as the quantitative analysis of Ga K-edge XANES data containing multiple (alloyed and oxidized) species can be subject to errors, as the Ga features are affected in a convoluted fashion by the oxidation state, electronic interactions (e.g., from alloying), as well as the coordination environment in the oxide and the size and phases of the alloyed nanoparticles, additional EXAFS analysis was performed to probe in more detail the presence of Ga–O atomic pairs.

The *k*^2^-weighted EXAFS oscillations (*k*^2^Χ(*k*)) and the corresponding magnitude of the Fourier transform (FT) at the Ni K-edge of Ni_100_/SiO_2_, Ni_75_Ga_25_/SiO_2_, and Ni_65_Ga_35_/SiO_2_ are presented in Figures S38 and S40 in the Supporting Information and Figure 3C. We observed a first neighboring Ni–M shell (M = Ni, Ga) at ca. 2.2 Å and no evidence of a Ni–O shell, in line with XANES analysis. The EXAFS data at the Ga K-edge of Ni_75_Ga_25_/SiO_2_ and Ni_65_Ga_35_/SiO_2_ are presented in Figures 3D, S38 and S40 in the Supporting Information and evidenced a prominent Ga–M (M = Ni or Ga) shell and a weak Ga–O shell, indicative of a minor quantity of oxidized Ga species in line with XANES. Fitting the Ga and Ni K-edge EXAFS data allowed us to determine the average interatomic distances, coordination numbers (CN), and σ^2^ (the mean square variation in path length also referred to as Debye Waller factors) of the first Ni–M, Ga–M, and Ga–O shells (Supporting Information Table S8). This analysis revealed an increase in the average Ni–M distance with increasing Ga content, i.e., 2.48 < 2.50 < 2.53 Å for Ni_100_/SiO_2_, Ni_75_Ga_25_/SiO_2_, and Ni_65_Ga_35_/SiO_2_, respectively (Supporting Information Figure S41), in line with d-PDF analysis that showed an increase in the lattice parameter as Ga is incorporated into the Ni–Ga alloy fcc structure. The Ga–M distances were determined to be 2.52 Å in both Ni_75_Ga_25_/SiO_2_ and Ni_65_Ga_35_/SiO_2_, which were close to the Ni–M distances determined by EXAFS fitting of the Ni K-edge data. The σ^2^ values obtained for Ga–M were slightly higher than those for Ni–M, suggesting a somehow higher degree of disorder around Ga. The average CN for Ni_100_/SiO_2_ was 9(1) (number in parentheses represents the standard deviations obtained from the fittings), whereas in Ni_75_Ga_25_/SiO_2_, the CN(Ga–M) was close to CN(Ni–M) [i.e., CN(Ni–M) = 9.0(6), CN(Ga–M) = 9(2)] in line with an homogeneous distribution of Ga within the nanoalloy (i.e., no Ga or Ni surface segregation). In Ni_65_Ga_35_/SiO_2_, the CN(Ni–M) = 8.8(6) was slightly larger than CN(Ga–M) = 8.1(8), which we attribute to a larger quantity of oxidized Ga species (GaO_*x*_) in this catalyst, leading to a decrease in CN(Ga–M). Indeed, to fit the Ga K-edge EXAFS data of Ni_75_Ga_25_/SiO_2_ and Ni_65_Ga_35_/SiO_2_, a Ga–O path was required. The Ga–O distances were at ca. 1.84–1.87 Å with a CN of 0.6(3) and 0.7(1) for Ni_75_Ga_25_/SiO_2_ and Ni_65_Ga_35_/SiO_2_, respectively, providing further evidence for the presence of GaO_*x*_ species in both catalysts. However, it was not possible to quantify the amount of GaO_*x*_ species via EXAFS.

To summarize, the combined d-PDF and XAS analyses show that Ni_65_Ga_35_/SiO_2_, Ni_70_Ga_30_/SiO_2_, and Ni_75_Ga_25_/SiO_2_ contained alloyed nanoparticles of ∼2 nm in size with an fcc structure along with small quantities of GaO_*x*_ species. Using the extracted cell parameters and the ICP-determined Ni and Ga contents in the catalysts, the alloy composition and the quantity of GaO_*x*_ were determined. Ni_65_Ga_35_/SiO_2_ and Ni_70_Ga_30_/SiO_2_ contained alloy nanoparticles with a Ni:Ga ratio of ca. 75:25, but Ni_65_Ga_35_/SiO_2_ contained more GaO_*x*_ species (0.14  mol_Ni_^–1^) compared to Ni_70_Ga_30_/SiO_2_ (0.06  mol_Ni_^–1^). On the other hand, Ni_75_Ga_25_/SiO_2_ contained an alloy with a higher Ni:Ga ratio of 82:18 compared to Ni_65_Ga_35_/SiO_2_ and Ni_70_Ga_30_/SiO_2_ along with a GaO_*x*_ content of 0.08  mol_Ni_^–1^, i.e., in between that of Ni_65_Ga_35_/SiO_2_ and Ni_70_Ga_30_/SiO_2_. XAS supported the findings of the d-PDF analysis in that Ni and Ga formed Ni–Ga alloys and that there were minor quantities of oxidized Ga species (ca. 0.05 and 0.15  mol_Ni_^–1^ in Ni_75_Ga_25_/SiO_2_ and Ni_65_Ga_35_/SiO_2_, respectively). Ni K-edge XANES data pointed to a different electronic structure (electron transfer from Ga to Ni) in the Ni–Ga catalysts compared to the monometallic Ni reference (Ni_100_/SiO_2_) due to the alloying of Ni with Ga.

### 2.3 Catalytic Performance and Role of Alloyed Ga and GaO_*x*_

**Figure 4:**
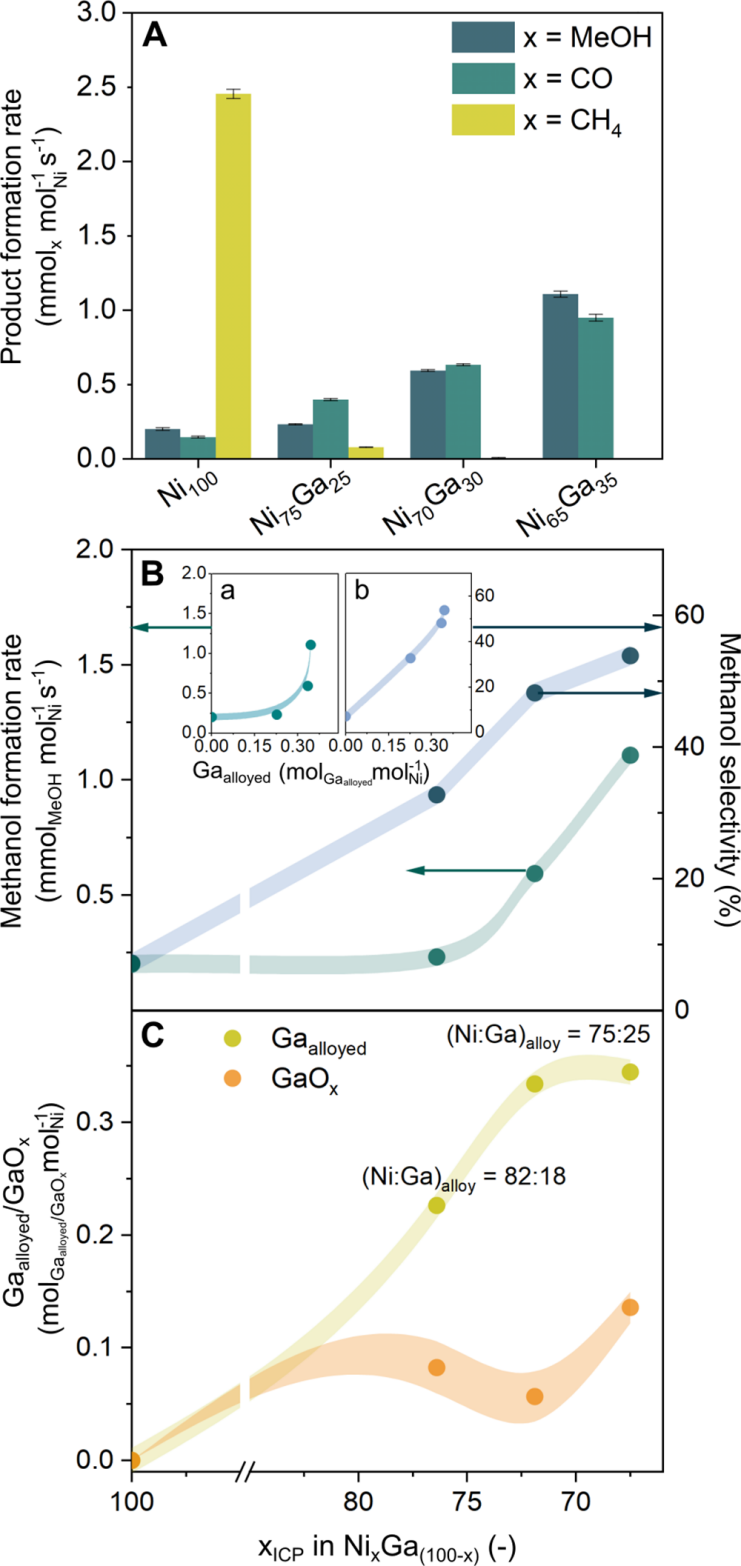
(A) Product formation rates over Ni_*x*_Ga_(100–*x*)/_SiO_2_. (B) Methanol formation rate and methanol selectivity (averaged over the first 180 min of TOS) as a function of *x*_ICP_ in Ni_*x*_Ga_(100–*x*)_/SiO_2_, as determined by ICP. The insets plot the methanol formation rates and methanol selectivity as a function of the Ga_alloyed_ content normalized by the catalysts’ Ni content. (C) Ga_alloyed_ and GaO_*x*_ contents as a function of *x*_ICP_. The labels denote the molar ratio Ni:Ga in the alloy, as determined from PDF analysis. Shaded lines are guides to the eye.

We determined the catalytic performance of the different materials under the following CO_2_ hydrogenation conditions: fixed bed reactor, 25 bar, CO_2_:N_2_:H_2_ = 1:1:3, 230 °C, and a gas hourly space velocity of 60 L gcat^–1^ h^–1^. All product (methanol, CO, and CH_4_) formation rates are normalized by the Ni content (Figure 4A) as Ni has been proposed as the active site whereby its electronic structure is modified by neighboring Ga atoms.^15,26^ Due to the small and invariant size of the Ni–Ga nanoparticles in the catalysts (ca. 2 nm according to d-PDF analysis), corresponding to a surface:volume ratio of 3:1 nm^–1^, we can infer that normalizing the product formation rates by the total Ni content (ca. 2 wt % in all catalysts) offers a meaningful basis for examining the impact of Ga addition on the catalytic activity of Ni-based nanoparticles. The catalytic performance per g of catalyst is reported in Table S1 in the Supporting Information. Interestingly, the rate of CO_2_ conversion decreases first by ca. 75% when transitioning from Ni_100_/SiO_2_ (2.8  mol_Ni_^–1^ s^–1^) to Ni_75_Ga_25_/SiO_2_ (0.7  mol_Ni_^–1^ s^–1^) and slightly increases again in catalysts with higher nominal Ga contents in the following order: Ni_75_Ga_25_/SiO_2_ < Ni_70_Ga_30_/SiO_2_ < Ni_65_Ga_35_/SiO_2_ (Figure 4A). Notably, this change of CO_2_ conversion rates was accompanied by a change in product selectivity, viz., from mostly methane when using pure Ni to methanol for Ni–Ga. The methanol formation rates of Ni_100_/SiO_2_ and Ni_75_Ga_25_/SiO_2_ were very similar, i.e., ca. 0.2 mmol_MeOH_ mol_Ni_^–1^ s^–1^ but were significantly higher for Ni_70_Ga_30_/SiO_2_ (0.6 mmol_MeOH_ mol_Ni_^–1^ s^–1^) and Ni_65_Ga_35_/SiO_2_ (1.1 mmol_MeOH_ mol_Ni_^–1^ s^–1^), while Ga_100_/SiO_2_ did not convert any CO_2_ to any significant extent (Table S1). We would like to note here that methane and CO, but not methanol, have been typically reported products from CO_2_ hydrogenation over monometallic Ni/SiO_2_ catalysts. We hypothesize that methanol formation over Ni_100_/SiO_2_ could be due to the presence of very small Ni clusters which interact strongly with the SiO_2_ support and show a different selectivity profile compared to larger Ni nanoparticles.^58^ Generally, a remarkable shift in methanol selectivity was observed upon the addition of Ga for the Ni_*x*_Ga_(100–*x*)_/SiO_2_ series (Figure 4, Supporting Information Figure S17). Specifically, the methanol selectivity increased in the following order: Ni_100_/SiO_2_ (7%) < Ni_75_Ga_25_/SiO_2_ (33%) < Ni_70_Ga_30_/SiO_2_ (48%) < Ni_65_Ga_35_/SiO_2_ (54%). In parallel, while Ni_100_/SiO_2_ displayed a high methane selectivity (88%), the methane selectivity decreased to 11% in Ni_75_Ga_25_/SiO_2_, and no methane was observed for Ni_70_Ga_30_/SiO_2_ and Ni_65_Ga_35_/SiO_2_.

To further analyze the role of the composition of the alloy and the quantity of GaO_*x*_ in the catalytic performance of the series of catalysts studied, the rate of methanol formation, the methanol selectivity, S_MeOH_, and the quantities of Ga_alloyed_ and GaO_*x*_ normalized by the catalysts’ Ni contents are plotted as a function of the Ni:Ga ratio determined by ICP (*x*_ICP_ in Ni_*x*_Ga_(100–*x*)_/SiO_2_, Figure 4B,C). Contrasting the individual trends, we observe that S_MeOH_ increased quasi-linearly (in the range considered here) with the amount of Ga_alloyed_ (Figure 4B, inset b). On the other hand, the methanol formation rate related nonlinearly with Ga_alloyed_ and increased significantly once the composition of the alloy nanoparticle had reached the composition Ni:Ga = 75:25 (Figure 4B, inset a). Moreover, it was observed that for catalysts with an optimal alloy composition of Ni:Ga = 75:25 (ratio determined after activation according to d-PDF), an increase in the quantity of GaO_*x*_ led to a significant increase in the rate of methanol formation (at a stable methanol selectivity).

### 2.4 Catalysts’ Structure during CO_2_ Hydrogenation to Methanol Conditions

**Figure 5:**
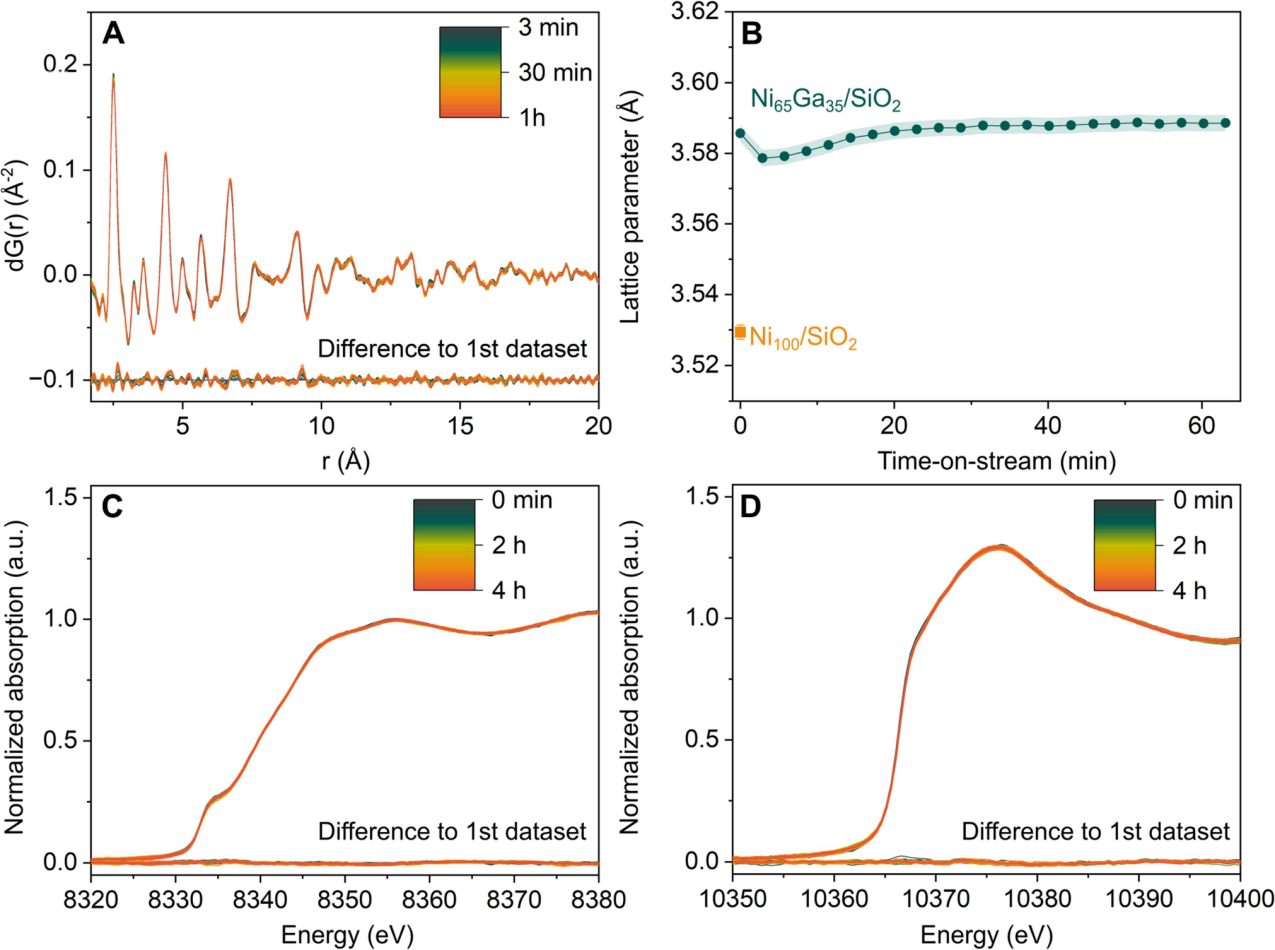
Operando characterization of Ni_65_Ga_35_/SiO_2_. (A) d-PDF data collected over 60 min of TOS. The difference between each scan collected at TOS with respect to the first data collected after 3 min under reaction conditions. (B) Lattice parameter obtained via the fitting of the d-PDF shown in (A) as a function of TOS. The first value of the cell parameter (TOS = 0) corresponds to the value determined after catalyst activation (data collected in H_2_, Figure 2D). The lattice parameter obtained for activated Ni_100_/SiO_2_ is plotted for comparison. Normalized XANES collected over 4 h TOS at the (C) Ni K-edge and (D) Ga K-edge. Conditions: 20 bar, CO_2_:H_2_:N_2_ = 1:3:1, 230 °C.

To track the catalyst structure and possible changes under reaction conditions, we further collected operando d-PDF and XAS data on the most active catalyst, i.e., Ni_65_Ga_35_/SiO_2_, under CO_2_ hydrogenation conditions (data collection started at the time of the gas switch from 20 bar N_2_ to 20 bar CO_2_:H_2_:N_2_ = 1:3:1 at 230 °C after the activation in hydrogen). We monitored the catalyst’s structure over ca. 3.5 h (total scattering) and 4 h (XAS) time-on-stream (TOS) (Figure 5). During the operando experiments, the outlet gas stream was analyzed online by gas-chromatography. In addition, similar operando d-PDF experiments were performed for Ni_70_Ga_30_/SiO_2_ and Ni_75_Ga_25_/SiO_2_ for 1 h TOS.

d-PDF analysis revealed that the nanocrystalline fcc phase in Ni_65_Ga_35_/SiO_2_ remained stable under reaction conditions (Figure 5A, Supporting Information Figure S25); also, no growth in particle size was observed. We detected a minor increase by ca. 0.3% in the lattice parameter (from 3.579 to 3.589 Å, Figure 5B) within the first 60 min of TOS (to a lesser extent in Ni_70_Ga_30_/SiO_2_ and Ni_75_Ga_25_/SiO_2_, Supporting Information Figure S24). The slight increase in the lattice parameters could be due to a temperature increase caused by the onset of the exothermic CO_2_ to methanol reaction (after the gases are switched from 20 bar N_2_ at TOS = 0 min to 20 bar CO_2_:H_2_:N_2_ = 1:3:1). It should be noted that a dealloying of the nanoparticles (i.e., oxidation of some Ga) would lead to a decrease in the lattice parameter, i.e., the opposite than what is observed experimentally.

Next, we evaluated whether changes in the oxidation state of Ga or Ni occurred under CO_2_ hydrogenation conditions. To probe possible changes in the XANES data with TOS, we calculated the difference (Δ) between the *n*th and the first XANES scan (with *n* ranging between the first and the final scan after ca. 4 h). The maximum difference in the magnitude of ΔXANES at the Ni- and Ga-K-edges was <0.05 in normalized absorption units (Figure 5C,D), indicating that no reduction nor oxidation of Ga took place under reaction conditions. This also implied that the small quantities of GaO_*x*_ that were present after activation of the catalysts remained constant with TOS. We however do not exclude the possibility of alloying/dealloying of Ga taking place over extended catalyst operation times. In line with our XANES data and the observation of a stable fcc alloy phase from operando total scattering/PDF analysis, also the fitted EXAFS parameters of the reacted catalyst Ni_65_Ga_35_/SiO_2_ remained constant with respect to the activated catalyst (i.e., within the error of the fitting, see Supporting Information Table S8). These results indicated that the Ni–Ga alloy nanoparticles retained their structure and composition under CO_2_ hydrogenation conditions, with no evidence for dealloying/additional alloying, in agreement with the study of Hejral et al. using unsupported Ni–Ga nanoparticles.^25^ The behavior observed for Ni–Ga/SiO_2_ contrasts what have been observed for other bimetallic catalysts (i.e., Cu–Zn,^31^ Cu–Ga,^10,12^ Pd–Ga^18^) synthesized via the same SOMC approach used in this work, where dealloying, even if partial, was always observed under CO_2_ hydrogenation conditions.^12,18,31,59,60^ Note that the absence of bulk dealloying was also observed for the Au–Zn/SiO_2_ system (Au:Zn ca. 33:67).^61^ Also here, the Au–Zn alloy formed after catalyst activation (H_2_, 300 °C) and remained stable during CO_2_ hydrogenation conditions (10 bar CO_2_:H_2_:Ar = 1:3:1, 230 °C), while a minor oxidation of surface Zn was observed using operando XAS with a modulation of the gas-phase composition.

### 2.5 Surface Reaction Intermediates and Bound Adsorbates

To assess whether differences in the catalysts’ structure and composition (e.g., Ni:Ga ratio in the fcc alloy and quantity of GaO_*x*_) affect the type and amounts of surface adsorbate species and reaction intermediates, we performed operando DRIFTS experiments on Ni_65_Ga_35_/SiO_2_, Ni_70_Ga_30_/SiO_2_, and Ni_75_Ga_25_/SiO_2_, i.e., catalysts with distinctive catalytic performance and structure. It is worth remembering that Ni_65_Ga_35_/SiO_2_ and Ni_70_Ga_30_/SiO_2_ contained fcc alloy nanoparticles with an identical Ni:Ga ratio (75:25) but different quantities of GaO_*x*_ (0.14 and 0.06  mol_Ni_^–1^ in Ni_65_Ga_35_/SiO_2_ and Ni_70_Ga_30_/SiO_2_, respectively) allowing us to assess the role of GaO_*x*_ species on the observed surfaces species. Further, Ni_75_Ga_25_/SiO_2_ contained an fcc alloy with a higher Ni:Ga ratio (82:12) compared to Ni_65_Ga_35_/SiO_2_ and Ni_70_Ga_30_/SiO_2_. Importantly, online analysis of the off-gas during the operando DRIFTS experiments reproduced the trend of the methanol formation rate observed in the catalytic packed bed measurements (Supporting Information Figures S46–48), viz., the methanol formation rate decreases in the order Ni_65_Ga_35_/SiO_2_ > Ni_70_Ga_30_/SiO_2_ > Ni_75_Ga_25_/SiO_2_.

In the operando DRIFTS data acquired, there are two spectral regions of interest (i) 1900–2200 cm^–1^ and (ii) 2600–3200 cm^–1^, in which *M*_*n*_-CO_m_ (M = Ni,Ga) vibrations^62^ and C–H stretching vibrations^63^ are expected, respectively. Bands at ca. 2200–2450, 3500, and 3770 cm^–1^ are assigned to gaseous CO_2_(*g*) (see Table S10 in the Supporting Information for all the referenced band assignments).

In the region 1900 cm^–1^ - 2200 cm^–1^ (Supporting Information Figures S46–48), bands at 2077, 2094, and 2129 cm^–1^ were observed for all of the catalysts and were due to pressurized gaseous CO_2_(*g*).^64^ The first and most prominent band to appear after less than 10 min once the reaction gas mixtures has been introduced into the IR cell (range of 2049–2064 cm^–1^) could be assigned to Ni(CO)_*n*_ (*n* = 1–4) surface adsorbate species and possibly also to gaseous Ni(CO)_4_*(g)*.^24,65−67^

**Figure 6:**
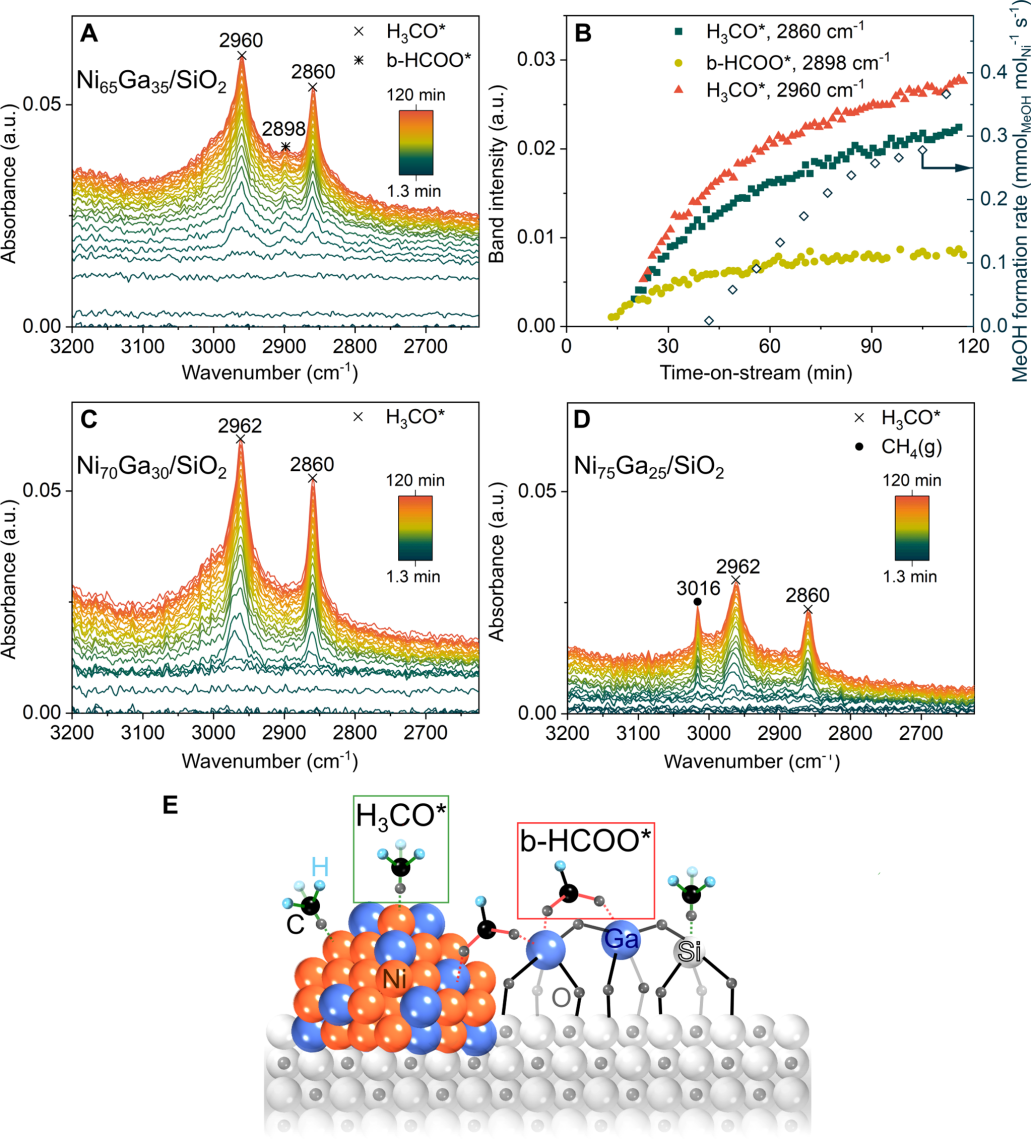
(A) Operando DRIFTS for Ni_65_Ga_33_/SiO_2_ and (B) the corresponding baseline subtracted, intensity of the methoxy (H_3_CO*) and bidentate formate (b-HCOO*) bands and the measured methanol formation rate as a function of TOS. Operando DRIFTS for (C) Ni_70_Ga_30_/SiO_2_ and (D) Ni_75_Ga_25_/SiO_2_. TOS denotes the time after switching from 20 bar N_2_ to reaction conditions. Conditions: 20 bar, CO_2_:H_2_:N_2_ = 1:3:1, 230 °C, GHSV = 60 L g_cat_^–1^ h^–1^. (E) Schematic showing how methoxy and formate adsorbate species are potentially adsorbed on the catalyst surface.

In the region 2600–3200 cm^–1^, two bands due to C–H stretching vibrations at 2860 and 2960–2962 cm^–1^ appeared on all catalysts independent of their composition. These bands started to appear at ca. 20–30 min after the introduction of a reaction gas mixture (Figure 6A–D). Previous studies on supported metal nanoparticle catalysts for methanol synthesis have assigned these bands to the asymmetric and symmetric stretching modes of methoxy groups (−OCH_3_) bonded to either the metal nanoparticles or the support.^63^ The band positions observed in our study (2860 and 2960–2962 cm^–1^) matched well with values reported for methoxy species adsorbed onto SiO_2_.^68−70^ Interestingly, on Ni_65_Ga_35_/SiO_2_ (i.e., the most active and selective catalyst), an additional band at 2898–2900 cm^–1^ was observed that can be assigned to ν(CH) of bidentate formate (b-HCOO);^18^ this band is absent in the other two catalysts, i.e., Ni_70_Ga_30_/SiO_2_ and Ni_75_Ga_25_/SiO_2_. However, we do not exclude the presence of formate species in these catalysts, which could be present in a smaller extent compared to that of Ni_65_Ga_35_/SiO_2_ and are thus not detected and overshadowed with overlapping methoxy species in the IR spectrum. Note that two additional bands due to b-HCOO would be expected in the region 2800–3000 cm^–1^, which, however, overlap with the two bands assigned to methoxy species.^18,71,72^ Based on previous in situ IR studies on gallium oxides, the band at 2898–2900 cm^–1^ was likely due to b-HCOO on gallium oxide species,^63,73^ which was in agreement with our d-PDF and XAS analyses that indicated more abundant GaO_*x*_ species (= binding sites of b-HCOO) on Ni_65_Ga_35_/SiO_2_ when compared to that on Ni_70_Ga_30_/SiO_2_ and Ni_75_Ga_25_/SiO_2_. Hence, our experiments suggested that GaO_*x*_ species (likely in proximity of the alloy nanoparticles) further promote the formation of formate species. According to the work of Collins et al.,^73^ bidentate formate species on gallium oxide also shows bands in the region 1300–1600 cm^–1^ (i.e., ν_as_(CO_2_) at 1580 cm^–1^, δ(CH) at 1386 cm^–1^, ν_s_(CO_2_) at 1372 cm^–1^) which, however, could not be observed unequivocally in our system due to the strong absorption of the incident IR radiation by SiO_2_ in this spectral region, leading to a poor signal-to-noise ratio.^74^ The presence of formate (in Ni_65_Ga_35_/SiO_2_) and methoxy bands in Ni_65_Ga_35_/SiO_2_, Ni_70_Ga_30_/SiO_2_, and Ni_75_Ga_25_/SiO_2_ was in line with the previously proposed mechanism for methanol formation, i.e., the formate–methoxy pathway (Figure 6E)^24^ and in line with what has been proposed for Cu.^75^ In addition, and in agreement with the formation of some methane in Ni_75_Ga_25_/SiO_2_ (Figure 6D), operando DRIFTS on Ni_75_Ga_25_/SiO_2_ showed the *v*_3_ stretching vibration of CH_4_(*g*) at 3016 cm^–1^, along with weak rotational bands of CH_4_ in the range 2600–3200 cm^–1^ (Supporting Information Figure S48). No bands due to CH_4_ were observed on Ni_65_Ga_35_/SiO_2_ and Ni_70_Ga_30_/SiO_2_, i.e., catalysts that also did not show any methane formation or only negligible quantities in the packed bed experiments (Figure 6 A,C, Supporting Information Figures S46 and S47).

## 3 Conclusions

In this study, we report the synthesis of model catalysts containing Ni–Ga nanoparticles supported on silica (Ni_*x*_Ga_(100–*x*)_/SiO_2_) using a SOMC/TMP approach enabling a precise control over loading and particle size and yielding highly active Ni–Ga-based catalysts for the selective hydrogenation of CO_2_ into methanol, in contrast to pure Ni that favors methanation. Combined electron microscopy, operando d-PDF, and XAS studies showed that these silica-supported catalysts contained, after their in situ activation in H_2_, ∼2 nm sized, Ni–Ga fcc alloy particles with a narrow size distribution along with a small fraction of GaO_*x*_ species. Furthermore, Ni K-edge XAS showed that the electronic structure of Ni was affected by alloying and can be described as Ni^δ−^Ga^δ+^. Using Vegard’s law, the composition of the alloyed nanoparticles was determined as Ni:Ga = 75:25 in activated Ni_65_Ga_35_/SiO_2_ and Ni_70_Ga_30_/SiO_2_, whereas the alloy in Ni_75_Ga_25_/SiO_2_ was more Ni-rich (Ni:Ga = 82:12). Furthermore, considering the total Ga content in the materials, as determined by elemental analysis, each catalyst also contained residual GaO_*x*_. Notably, the GaO_*x*_ content in Ni_65_Ga_35_/SiO_2_ (0.14  mol_Ni_^–1^) was more than double than that in Ni_70_Ga_30_/SiO_2_ (0.06  mol_Ni_^–1^), despite having an alloy with the same composition. Ni_75_Ga_25_/SiO_2_, having a more Ni-rich alloy, also contained GaO_*x*_ (0.08  mol_Ni_^–1^), the quantity of which lies in between that of the other two catalysts.

Regarding catalysis, alloying Ni with Ga decreased its methanation activity, while promoting methanol formation rates up to a certain Ga content (Ni:Ga = 75:25). Ni_65_Ga_35_/SiO_2_, containing an alloy with a Ni:Ga ratio of 75:25 and the largest amount of GaO_*x*_, showed the highest methanol activity (1.1 mmol_MeOH_ mol_Ni_^–1^ s^–1^) and selectivity (S_MeOH_ = 54%) and improved performance compared to Ni_70_Ga_30_/SiO_2_ (0.6 mmol_MeOH_ mol_Ni_^–1^ s^–1^ and S_MeOH_ = 48%) which contained an alloy with the same composition (Ni:Ga = 75:25) but a lower GaO_*x*_ content. Ni_75_Ga_25_/SiO_2_, containing a Ni-richer alloy and an intermediate amount of GaO_*x*_, showed the poorest performance (0.2 mmol_MeOH_ mol_Ni_^–1^ s^–1^ and S_MeOH_ = 33%) along with the monometallic Ni catalyst (0.2 mmol_MeOH_ mol_Ni_^–1^ s^–1^ and S_MeOH_ = 7%) that favored methanation ( = 88%), as expected for pure Ni. Hence, both the amount of Ga in the alloy (Ni:Ga = 75:25) and Ga in the form of GaO_*x*_ are important for the methanol production rate and selectivity. Importantly, operando d-PDF and XAS analyses of the catalysts under CO_2_ hydrogenation conditions revealed that the fcc structure of the alloy and the oxidation states of Ni and Ga remained invariant with respect to the activated state (treated under H_2_), indicating that the bulk alloy was not affected by the reaction conditions, in sharp contrast to what has been observed for Cu–Ga and Pd–Ga. Furthermore, monitoring the catalysts by operando DRIFTS indicates that the presence of GaO_*x*_ helps in stabilizing formate species, an important reaction intermediate in the methanol formation pathway, further supporting the importance of GaO_*x*_.

Overall, our results showed that alloy nanoparticles with a Ni:Ga ratio of 75:25 result in high methanol activity and selectivity—considerably higher than for Ni-richer alloys—while the presence of GaO_*x*_ further increases the rate of methanol formation. These results pointed to the importance of site-isolation of Ni by Ga that leads to modifications of the electronic structure of Ni, viz., Ni^δ+^Ga^δ−^. Ni site isolation likely prevents methanation activity, while the presence of GaO_*x*_ further promotes the selective formation of methanol. Therefore, the regulation of the quantity of both alloyed Ga and GaO_*x*_ species is crucial in achieving a high methanol selectivity and a high rate of methanol formation.

## 4 Experimental Section

### 4.1 Materials

All preparation and operations were performed in a M. Braun glovebox under an argon atmosphere or using high vacuum and standard Schlenk techniques. Pentane was purged with argon for 30 min and dried using a MB SPS 800 solvent purification system in which columns used for pentane purification were packed with activated copper and alumina. Benzene was either distilled from purple Na^0^/benzophenone or obtained from the MB SPS system and used without further purification. All solvents were stored over 4 Å molecular sieves after being transferred into a glovebox. Four Å molecular sieves were activated under high vacuum overnight at 300 °C. Fine quartz wool (Elemental Microanalysis) was calcined at 800 °C for 12 h and transferred into a glovebox for storage. SiO_2–700_ was prepared by heating Aerosil (200 m^2^/g) to 500 °C (ramp of 300 °C/h) in air and subsequent calcination in air for 12 h. Afterward, the material was evacuated at high vacuum (10^–5^ mbar) at 500 °C for 8 h, followed by heating to 700 °C (ramp of 60 °C/h) and keeping the material at 700 °C for approximately 24 h. Titration of SiO_2–700_ using [Mg(CH_2_Ph)_2_(THF)_2_] purified via sublimation prior to use yielded an Si–OH density of 0.3 mmol/g, corresponding to 0.9 accessible Si–OH groups per nm^2^. The molecular complexes [Ga(OSi(OtBu)_3_)_3_(THF)] and [Ni(CH_3_)_2_(tmeda)] were prepared according to adapted literature procedures.^36,76^ Other reagents were purchased from Sigma-Aldrich or Acros Organics and used as received. The supported species Ga^III^/SiO_2_ (precursor for Ni_*x*_Ga_(100–*x*)_/SiO_2_ syntheses) were prepared according to an adapted literature procedure (see below).^36^

### 4.2 Synthesis of Ga^III^/SiO_2_

In a typical synthesis, SiO_2–700_ (0.729 g, 0.219 mmol −OH) was added to a 20 mL vial. Next, benzene (about 5 mL) was added slowly while stirring to give a white suspension. [Ga(OSi(OtBu)_3_)_3_(THF)] (0.147 mmol/g SiO_2–700_ nominal loading; 0.100 g, 0.107 mmol) was added slowly to the suspension as a white solution in benzene (about 5 mL) while stirring (1200 rpm). The resulting suspension was stirred at room temperature (rt) for 12 h (100 rpm). The benzene on top of the silica material was decanted, and the material was washed with benzene (5 mL) two times to wash off any unreacted complex. The material was then washed with pentane before it was dried in vacuo for 2 h to remove any residual solvent yielding Ga^III^/SiO_2_ as a white solid. The white material was then transferred into a tubular quartz reactor. The reactor was set under high vacuum (10^–5^ mbar) and successively heated to 300 °C (ramp of 5 °C/min) for 1 h, 400 °C (ramp of 5 °C/min) for 1 h, 500 °C (ramp of 5 °C/min) for 1 h, and finally 600 °C (ramp of 5 °C/min) for 10 h, yielding Ga^III^/SiO_2_ as a white/gray material.

### 4.3 Synthesis of Ga_100_/SiO_2_

Here, ca. 0.580 g of Ga^III^/SiO_2_ (synthesized as described above) was added to a tubular quartz flow-reactor supported with a porous quartz frit. The reactor was heated to 600 °C (ramp of 5 °C/min) under a steady flow of H_2_ and then treated under H_2_ at 600 °C for 12 h. The reactor was subsequently evacuated under high vacuum (10^–5^ mbar) while cooling to room temperature, yielding Ga_100_/SiO_2_ as a white/gray material. Elemental analysis yielded: Ga, 0.88 wt %.

### 4.4 Synthesis of Ni_100_/SiO_2_

SiO_2–700_ (0.726 g, 0.218 mmol −OH) was added to a 20 mL vial. Benzene (about 5 mL) was added slowly while stirring to give a white suspension. [Ni(CH_3_)_2_(tmeda)] (0.342 mmol/g SiO_2–700_ nominal loading; 0.051 g, 0.248 mmol) was added slowly to the suspension as a deep yellow solution in benzene (about 5 mL) while stirring (1200 rpm), resulting in an immediate pink/deep-red coloration of SiO_2_. The resulting suspension was stirred at room temperature for 75 min (125 rpm) after which no yellow color of the supernatant was observable anymore. The benzene on top of the silica material was decanted, and the material was washed with benzene (5 mL) two times to wash off any unreacted complex. The material was then washed with pentane before it was dried in vacuo for 30 min to remove any residual solvent, yielding Ni^II^/SiO_2_ as a pink/deep-red solid. The material was transferred to a tubular quartz flow-reactor supported with a porous quartz frit. The reactor was heated to 600 °C (ramp of 5 °C/min) under a steady flow of H_2_ and held under H_2_ at 600 °C for 12 h. The reactor was subsequently evacuated under high vacuum (10^–5^ mbar) while cooling to room temperature, yielding Ni_100_/SiO_2_ as a dark brown/black material. Elemental analysis yielded: Ni, 2.12 wt %.

### 4.5 Synthesis of Ni_*x*_Ga_(100–*x*)_/SiO_2_ (*x* = 65, 70, 75)

Ga^III^/SiO_2_ materials (0.700, 0.707, and 0.704 g) were synthesized as described above with different nominal Ga loadings (0.114, 0.147, and 0.184 mmol/g SiO_2–700_ nominal loading; 0.074, 0.097, and 0.121 g of [Ga(OSi(OtBu)_3_)_3_(THF)]). All materials were obtained as off white/gray materials. In a next step, the Ga^III^/SiO_2_ materials (0.640, 0.605, and 0.545 g) were added to 20 mL vials. Next, benzene (about 5 mL) was added slowly while stirring to give a grayish suspension. [Ni(CH_3_)_2_(tmeda)] (0.342 mmol/g Ga^III^/SiO_2_ nominal loading; 0.045, 0.042, and 0.038 g; 0.219, 0.207, and 0.187 mmol) was added slowly to the suspension as a deep yellow solution in benzene (about 5 mL) while stirring (1200 rpm), resulting in an immediate pink/deep-red coloration of the SiO_2_. The resulting suspensions were stirred at room temperature for 65 min (125 rpm) after which no yellow color of the supernatant was observable anymore. The benzene on top of the silica material was decanted, and the materials were washed with benzene (5 mL) two times to wash off any unreacted complex. The materials were then washed with pentane before they were dried in vacuo for ca. 45 min to remove any residual solvent yielding pink/deep-red solids. The materials were then transferred to small, tubular quartz vessels supported with a porous quartz frit. These small tubes were plugged with a small amount of quartz wool to avoid spilling. The small vessels were subsequently transferred to a large tubular quartz flow-reactor supported with a porous quartz frit. The reactor was heated to 600 °C (ramp of 5 °C/min) under a steady flow of H_2_ and then treated under H_2_ at 600 °C for 12 h. The reactor was subsequently evacuated under high vacuum (10^–5^ mbar) while cooling to room temperature, yielding Ni_75_Ga_25_/SiO_2_, Ni_70_Ga_30_/SiO_2_, and Ni_65_Ga_35_/SiO_2_ as dark brown/black materials.

The synthesis yielded ca. 500 mg per sample. The materials were characterized by elemental analysis, catalytic CO_2_ hydrogenation tests, in situ and operando X-ray total scattering, and operando DRIFTS. A second batch of Ni_65_Ga_35_/SiO_2_ and Ni_75_Ga_25_/SiO_2_ catalysts was prepared to allow for further characterization. The second catalyst batch was characterized by XAS (Figures 3, S32–44), ICP, catalytic tests (labeled with the suffix XAS). To ensure reproducibility, PDF was also collected for Ni_65_Ga_35_/SiO_2_-XAS.

### 4.6 Characterization

#### 4.6.1 Elemental Analysis (Ga, Ni)

The elemental composition of the catalysts was determined by ICP-OES using an Agilent 5100 VDV instrument. Typically, 2–3 mg of the sample was dissolved in 5 mL of aqua regia, followed by microwave digestion at 175 °C for 30 min (Anton Paar, Multiwave GO). The resulting solution was cooled down to room temperature and diluted to 25 mL with deionized water. For the calibration of the instrument, a multielement standard (multielement standard solution 5, Sigma-Aldrich) was used. Each measurement was repeated three times, and the average values are reported in Table S1.

#### 4.6.2 Transmission Electron Microscopy

HAADF-STEM images were recorded on a FEI Talos F200X and–where indicated–a JEOL JEM-ARM300F Grand Arm “Vortex” instrument operated at 200 and 300 keV, respectively. Powdered samples were mixed in solid form with a Lacey-C 400 mesh Cu grid inside a glovebox under an Ar atmosphere before it was mounted onto a vacuum transfer tomography holder from Fischione Instruments (model #2560) (Talos) or a Double Tilt Atoms Defend Holder System (Mel-Build Corporation, serial number: DT-TR-006-J001) inside the glovebox, which was subsequently transferred to the chamber of the TEM in the absence of air. All imaging was done in air-free conditions if not indicated otherwise. For all materials, 300 nanoparticles were counted to yield a particle size distribution (PSD) analysis. The determination of the nanoparticle diameters for the PSD was done by manual measurements using the software ImageJ (version 1.52a). All values extracted from the specific particle size distributions assumed a normal distribution (most of the shown distribution curves are log-normal). The “±” symbol in the particle size distributions plots indicates the standard deviation of the mean particle size. The following catalysts were characterized by HAADF-STEM/EDX: Ni_100/_SiO_2_, Ni_75_Ga_25_/SiO_2_, Ni_70_Ga_30_/SiO_2_, and Ni_65_Ga_35_/SiO_2_ (as-prepared and after a CO_2_ hydrogenation test, the latter are referred to as “spent”).

#### 4.6.3 Total X-ray Scattering and X-ray Absorption Spectroscopy

In situ/operando X-ray total scattering and XAS experiments were performed at the beamlines ID15A and BM31 of the European Synchrotron Radiation Facility, respectively. The same setup was used for both types of experiments, consisting of a capillary cell reactor (quartz capillary with an outer diameter of 1 and 0.02 mm wall thickness) containing ca. 2–3 mg of catalyst placed between two quartz wool plugs. Heating of the capillary was achieved from below via a hot air blower, and gases could be flown through it at a defined pressure via mass flow controllers and a backpressure regulator placed after the outlet of the capillary. A typical in situ/operando experiment consists of an in situ activation treatment (temperature ramp to 600 °C at 10 °C/min in 1 bar 5 mL/min H_2_ and then waiting for 1 h), cooling down to the reaction temperature of 230 °C in 1 bar 5 mL/min H_2_ (or 50 °C, to collect EXAFS, see Figure S18), and then pressurizing the reactor to 20 bar in 15 mL/min N_2_ (at 230 °C). Once the pressure had equilibrated, the gases were changed to the reaction gas mix of CO_2_:H_2_:N_2_ = 1:3:1 (at 20 bar, 5 mL/min). The off-gas during the reaction was analyzed using a compact gas chromatograph (Global Analyzer Solutions compact GC^4.0^ equipped with FID and TCD detectors, 1 injection/7 min).

X-ray total scattering data was collected continuously at a rate of 1 measurement/2.62 min and up to *Q*_max,instr_ = 30 Å^–1^ (incident X-ray energy of 90.0 keV). Total scattering data of the pristine silica support was measured under in situ activation and reaction conditions and used as background to calculate the d-PDF data of the Ni_*x*_Ga_(100–*x*)_/SiO_2_ (Supporting Information Figure S22). In addition, total scattering data of the CeO_2_ NIST reference materials were obtained to determine the experimental resolution parameters *Q*_damp_ and *Q*_broad_. For the conversion from total scattering data (reciprocal space) to the PDF (real space), the software PDFgetX3^77^ v. 2.2.1 was used, whereas the modeling of the d-PDF was done in PDFGui^78^ v 1.0. The total scattering data was processed within the range Q_min_ = 1.5 Å^-1^ and Q_max_ = 23 Å^-1^ with r_poly_ = 1.0, which is approximately the r-limit for the maximum frequency in the F(Q) correction polynomial.

Ni and Ga K-edge XAS scans were collected consecutively. Ni and Ga XANES were collected between 8250 and 8550 eV and 10300–10600 eV, respectively, with a step of 0.3 eV (ca. 50 s/spectrum). Ni and Ga EXAFS were collected between 8200 and 8970 eV and 10200–11100 eV with a step of 0.5 eV (ca. 3 min/spectrum). More details on the X-ray total scattering and XAS data collection and processing can be found in section 3 of the Supporting Information.

#### 4.6.4 Diffuse Reflectance Infrared Fourier Transform Spectroscopy

Operando DRIFTS experiments were performed using a Nicolet 6700 FT-IR equipped with a Harrick Praying Mantis DRIFTS accessory and high-temperature reaction chamber. Data were collected from 650 to 4000 cm^–1^ at a spectral resolution of 4 cm^–1^ using a mercury cadmium telluride detector. In a typical experiment, ca. 20 mg of powder was placed onto a piece of quartz wool in the sample cup of the reaction cell. First, the catalysts were in situ activated in 15% H_2_/N_2_ (20 mL/min) at 590 °C (10°/min) for 1 h and subsequently cooled down to 230 °C. Next, the cell was pressurized to 20 bar in N_2_ (20 mL/min). Once the pressure had stabilized, a measurement was collected, which served as the background for all the subsequent measurements under reaction conditions. In a next step, the atmosphere was switched to CO_2_:H_2_:N_2_ = 1:3:1 (at 20 bar, 20 mL/min), and measurements were continuously collected every 1.3 min for ca. 2 h. The identical compact GC as the one used for the operando XAS/PDF experiments was used for the analysis of the off-gas. A blank test (empty reaction cell) was performed to determine the time needed for the equilibration of the feed gases from the time when the gases are switched from 20 bar N_2_ to the reaction gas mixture. The exchange time was approximately 126 min (see Figure S45).

### 4.7 Catalytic Tests

For the catalytic tests, 100 mg of the as-prepared powder was transferred into a Hastelloy C276 reactor tube (internal diameter 9.1 mm) inside a M. Braun glovebox under a N_2_ atmosphere. The powder was placed in between two pieces of quartz wool (Acros Organics, 9–30 μm) and onto a frit in the middle of the reactor tube (Hastelloy C276, pore size 2 μm). The reactor was then transferred from the glovebox and mounted into a Microactivity-Efficient flow reactor system (PID Eng & Tech), without exposing the catalyst to air (the reactor is provided with valves for such purpose). The spent catalysts were transferred back into the glovebox and stored for further TEM analysis.

The CO_2_ hydrogenation tests were performed as follows. First, the catalyst was heated to 600 °C (10 °C/min) in 1 bar H_2_ (50 mL/min) and held at 600 °C for 1 h. This step is referred to as in situ activation. Subsequently, the catalyst was cooled down to the reaction temperature of 230 °C (still in 1 bar H_2_, 50 mL/min), followed by a switch of the gas flow to 80 mL/min N_2_ and a pressure increase to 25 bar. After the stabilization of the pressure and temperature, the gas feed was switched to 25 bar of CO_2_:H_2_:N_2_ = 1:3:1 (100 mL/min, resulting in a GHSV of 60 L gcat^–1^ h^–1^), and the off-gas was continuously analyzed by a gas chromatograph (PerkinElmer Clarus 580 equipped with FID and TCD detectors, 1 injection/30 min). For further information on the catalytic tests, we refer the reader to Section 2 of the Supporting Information.

## Supporting Information Available

Experimental details, additional microscopy, X-ray absorption and IR spectroscopy, as well as X-ray total scattering/PDF and catalytic test data (PDF)

## Abbreviations

d-PDF: differential (or difference) pair distribution function analysis
DRIFTS: diffuse reflectance infrared Fourier transform spectroscopy
EXAFS: extended X-ray absorption fine structure
fcc: face-centered cubic
SOMC: surface organometallic chemistry
STEM-EDX: scanning transmission electron microscopy–element-dispersive X-ray spectroscopy
TMP: thermolytic molecular precursor
TOS: time-on-stream
XANES: X-ray absorption near-edge structure
XAS: X-ray absorption spectroscopy

## References

[ref1] Zhong J.; Yang X.; Wu Z.; Liang B.; Huang Y.; Zhang T. State of the Art and Perspectives in Heterogeneous Catalysis of CO_2_ Hydrogenation to Methanol. Chem. Soc. Rev. 2020, 49 (5), 1385–1413. 10.1039/C9CS00614A. 32067007

[ref2] Sha F.; Han Z.; Tang S.; Wang J.; Li C. Hydrogenation of Carbon Dioxide to Methanol over Non–Cu-Based Heterogeneous Catalysts. ChemSusChem 2020, 13 (23), 6160–6181. 10.1002/cssc.202002054. 33146940

[ref3] Prieto G.; Zečević J.; Friedrich H.; de Jong K. P.; de Jongh P. E. Towards Stable Catalysts by Controlling Collective Properties of Supported Metal Nanoparticles. Nat. Mater. 2013, 12 (1), 34–39. 10.1038/nmat3471. 23142841

[ref4] Liang B.; Ma J.; Su X.; Yang C.; Duan H.; Zhou H.; Deng S.; Li L.; Huang Y. Investigation on Deactivation of Cu/ZnO/Al_2_O_3_ Catalyst for CO_2_ Hydrogenation to Methanol. Ind. Eng. Chem. Res. 2019, 58 (21), 9030–9037. 10.1021/acs.iecr.9b01546.

[ref5] Martin O.; Pérez-Ramírez J. New and Revisited Insights into the Promotion of Methanol Synthesis Catalysts by CO_2_. Catal. Sci. Technol. 2013, 3 (12), 3343–3352. 10.1039/c3cy00573a.

[ref6] Jangam A.; Hongmanorom P.; Hui Wai M.; Jeffry Poerjoto A.; Xi S.; Borgna A.; Kawi S. CO_2_ Hydrogenation to Methanol over Partially Reduced Cu-SiO_2P_ Catalysts: The Crucial Role of Hydroxyls for Methanol Selectivity. ACS Appl. Energy Mater. 2021, 4 (11), 12149–12162. 10.1021/acsaem.1c01734.

[ref7] Liao F.; Lo T. W. B.; Tsang S. C. E. Recent Developments in Palladium-Based Bimetallic Catalysts. ChemCatChem. 2015, 7 (14), 1998–2014. 10.1002/cctc.201500245.

[ref8] Li M. M.-J.; Tsang S. C. E. Bimetallic Catalysts for Green Methanol Production via CO_2_ and Renewable Hydrogen: A Mini-Review and Prospects. Catal. Sci. Technol. 2018, 8 (14), 3450–3464. 10.1039/C8CY00304A.

[ref9] Lee S. W.; Luna M. L.; Berdunov N.; Wan W.; Kunze S.; Shaikhutdinov S.; Cuenya B. R. Unraveling Surface Structures of Gallium Promoted Transition Metal Catalysts in CO_2_ Hydrogenation. Nat. Commun. 2023, 14 (1), 4649 10.1038/s41467-023-40361-3. 37532720 PMC10397205

[ref10] Alfke J. L.; Tejeda-Serrano M.; Gani T. Z. H.; Rochlitz L.; Zhang S. B. X. Y.; Lin L.; Copéret C.; Safonova O. V. Boundary Conditions for Promotion versus Poisoning in Copper-Gallium-based CO_2_–to–Methanol Hydrogenation Catalysts. ChemRxiv Submitted 2023–06–06. https://chemrxiv.org/engage/chemrxiv/article-details/647d9a52be16ad5c57800181 (accessed December 11, 2023), DOI: 10.26434/chemrxiv-2023-47d99.

[ref11] Medina J. C.; Figueroa M.; Manrique R.; Pereira J. R.; Srinivasan P. D.; Bravo-Suárez J. J.; Medrano V. G. B.; Jiménez R.; Karelovic A. Catalytic Consequences of Ga Promotion on Cu for CO_2_ Hydrogenation to Methanol. Catal. Sci. Technol. 2017, 7 (15), 3375–3387. 10.1039/C7CY01021D.

[ref12] Lam E.; Noh G.; Wing Chan K.; Larmier K.; Lebedev D.; Searles K.; Wolf P.; Safonova O. V.; Copéret C. Enhanced CH_3_OH Selectivity in CO_2_ Hydrogenation Using Cu-Based Catalysts Generated via SOMC from Ga III Single-Sites. Chem. Sci. 2020, 11 (29), 7593–7598. 10.1039/D0SC00465K. 34094136 PMC8159433

[ref13] Studt F.; Sharafutdinov I.; Abild-Pedersen F.; Elkjær C. F.; Hummelsho̷j J. S.; Dahl S.; Chorkendorff I.; No̷rskov J. K. Discovery of a Ni-Ga Catalyst for Carbon Dioxide Reduction to Methanol. Nat. Chem. 2014, 6 (4), 320–324. 10.1038/nchem.1873. 24651199

[ref14] Gallo A.; Snider J. L.; Sokaras D.; Nordlund D.; Kroll T.; Ogasawara H.; Kovarik L.; Duyar M. S.; Jaramillo T. F. Ni_5_Ga_3_ Catalysts for CO_2_ Reduction to Methanol: Exploring the Role of Ga Surface Oxidation/Reduction on Catalytic Activity. Appl. Catal., B 2020, 267, 118369 10.1016/j.apcatb.2019.118369.

[ref15] Tang Q.; Ji W.; Russell C. K.; Zhang Y.; Fan M.; Shen Z. A New and Different Insight into the Promotion Mechanisms of Ga for the Hydrogenation of Carbon Dioxide to Methanol over a Ga-Doped Ni(211) Bimetallic Catalyst. Nanoscale 2019, 11 (20), 9969–9979. 10.1039/C9NR01245A. 31070648

[ref16] Sharafutdinov I.; Elkjær C. F.; Pereira de Carvalho H. W.; Gardini D.; Chiarello G. L.; Damsgaard C. D.; Wagner J. B.; Grunwaldt J.-D.; Dahl S.; Chorkendorff I. Intermetallic Compounds of Ni and Ga as Catalysts for the Synthesis of Methanol. J. Catal. 2014, 320, 77–88. 10.1016/j.jcat.2014.09.025.

[ref17] Choi H.; Oh S.; Trung Tran S. B.; Park J. Y. Size-Controlled Model Ni Catalysts on Ga_2_O_3_ for CO_2_ Hydrogenation to Methanol. J. Catal. 2019, 376, 68–76. 10.1016/j.jcat.2019.06.051.

[ref18] Docherty S. R.; Phongprueksathat N.; Lam E.; Noh G.; Safonova O. V.; Urakawa A.; Copéret C. Silica-Supported PdGa Nanoparticles: Metal Synergy for Highly Active and Selective CO_2_-to-CH_3_OH Hydrogenation. JACS Au 2021, 1 (4), 450–458. 10.1021/jacsau.1c00021. 34467307 PMC8395611

[ref19] Collins S. E.; Delgado J. J.; Mira C.; Calvino J. J.; Bernal S.; Chiavassa D. L.; Baltanas M. A.; Bonivardi A. L. The Role of Pd-Ga Bimetallic Particles in the Bifunctional Mechanism of Selective Methanol Synthesis via CO_2_ Hydrogenation on a Pd/Ga_2_O_3_ Catalyst. J. Catal. 2012, 292, 90–98. 10.1016/j.jcat.2012.05.005.

[ref20] Fiordaliso E. M.; Sharafutdinov I.; Carvalho H. W. P.; Grunwaldt J.-D.; Hansen T. W.; Chorkendorff I.; Wagner J. B.; Damsgaard C. D. Intermetallic GaPd_2_ Nanoparticles on SiO_2_for Low-Pressure CO_2_ Hydrogenation to Methanol: Catalytic Performance and In Situ Characterization. ACS Catal. 2015, 5 (10), 5827–5836. 10.1021/acscatal.5b01271.

[ref21] García-Trenco A.; White E. R.; Regoutz A.; Payne D. J.; Shaffer M. S. P.; Williams C. K. Pd_2_Ga-Based Colloids as Highly Active Catalysts for the Hydrogenation of CO_2_ to Methanol. ACS Catal. 2017, 7 (2), 1186–1196. 10.1021/acscatal.6b02928.

[ref22] Collins S. E.; Baltanás M. A.; Delgado J. J.; Borgna A.; Bonivardi A. L. CO_2_ Hydrogenation to Methanol on Ga_2_O_3_-Pd/SiO_2_ Catalysts: Dual Oxide-Metal Sites or (Bi)Metallic Surface Sites?. Catal. Today 2021, 381, 154–162. 10.1016/j.cattod.2020.07.048.

[ref23] Vogt C.; Groeneveld E.; Kamsma G.; Nachtegaal M.; Lu L.; Kiely C. J.; Berben P. H.; Meirer F.; Weckhuysen B. M. Unravelling Structure Sensitivity in CO_2_ Hydrogenation over Nickel. Nat. Catal. 2018, 1 (2), 127–134. 10.1038/s41929-017-0016-y.

[ref24] Rasteiro L. F.; De Sousa R. A.; Vieira L. H.; Ocampo-Restrepo V. K.; Verga L. G.; Assaf J. M.; Da Silva J. L. F.; Assaf E. M. Insights into the Alloy-Support Synergistic Effects for the CO_2_ Hydrogenation towards Methanol on Oxide-Supported Ni_5_Ga_3_ Catalysts: An Experimental and DFT Study. Appl. Catal., B 2022, 302, 120842 10.1016/j.apcatb.2021.120842.

[ref25] Hejral U.; Timoshenko J.; Kordus D.; Lopez Luna M.; Divins N. J.; Widrinna S.; Zegkinoglou I.; Pielsticker L.; Mistry H.; Boscoboinik J. A.; Kuehl S.; Roldan Cuenya B. Tracking the Phase Changes in Micelle-Based NiGa Nanocatalysts for Methanol Synthesis under Activation and Working Conditions. J. Catal. 2022, 405, 183–198. 10.1016/j.jcat.2021.11.024.

[ref26] Tang Q.; Shen Z.; Russell C. K.; Fan M. Thermodynamic and Kinetic Study on Carbon Dioxide Hydrogenation to Methanol over a Ga_3_Ni_5_(111) Surface: The Effects of Step Edge. J. Phys. Chem. C 2018, 122 (1), 315–330. 10.1021/acs.jpcc.7b08232.

[ref27] Tang Q.; Shen Z.; Huang L.; He T.; Adidharma H.; Russell A. G.; Fan M. Synthesis of Methanol from CO_2_ Hydrogenation Promoted by Dissociative Adsorption of Hydrogen on a Ga_3_Ni_5_(221) Surface. Phys. Chem. Chem. Phys. 2017, 19 (28), 18539–18555. 10.1039/C7CP03231E. 28685170

[ref28] Cortés-Reyes M.; Azaoum I.; Molina-Ramírez S.; Herrera C.; Larrubia M. Á.; Alemany L. J. NiGa Unsupported Catalyst for CO_2_ Hydrogenation at Atmospheric Pressure. Tentative Reaction Pathways. Ind. Eng. Chem. Res. 2021, 60 (51), 18891–18899. 10.1021/acs.iecr.1c03115.

[ref29] Wencka M.; Kovač J.; Dasireddy V. D. B. C.; Likozar B.; Jelen A.; Vrtnik S.; Gille P.; Kim H. J.; Dolinšek J. The Effect of Surface Oxidation on the Catalytic Properties of Ga_3_Ni_2_ Intermetallic Compound for Carbon Dioxide Reduction. J. Anal. Sci. Technol. 2018, 9 (1), 12 10.1186/s40543-018-0144-2.

[ref30] Chiang C. L.; Lin K. S.; Lin Y. G. Preparation and Characterization of Ni_5_Ga_3_ for Methanol Formation via CO_2_ Hydrogenation. Top. Catal. 2017, 60 (9), 685–696. 10.1007/s11244-017-0771-7.

[ref31] Lam E.; Noh G.; Larmier K.; Safonova O. V.; Copéret C. CO_2_ Hydrogenation on Cu-Catalysts Generated from ZnII Single-Sites: Enhanced CH_3_OH Selectivity Compared to Cu/ZnO/Al_2_O_3_. J. Catal. 2021, 394, 266–272. 10.1016/j.jcat.2020.04.028.

[ref32] Copéret C.; Comas-Vives A.; Conley M. P.; Estes D. P.; Fedorov A.; Mougel V.; Nagae H.; Núñez-Zarur F.; Zhizhko P. A. Surface Organometallic and Coordination Chemistry toward Single-Site Heterogeneous Catalysts: Strategies, Methods, Structures, and Activities. Chem. Rev. 2016, 116 (2), 323–421. 10.1021/acs.chemrev.5b00373. 26741024

[ref33] Samantaray M. K.; Pump E.; Bendjeriou-Sedjerari A.; D’Elia V.; Pelletier J. D. A.; Guidotti M.; Psaro R.; Basset J.-M. Surface Organometallic Chemistry in Heterogeneous Catalysis. Chem. Soc. Rev. 2018, 47 (22), 8403–8437. 10.1039/C8CS00356D. 30250959

[ref34] Fujdala K. L.; Tilley T. D. Design and Synthesis of Heterogeneous Catalysts: The Thermolytic Molecular Precursor Approach. J. Catal. 2003, 216 (1), 265–275. 10.1016/S0021-9517(02)00106-9.

[ref35] Searles K.; Chan K. W.; Mendes Burak J. A.; Zemlyanov D.; Safonova O.; Copéret C. Highly Productive Propane Dehydrogenation Catalyst Using Silica-Supported Ga–Pt Nanoparticles Generated from Single-Sites. J. Am. Chem. Soc. 2018, 140 (37), 11674–11679. 10.1021/jacs.8b05378. 30145890

[ref36] Searles K.; Siddiqi G.; Safonova O. V.; Copéret C. Silica-Supported Isolated Gallium Sites as Highly Active, Selective and Stable Propane Dehydrogenation Catalysts. Chem. Sci. 2017, 8 (4), 2661–2666. 10.1039/C6SC05178B. 28553501 PMC5433511

[ref37] Zimmerli N. K.; Müller C. R.; Abdala P. M. Deciphering the Structure of Heterogeneous Catalysts across Scales Using Pair Distribution Function Analysis. Trends Chem. 2022, 4 (9), 807–821. 10.1016/j.trechm.2022.06.006.

[ref38] Lamberti C.; Borfecchia E.; van Bokhoven J. A.; Fernández-García M. XAS Spectroscopy: Related Techniques and Combination with Other Spectroscopic and Scattering Methods. In X-Ray Absorption and X-Ray Emission Spectroscopy; John Wiley & Sons, Ltd., 2016; pp 303–350.

[ref39] Geslin P.-A.; Rodney D. Microelasticity Model of Random Alloys. Part I: Mean Square Displacements and Stresses. J. Mech. Phys. Solids 2021, 153, 104479 10.1016/j.jmps.2021.104479.

[ref40] Lindahl Christiansen T.; Kjær E. T. S.; Kovyakh A.; Röderen M. L.; Ho̷j M.; Vosch T.; Jensen K. M. Ø. Structure Analysis of Supported Disordered Molybdenum Oxides Using Pair Distribution Function Analysis and Automated Cluster Modelling. J. Appl. Crystallogr. 2020, 53 (1), 148–158. 10.1107/S1600576719016832. 32047409 PMC6998784

[ref41] Christiansen T. L.; Cooper S. R.; Jensen K. M. Ø. There’s No Place like Real-Space: Elucidating Size-Dependent Atomic Structure of Nanomaterials Using Pair Distribution Function Analysis. Nanoscale Adv. 2020, 2 (6), 2234–2254. 10.1039/D0NA00120A. 36133369 PMC9418950

[ref42] Quinson J.; Kacenauskaite L.; Christiansen T. L.; Vosch T.; Arenz M.; Jensen K. M. Ø. Spatially Localized Synthesis and Structural Characterization of Platinum Nanocrystals Obtained Using UV Light. ACS Omega 2018, 3 (8), 10351–10356. 10.1021/acsomega.8b01613. 30198008 PMC6120742

[ref43] Doan-Nguyen V. V. T.; Kimber S. A. J.; Pontoni D.; Reifsnyder Hickey D.; Diroll B. T.; Yang X.; Miglierini M.; Murray C. B.; Billinge S. J. L. Bulk Metallic Glass-like Scattering Signal in Small Metallic Nanoparticles. ACS Nano 2014, 8 (6), 6163–6170. 10.1021/nn501591g. 24871305

[ref44] Castro-Fernández P.; Mance D.; Liu C.; Moroz I. B.; Abdala P. M.; Pidko E. A.; Copéret C.; Fedorov A.; Müller C. R. Propane Dehydrogenation on Ga_2_O_3_-Based Catalysts: Contrasting Performance with Coordination Environment and Acidity of Surface Sites. ACS Catal. 2021, 11, 907–924. 10.1021/acscatal.0c05009.

[ref45] Jensen K. M. Ø. Characterization of Nanomaterials with Total Scattering and Pair Distribution Function Analysis: Examples from Metal Oxide Nanochemistry. Chimia 2021, 75 (5), 368 10.2533/chimia.2021.368. 34016231

[ref46] Suryanarayana C.; Norton M. G. X-Rays and Diffraction. In X-Ray Diffraction: A Practical Approach; Suryanarayana C.; Norton M. G., Eds.; Springer US: Boston, MA, 1998; pp 3–19.

[ref47] Vegard L. Die Konstitution der Mischkristalle und die Raumfüllung der Atome. Z. Physik 1921, 5 (1), 17–26. 10.1007/BF01349680.

[ref48] Pearson W. B. A Nickel-Gallium Superlattice (Ni_3_Ga). Nature 1954, 173 (4399), 364–364. 10.1038/173364a0.

[ref49] Wang C.; Chen D. P.; Sang X.; Unocic R. R.; Skrabalak S. E. Size-Dependent Disorder–Order Transformation in the Synthesis of Monodisperse Intermetallic PdCu Nanocatalysts. ACS Nano 2016, 10 (6), 6345–6353. 10.1021/acsnano.6b02669. 27214313

[ref50] Lu H. M.; Cao Z. H.; Zhao C. L.; Li P. Y.; Meng X. K. Size-Dependent Ordering and Curie Temperatures of FePt Nanoparticles. J. Appl. Phys. 2008, 103 (12), 123526 10.1063/1.2946724.

[ref51] Alloyeau D.; Ricolleau C.; Mottet C.; Oikawa T.; Langlois C.; Le Bouar Y.; Braidy N.; Loiseau A. Size and Shape Effects on the Order–Disorder Phase Transition in CoPt Nanoparticles. Nat. Mater. 2009, 8 (12), 940–946. 10.1038/nmat2574. 19915553

[ref52] Li C.; Chen Y.; Zhang S.; Zhou J.; Wang F.; He S.; Wei M.; Evans D. G.; Duan X. Nickel–Gallium Intermetallic Nanocrystal Catalysts in the Semihydrogenation of Phenylacetylene. ChemCatChem. 2014, 6 (3), 824–831. 10.1002/cctc.201300813.

[ref53] Chang Y. K.; Lin K. P.; Pong W. F.; Tsai M.-H.; Hseih H. H.; Pieh J. Y.; Tseng P. K.; Lee J. F.; Hsu L. S. Charge Transfer and Hybridization Effects in Ni_3_Al and Ni_3_Ga Studies by x-Ray-Absorption Spectroscopy and Theoretical Calculations. J. Appl. Phys. 2000, 87 (3), 1312–1317. 10.1063/1.372015.

[ref54] Li L.; Chalmers J. A.; Bare S. R.; Scott S. L.; Vila F. D. Rigorous Oxidation State Assignments for Supported Ga-Containing Catalysts Using Theory-Informed X-Ray Absorption Spectroscopy Signatures from Well-Defined Ga(I) and Ga(III) Compounds. ACS Catal. 2023, 13 (10), 6549–6561. 10.1021/acscatal.3c01021.

[ref55] Getsoian A. B.; Das U.; Camacho-Bunquin J.; Zhang G.; Gallagher J. R.; Hu B.; Cheah S.; Schaidle J. A.; Ruddy D. A.; Hensley J. E.; Krause T. R.; Curtiss L. A.; Miller J. T.; Hock A. S. Organometallic Model Complexes Elucidate the Active Gallium Species in Alkane Dehydrogenation Catalysts Based on Ligand Effects in Ga K-Edge XANES. Catal. Sci. Technol. 2016, 6 (16), 6339–6353. 10.1039/C6CY00698A.

[ref56] Schreiber M. W.; Plaisance C. P.; Baumgärtl M.; Reuter K.; Jentys A.; Bermejo-Deval R.; Lercher J. A. Lewis–Bro̷nsted Acid Pairs in Ga/H-ZSM-5 To Catalyze Dehydrogenation of Light Alkanes. J. Am. Chem. Soc. 2018, 140 (14), 4849–4859. 10.1021/jacs.7b12901. 29488757

[ref57] Yuan Y.; Lee J. S.; Lobo R. F. Ga^+^-Chabazite Zeolite: A Highly Selective Catalyst for Nonoxidative Propane Dehydrogenation. J. Am. Chem. Soc. 2022, 144 (33), 15079–15092. 10.1021/jacs.2c03941. 35793461

[ref58] Zhang Z.; Shen C.; Sun K.; Jia X.; Ye J.; Liu C. Advances in Studies of the Structural Effects of Supported Ni Catalysts for CO_2_ Hydrogenation: From Nanoparticle to Single Atom Catalyst. J. Mater. Chem. A 2022, 10 (11), 5792–5812. 10.1039/D1TA09914K.

[ref59] Docherty S. R.; Copéret C. Deciphering Metal–Oxide and Metal–Metal Interplay via Surface Organometallic Chemistry: A Case Study with CO_2_ Hydrogenation to Methanol. J. Am. Chem. Soc. 2021, 143 (18), 6767–6780. 10.1021/jacs.1c02555. 33942617

[ref60] Müller A.; Comas-Vives A.; Copéret C. Ga and Zn Increase the Oxygen Affinity of Cu-Based Catalysts for the CO_x_ Hydrogenation According to Ab Initio Atomistic Thermodynamics. Chem. Sci. 2022, 13 (45), 13442–13458. 10.1039/D2SC03107H. 36507169 PMC9685501

[ref61] Docherty S. R.; Safonova O. V.; Copéret C. Surface Redox Dynamics in Gold–Zinc CO_2_ Hydrogenation Catalysts. J. Am. Chem. Soc. 2023, 145 (25), 13526–13530. 10.1021/jacs.3c03522. 37318330

[ref62] Fielicke A.; Gruene P.; Meijer G.; Rayner D. M. The Adsorption of CO on Transition Metal Clusters: A Case Study of Cluster Surface Chemistry. Surf. Sci. 2009, 603 (10), 1427–1433. 10.1016/j.susc.2008.09.064.

[ref63] Collins S. E.; Briand L. E.; Gambaro L. A.; Baltanás M. A.; Bonivardi A. L. Adsorption and Decomposition of Methanol on Gallium Oxide Polymorphs. J. Phys. Chem. C 2008, 112 (38), 14988–15000. 10.1021/jp801252d.

[ref64] Fehr S. M.; Krossing I. Spectroscopic Signatures of Pressurized Carbon Dioxide in Diffuse Reflectance Infrared Spectroscopy of Heterogeneous Catalysts. ChemCatChem. 2020, 12 (9), 2622–2629. 10.1002/cctc.201902038.

[ref65] Mihaylov M.; Hadjiivanov K.; Knözinger H. Formation of Ni(CO)_4_ during the Interaction between CO and Silica-Supported Nickel Catalyst: An FTIR Spectroscopic Study. Catal. Lett. 2001, 76 (1), 59–63. 10.1023/A:1016786023456.

[ref66] Blyholder G.; Wyatt W. V. Infrared Spectra and Structures of Some C_x_H_y_O Compounds Adsorbed on Silica-Supported Iron, Cobalt, and Nickel. J. Phys. Chem. 1966, 70 (6), 1745 10.1021/j100878a010.

[ref67] Cherevotan A.; Raj J.; Dheer L.; Roy S.; Sarkar S.; Das R.; Vinod C. P.; Xu S.; Wells P.; Waghmare U. V.; Peter S. C. Operando Generated Ordered Heterogeneous Catalyst for the Selective Conversion of CO_2_ to Methanol. ACS Energy Lett. 2021, 6 (2), 509–516. 10.1021/acsenergylett.0c02614.

[ref68] Monti D. M.; Cant N. W.; Trimm D. L.; Wainwright M. S. Hydrogenolysis of Methyl Formate over Copper on Silica: I. Study of Surface Species by in Situ Infrared Spectroscopy. J. Catal. 1986, 100 (1), 17–27. 10.1016/0021-9517(86)90067-9.

[ref69] Millar G. J.; Rochester C. H.; Waugh K. C. Evidence for the Adsorption of Molecules at Special Sites Located at Copper/Zinc Oxide Interfaces. Part 2.—A Fourier-Transform Infrared Spectroscopy Study of Methanol Adsorption on Reduced and Oxidised Cu/ZnO/SiO_2_ Catalysts. J. Chem. Soc., Faraday Trans. 1992, 88 (15), 2257–2261. 10.1039/FT9928802257.

[ref70] Flego C.; Carati A.; Perego C. Methanol Interaction with Mesoporous Silica–Aluminas. Microporous Mesoporous Mater. 2001, 44–45, 733–744. 10.1016/S1387-1811(01)00255-4.

[ref71] Phongprueksathat N.; Ting K. W.; Mine S.; Jing Y.; Toyoshima R.; Kondoh H.; Shimizu K.; Toyao T.; Urakawa A. Bifunctionality of Re Supported on TiO_2_ in Driving Methanol Formation in Low-Temperature CO_2_ Hydrogenation. ACS Catal. 2023, 13 (16), 10734–10750. 10.1021/acscatal.3c01599. 37614518 PMC10442859

[ref72] Meunier F. C.; Dansette I.; Paredes-Nunez A.; Schuurman Y. Cu-Bound Formates Are Main Reaction Intermediates during CO_2_ Hydrogenation to Methanol over Cu/ZrO_2_. Angew. Chem.-Int. Ed. 2023, 62 (29), e202303939 10.1002/anie.202303939. 37212538

[ref73] Collins S. E.; Baltanás M. A.; Bonivardi A. L. An Infrared Study of the Intermediates of Methanol Synthesis from Carbon Dioxide over Pd/β-Ga_2_O_3_. J. Catal. 2004, 226 (2), 410–421. 10.1016/j.jcat.2004.06.012.

[ref74] Efimov A. M.; Pogareva V. G. IR Absorption Spectra of Vitreous Silica and Silicate Glasses: The Nature of Bands in the 1300 to 5000 cm^–1^ Region. Chem. Geol. 2006, 229 (1), 198–217. 10.1016/j.chemgeo.2006.01.022.

[ref75] Larmier K.; Liao W.-C.; Tada S.; Lam E.; Verel R.; Bansode A.; Urakawa A.; Comas-Vives A.; Coperet C. CO_2_-to-Methanol Hydrogenation on Zirconia-Supported Copper Nanoparticles: Reaction Intermediates and the Role of the Metal-Support Interface. Angew. Chem.-Int. Ed. 2017, 56 (9), 2318–2323. 10.1002/anie.201610166. 28111850

[ref76] Göttker-Schnetmann I.; Mecking S. A Practical Synthesis of [(Tmeda)Ni(CH_3_)_2_], Isotopically Labeled [(Tmeda)Ni(^13^CH_3_)_2_], and Neutral Chelated-Nickel Methyl Complexes. Organometallics 2020, 39 (18), 3433–3440. 10.1021/acs.organomet.0c00500.

[ref77] Juhás P.; Davis T.; Farrow C. L.; Billinge S. J. L. PDFgetX3: A Rapid and Highly Automatable Program for Processing Powder Diffraction Data into Total Scattering Pair Distribution Functions. J. Appl. Crystallogr. 2013, 46 (2), 560–566. 10.1107/S0021889813005190.

[ref78] Farrow C. L.; Juhas P.; Liu J. W.; Bryndin D.; Božin E. S.; Bloch J.; Proffen T.; Billinge S. J. L. PDFfit2 and PDFgui: Computer Programs for Studying Nanostructure in Crystals. J. Phys.: Condens. Matter 2007, 19 (33), 335219 10.1088/0953-8984/19/33/335219. 21694142

[ref79] CRediT (Contribuor Roles Taxonomy). https://credit.niso.org/ (accessed February 20, 2023).

