# Supplementary material for: Structure and Role of a Ga-Promoter in Ni-Based Catalysts for the Selective Hydrogenation of CO2 to Methanol
Source: JACS Au. 2024 Jan 3;4(1):237–52. doi: 10.1021/jacsau.3c00677 (PMC10806875; doi:10.1021/jacsau.3c00677)
Supplement: Supplementary file 1 — au3c00677_si_001.pdf [file au3c00677_si_001.pdf]

Supporting Information

Structure and Role of a Ga-Promoter in Ni-Based Catalysts for the Selective Hydrogenation of CO_2 to Methanol

Nora K. Zimmerli[a‡], Lukas Rochlitz[b‡], Stefano Checchia[c], Christoph R. Müller[a*], Christophe Copéret[b*], and Paula M. Abdala[a*]

a Department of Mechanical and Process Engineering, ETH Zürich, Leonhardstrasse 21, CH 8092 Zürich, Switzerland

b Department of Chemistry and Applied Biosciences, ETH Zürich, Vladimir-Prelog-Weg 2, CH 8093 Zürich, Switzerland

c ESRF – The European Synchrotron, 71 Avenue des Martyrs, 38000 Grenoble, France

‡ These authors contributed equally to this work.

Table of contents

Table of contents 1

1 TEM & -HAADF-STEM/EDX 2

1.1. Ni_{100}/SiO_2 2

1.2. Ga_{100}/SiO_2 2

1.3. $Ni_{75}Ga_{25}/SiO_2$ 3

1.4. $Ni_{70}Ga_{30}/SiO_2$ 5

1.5. $Ni_{65}Ga_{35}/SiO_2$ 7

2 CO_2 hydrogenation tests 9

3 XAS and X-ray total scattering experiments 14

3.1. Collection of X-ray total scattering data and pair distribution function (PDF) analysis 14

3.2. XAS measurements 15

3.2.1. Collection of XAS data 15

3.2.2. XAS data analysis 15

4 Diffuse Reflectance Infrared Fourier Transform Spectroscopy 40

References 43

1 TEM & -HAADF-STEM/EDX

1.1. Ni_{100}/SiO_2

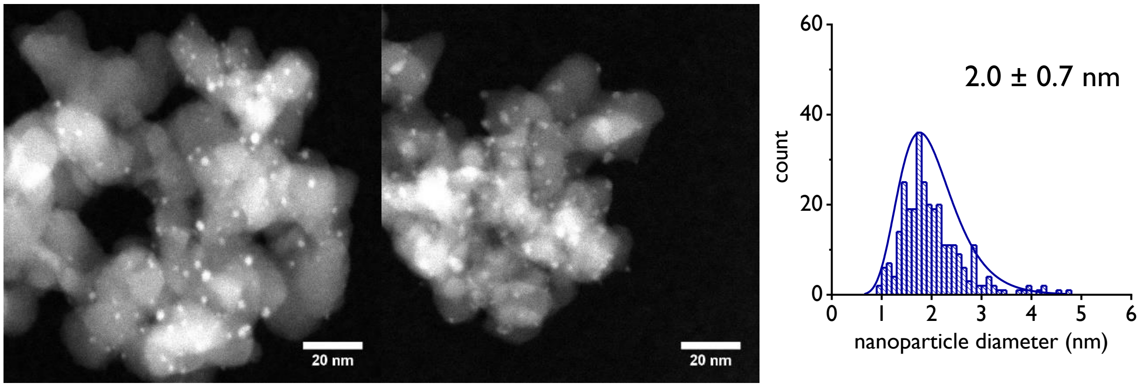

Figure S1. Representative HAADF-STEM images of as-prepared **Ni_{100}/SiO_2** and corresponding particle size distribution.

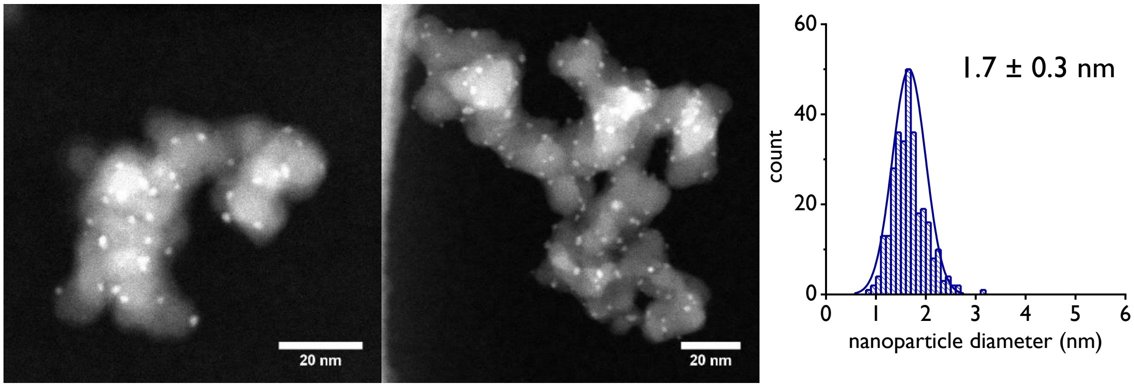

Figure S2. Representative HAADF-STEM images of spent **Ni_{100}/SiO_2** and corresponding particle size distribution after 4.5 h on-stream under CO_2 hydrogenation conditions (25 bar CO_2:N_2:H_2 = 1:1:3, GHSV = 60 L h^{-1} $gcat^{-1}$, 230°C).

1.2. Ga_{100}/SiO_2

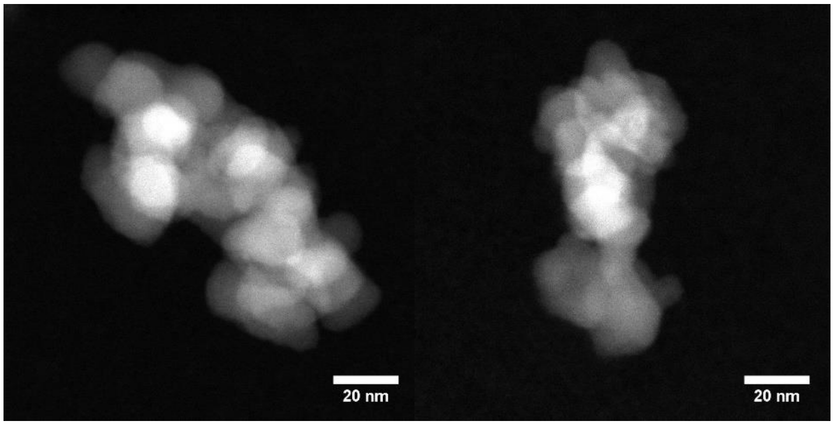

Figure S3. Representative HAADF-STEM images of as-prepared **Ga_{100}/SiO_2** before (left) and after 4 h on-stream under CO_2 hydrogenation conditions (right, 25 bar CO_2:N_2:H_2 = 1:1:3, GHSV = 60 L h^{-1} $gcat^{-1}$, 230°C).

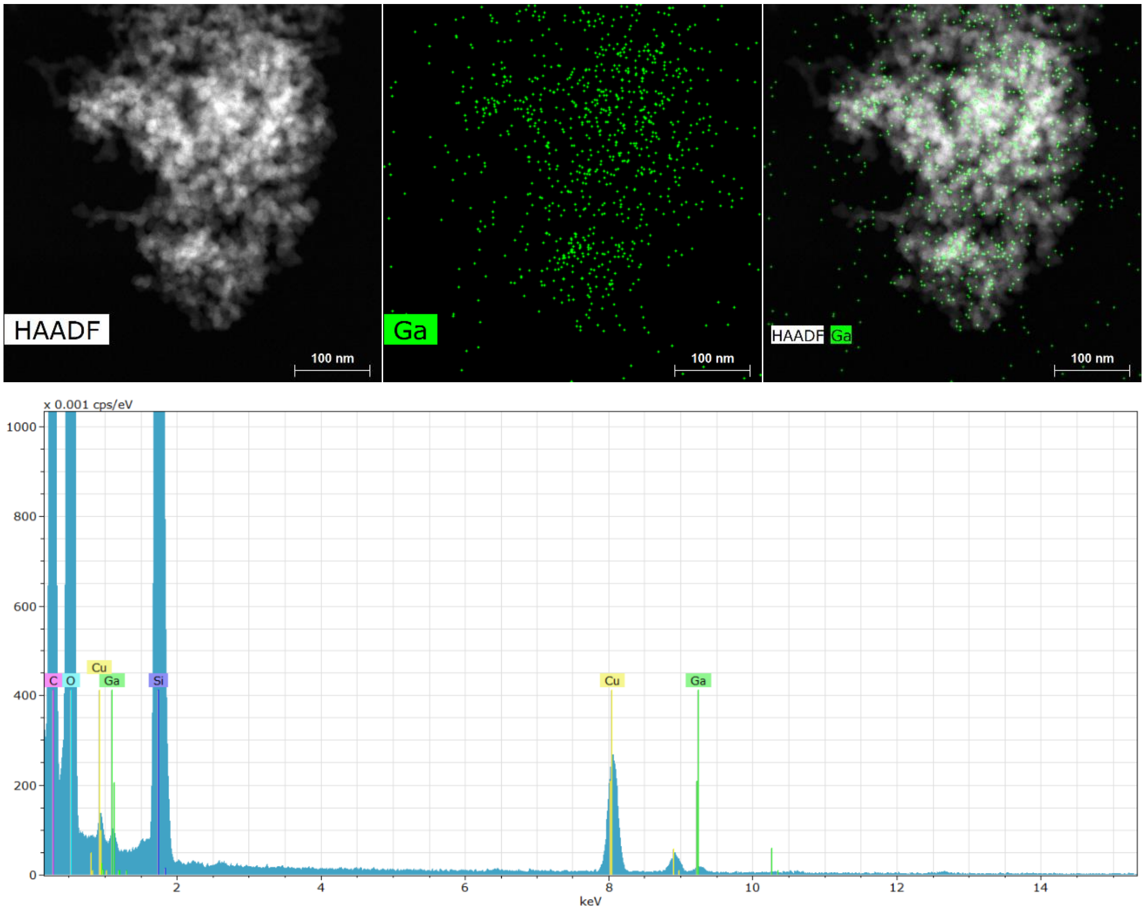

Figure S4. Representative HAADF-STEM/EDX image of as-prepared **Ga_{100}/SiO_2** and the corresponding EDX spectrum.

1.3. $Ni_{75}Ga_{25}/SiO_2$

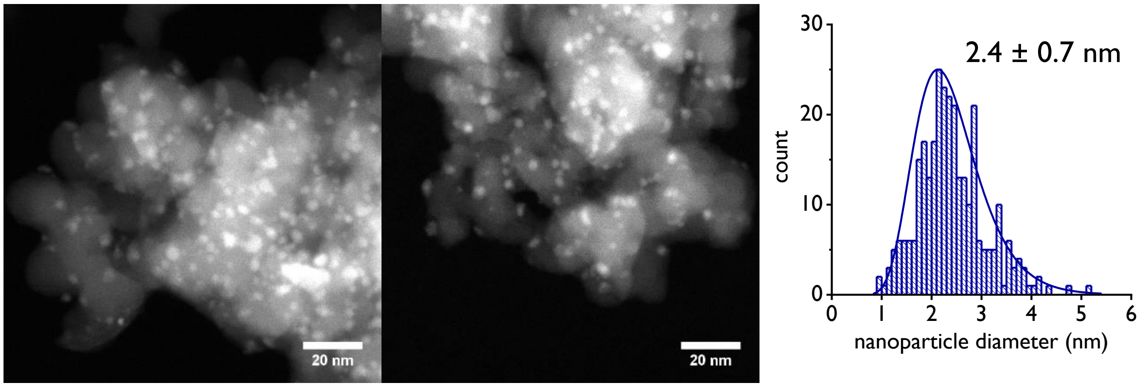

Figure S5. Representative HAADF-STEM images of as-prepared **$Ni_{75}Ga_{25}/SiO_2$** and the corresponding particle size distribution.

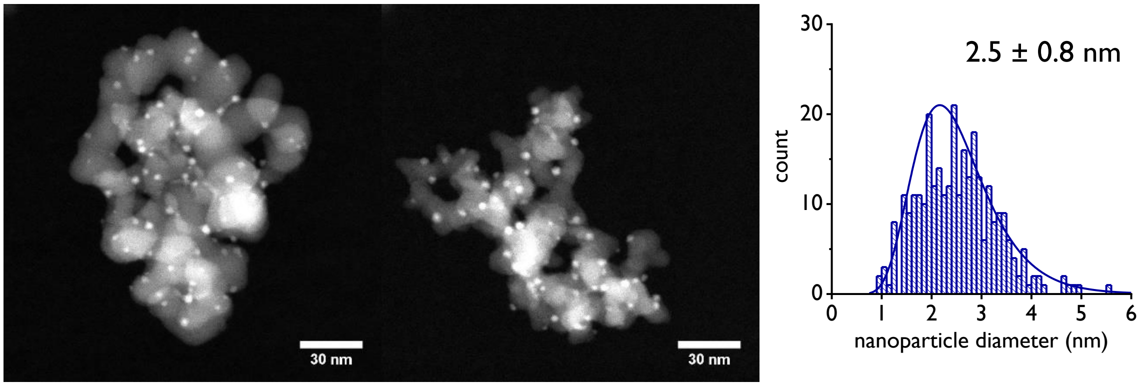

Figure S6. Representative HAADF-STEM images of spent **$Ni_{75}Ga_{25}/SiO_2$** and the corresponding particle size distribution after 4 h on-stream under CO_2 hydrogenation conditions (25 bar CO_2:N_2:H_2 = 1:1:3, GHSV = 60 L h^{-1} $gcat^{-1}$, 230°C).

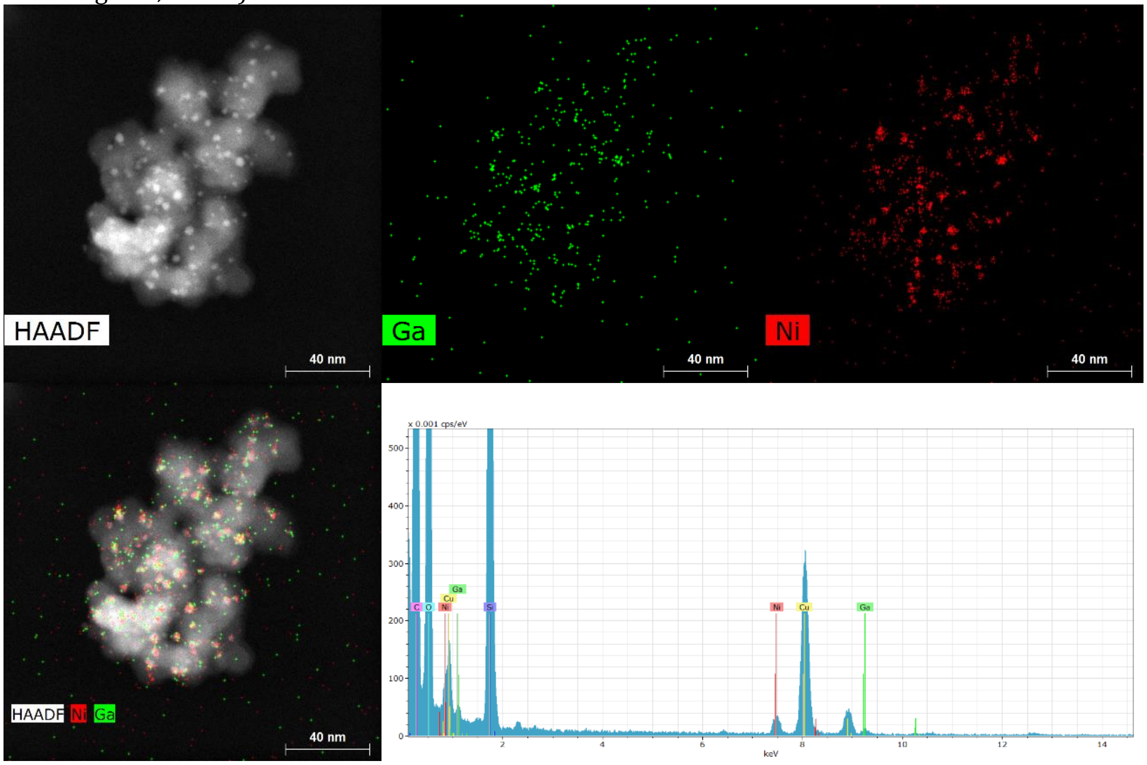

Figure S7. Representative HAADF-STEM/EDX image of as-prepared **$Ni_{75}Ga_{25}/SiO_2$** and the corresponding EDX spectrum.

1.4. $Ni_{70}Ga_{30}/SiO_2$

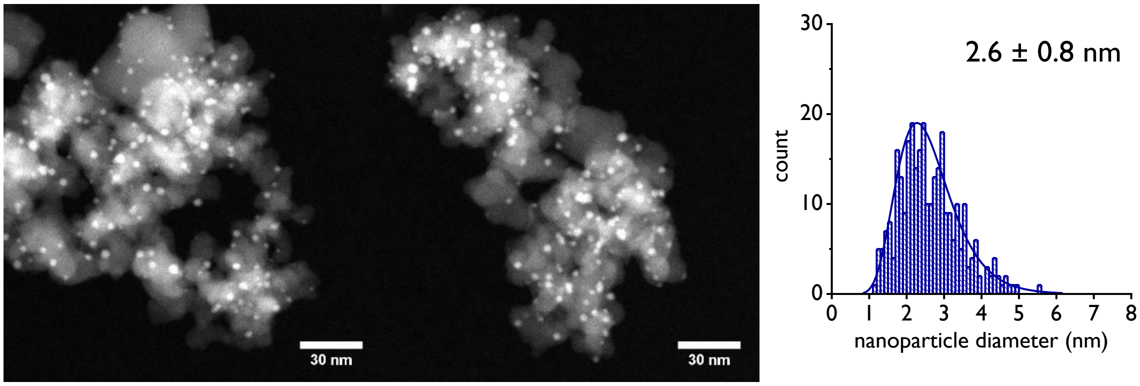

Figure S8. Representative HAADF-STEM images of as-prepared **$Ni_{70}Ga_{30}/SiO_2$** and the corresponding particle size distribution.

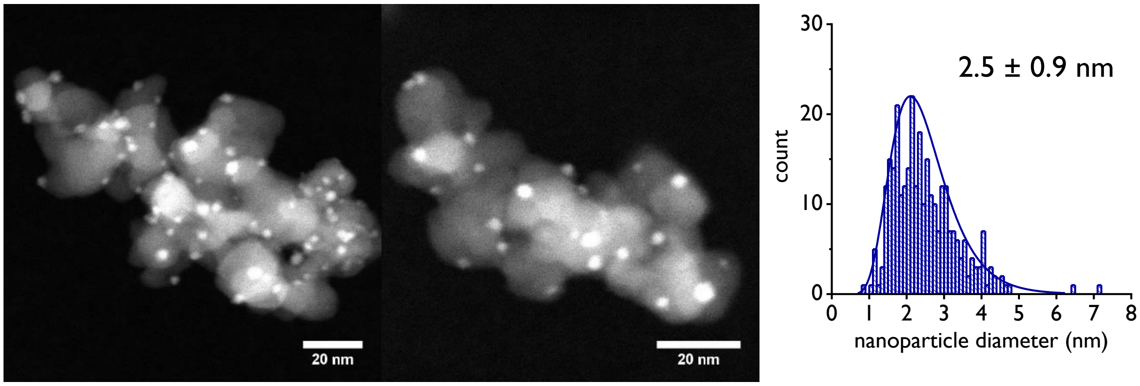

Figure S9. Representative HAADF-STEM images of spent **$Ni_{70}Ga_{30}/SiO_2$** and the corresponding particle size distribution after 3.5 h on-stream under CO_2 hydrogenation conditions (25 bar CO_2:N_2:H_2 = 1:1:3, GHSV = 60 L h^{-1} $gcat^{-1}$, 230°C).

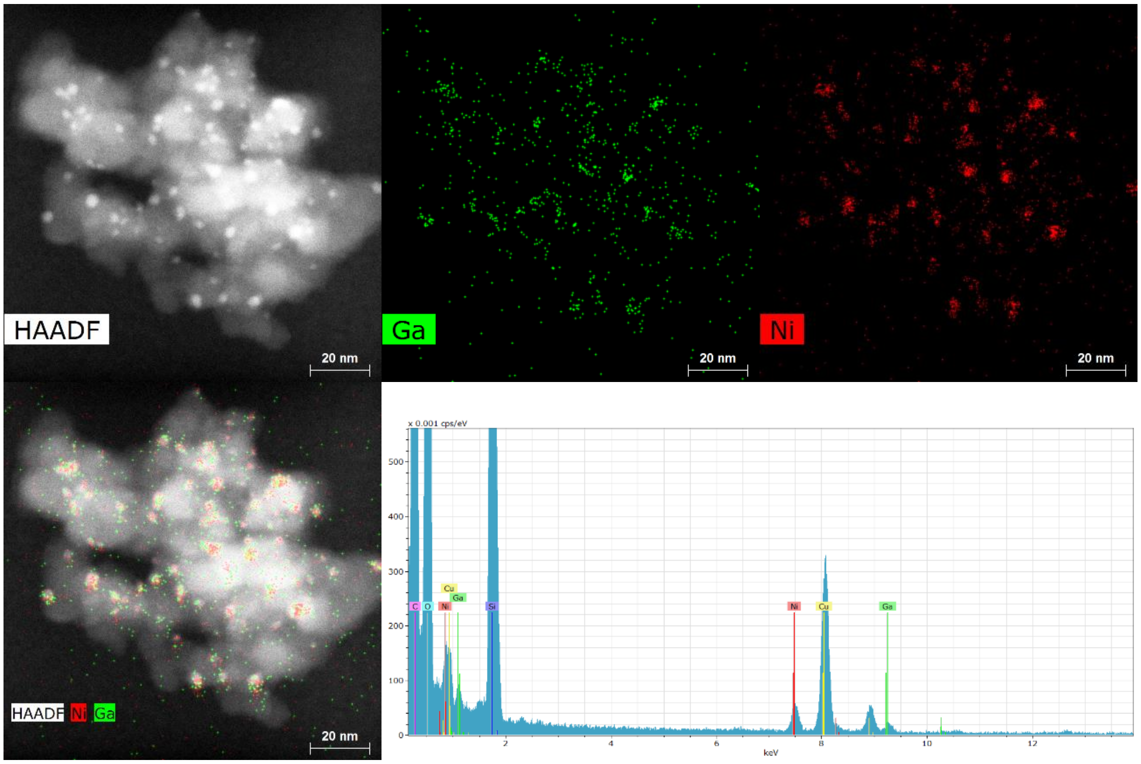

Figure S10. Representative HAADF-STEM/EDX image of as-prepared **$Ni_{70}Ga_{30}/SiO_2$** and the corresponding EDX spectrum.

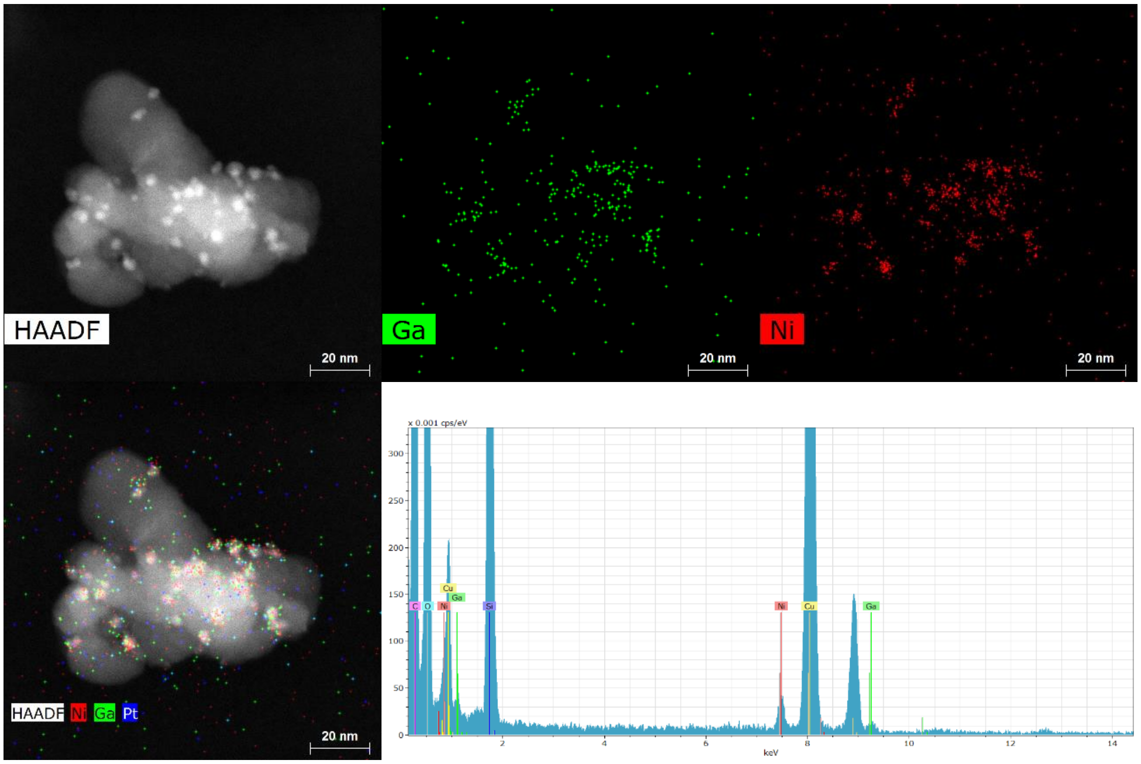

Figure S11. Representative HAADF-STEM/EDX image of spent **$Ni_{70}Ga_{30}/SiO_2$** and the corresponding EDX spectrum.

1.5. $Ni_{65}Ga_{35}/SiO_2$

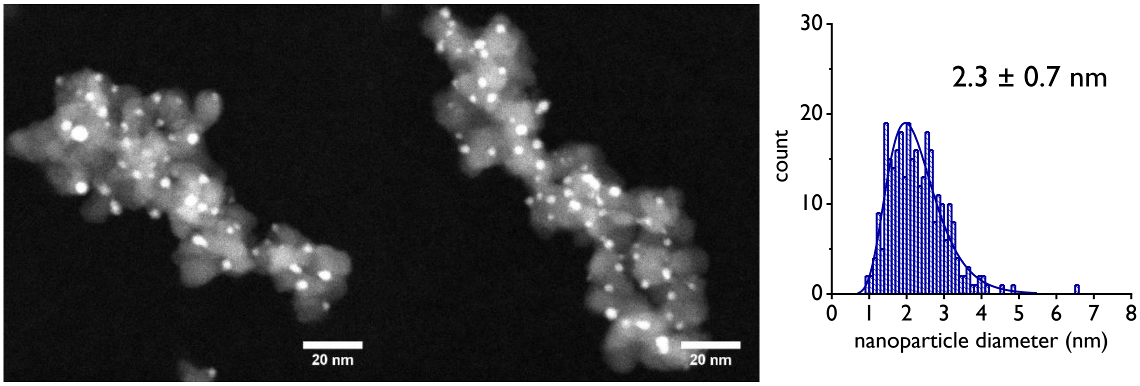

Figure S12. Representative HAADF-STEM images of as-prepared **$Ni_{65}Ga_{35}/SiO_2$** and the corresponding particle size distribution.

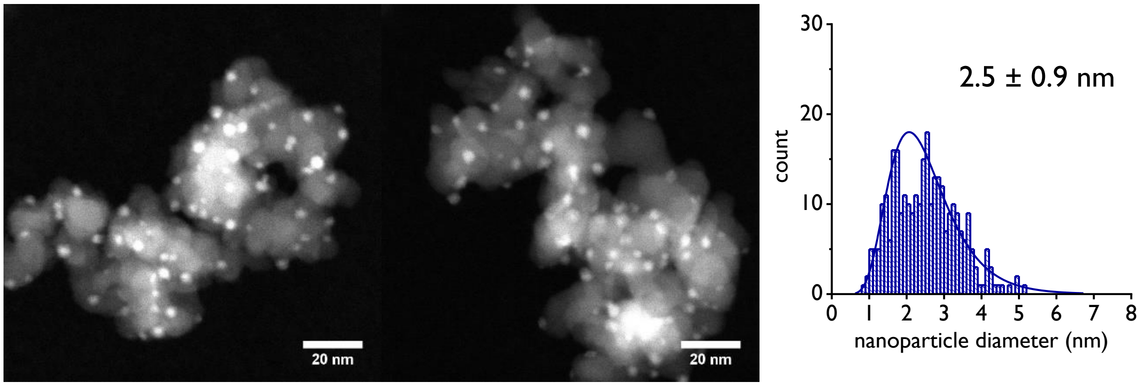

Figure S13. Representative HAADF-STEM images of spent **$Ni_{65}Ga_{35}/SiO_2$** and the corresponding particle size distribution after 4 h on stream under CO_2 hydrogenation conditions (25 bar CO_2:N_2:H_2 = 1:1:3, GHSV = 60 L h^{-1} $gcat^{-1}$, 230°C).

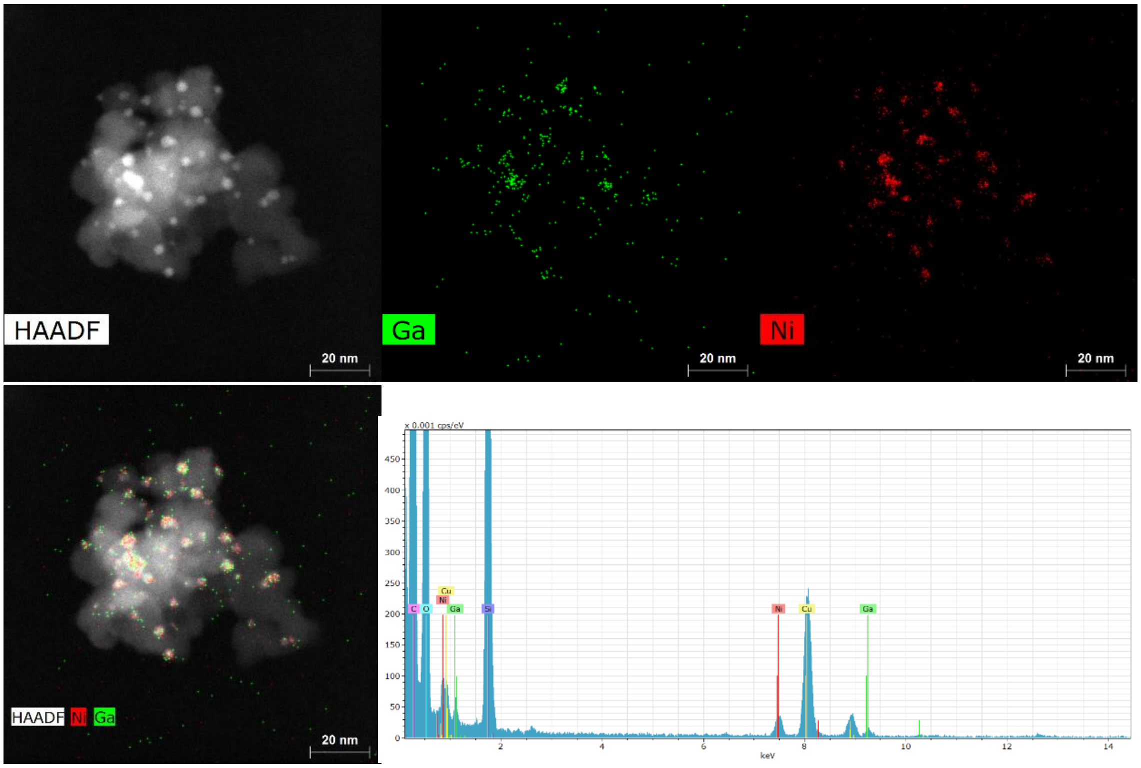

Figure S14. Representative HAADF-STEM/EDX image of as-prepared **$Ni_{65}Ga_{35}/SiO_2$** and the corresponding EDX spectrum.

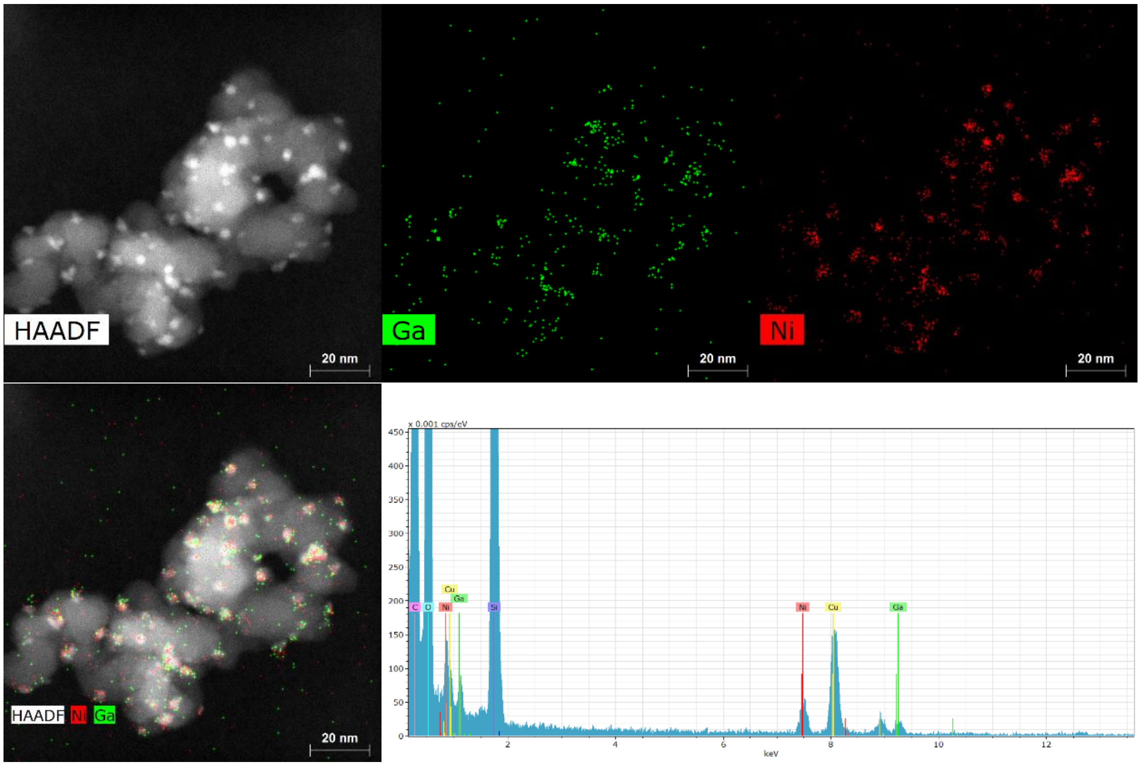

Figure S15. Representative HAADF-STEM/EDX image of spent **$Ni_{65}Ga_{35}/SiO_2$** and the corresponding EDX spectrum.

2 CO_2 hydrogenation tests

In addition to the $Ni_xGa_{(100-x)}/SiO_2$, we also tested 100 mg of commercial $Cu/ZnO/Al_2O_3$ methanol synthesis catalyst (Alfa Aesar, #45776-36). For this test, the catalyst was loaded into the reactor under ambient air conditions and the in-situ catalyst activation was done by heating to 300°C (10°/min) in 1 bar 5% H_2/N_2 (50 ml/min) and holding at 300°C for 1h. All catalytic performance results from this work, as well as selected results from literature, can be found in Tables S1 and S2.

The catalytic performance parameters were calculated as follows:

Total off-gas flow rate (mol min^{-1}):

$$F_{tot,out} = \frac{F_{tot,in} \times C_{N2,in}}{C_{N2,out}}$$

Mass formation rate of product i per total catalyst mass ($g_i\ g_{cat}^{-1}\ h^{-1}$):

$$r_{i,gcat} = \frac{C_i \times F_{tot,out} \times MW_i \times 60\left(\frac{min}{h}\right)}{m_{cat}}$$

Molar formation rate of product i per mol of Ni in the catalyst ($mmol_i\ mol_{Ni}^{-1}\ s^{-1}$):

$$r_{i,Ni} = \frac{C_i \times F_{tot,out} \times 10^3\left(\frac{mmol}{mol}\right) \times MW_{Ni}}{m_{cat} \times w_{Ni} \times 60\left(\frac{s}{min}\right)}$$

Molar formation rate of product i per mol of (Ni+Ga) in the catalyst ($mmol_i\ mol_{(Ni+Ga)}^{-1}\ s^{-1}$):

$$r_{i,Ni} = \frac{C_i \times F_{tot,out} \times 10^3\left(\frac{mmol}{mol}\right)}{m_{cat} \times \left(\frac{w_{Ni}}{MW_{Ni}} + \frac{w_{Ga}}{MW_{Ga}}\right) \times 60\left(\frac{s}{min}\right)}$$

Selectivity of product i (%):

$$S_i = \frac{C_i}{C_{CH3OH} + C_{CO} + C_{CH4}} \times 100\ \%$$

CO_2 conversion (%):

$$X_{CO2} = \frac{F_{tot,out} \times (C_{CH3OH} + C_{CO} + C_{CH4})}{F_{tot,in} \times C_{CO2,in}} \times 100\ \%$$

where $\boldsymbol{F_{tot,in}}$ **(mol min^{-1})** is the total gas flow rate at the inlet of the reactor, $\boldsymbol{C_{N2,in}}$ **(-)** is the concentration of N_2 in the inlet gas, $\boldsymbol{C_{N2,out}}$ **(-)** is the concentration of N_2 in the off-gas, $\boldsymbol{C_i}$ **(-)** is the concentration of product i ∈{CH_3OH, CO, CH_4} in the off-gas, **MW_i (g mol^{-1})** is the molecular weight of species i, m_{cat} (g) is the catalyst mass, $\boldsymbol{w_{Ni}}$ (-) and $\boldsymbol{w_{Ga}}$ (-) are the weight fractions of Ni and Ga in the catalyst as measured by ICP, and $\boldsymbol{C_{CO2,in}}$ **(-)** is the concentration of CO_2 in the inlet gas.

Supplementary plots

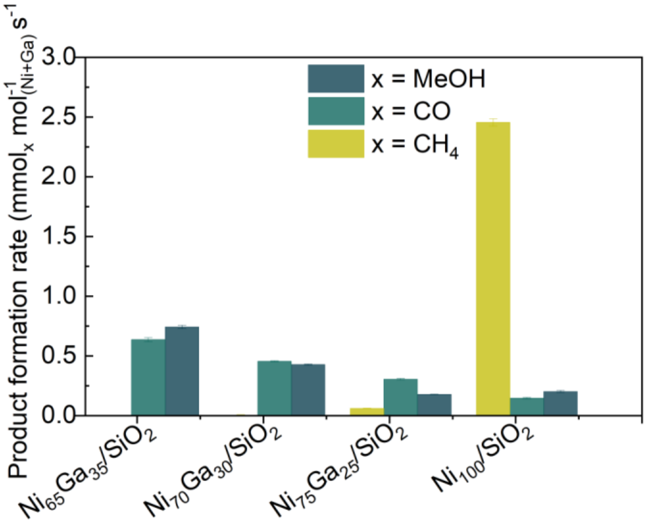

Figure S16. Molar product formation rates of the $Ni_xGa_{(100-x)}/SiO_2$ normalized by the catalysts' total metal contents (i.e. Ni+Ga moles) determined by elemental analysis (mmol$_x$ mol$_{(Ni+Ga)}^{-1}$ s^{-1}).

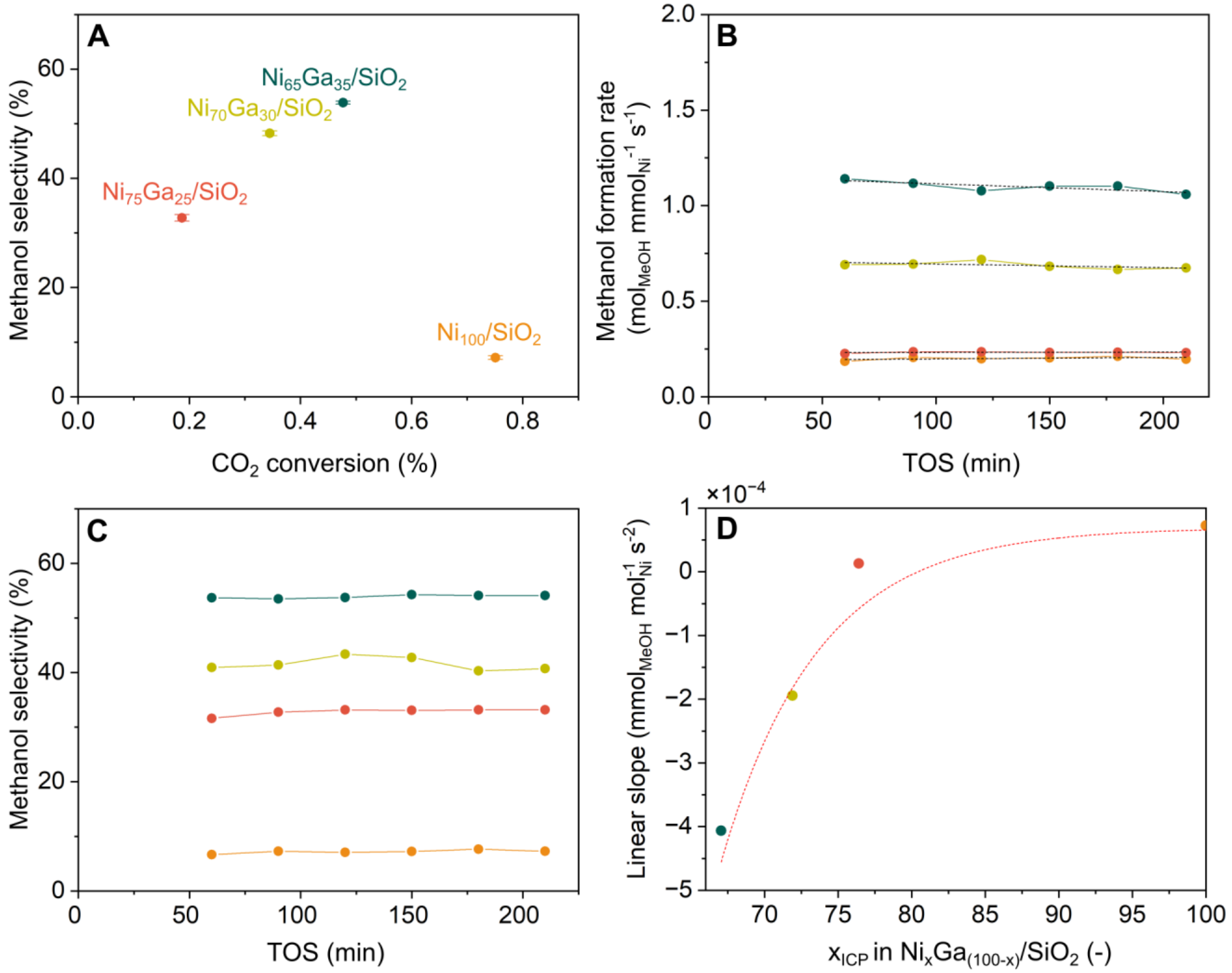

Figure S17. (A) Methanol selectivity as a function of CO_2 conversion. (B) Methanol formation rate as a function of time-on-stream under CO_2 hydrogenation conditions with linear fits (black dotted lines) indicating the deactivation trends. (C) Methanol selectivity as a function of TOS. (D) Slope of the linear curves fitted to the methanol formation rates in (B) as a function of x_{ICP} in $Ni_xGa_{(100-x)}/SiO_2$. Conditions: 25 bar CO_2:N_2:H_2 = 1:1:3, GHSV = 60 L h^{-1} $gcat^{-1}$, 230°C.

Table S1. Composition (as determined by ICP-OES), mean TEM particle diameter of the as-prepared catalysts and methanol formation rates and selectivities determined in a fixed bed flow reactor at 25 bar CO_2:H_2:N_2 = 1:3:1, 230°C, GHSV = 60 L g_{cat}^{-1} h^{-1}. [(a)]w_{Ni} + w_{Ga} + w_{SiO2} = 100 wt% and n_i denote the number of moles of species i. [(b)]"± x" indicates the standard deviation from the mean. [(c)]Mean values over the first 180 minutes TOS (= 5 GC samples). The value in parentheses denotes the uncertainty of the least significant digit of the mean.

Material	Elemental analysis results[(a)]			Mean particle diameter (nm)[(b)]	**Methanol formation rate[(c)] ($mmol_{MeOH}$ $mol_{Ni}^{-1}s^{-1}$ /10^{-2}*g_{MeOH} $g_{cat}^{-1}h^{-1}$)**	Selectivity (%)		
	Ni (wt%)	Ga (wt%)	n_{Ni}:n_{Ga} (-)			Methanol	CO	CH_4
Ni_{100}/SiO_2	2.12	0	100:0	2.0 ± 0.7	0.201(9)/0.84(4)	7.2(3)	5.2(2)	87.6(5)
$Ni_{75}Ga_{25}/SiO_2$	2.08	0.76	76:24	2.4 ± 0.7	0.232(3)/0.95(1)	32.7(6)	56.2(6)	11.1(3)
$Ni_{75}Ga_{25}/SiO_2$-XAS	1.84	0.55	80:20	-	0.193(6)/0.70(2)	14.3(1)	42.7(7)	43.0(6)
$Ni_{70}Ga_{30}/SiO_2$	2.21	1.03	72:28	2.6 ± 0.8	0.594(7)/2.58(3)	48.2(4)	51.4(6)	0.3(4)
$Ni_{65}Ga_{35}/SiO_2$	1.83	1.07	67.0:33.0	-	1.11(2)/3.98(7)	53.9(3)	46.1(3)	0
$Ni_{65}Ga_{35}/SiO_2$-XAS	2.17	1.24	67.5:32.5	2.3 ± 0.7	0.94(2)/4.01(9)	52.5(3)	47.5(3)	0
Ga_{100}/SiO_2	0.88	0	0:100	-	0	-	-	-

Table S2. Selected Ni, Cu, and Pd based catalysts used for the CO_2 hydrogenation to methanol. Comparison of the reported values for the methanol formation rates, and corresponding methanol selectivities.[1] [(a)]In case that no elemental analysis results were reported, the reported nominal metal loadings and metal ratios were used to calculate the metal normalized methanol formation rates. [(b)]In this study, only the CO-free selectivity was reported.

Material	Feed gas composition	P (bar)	T (°C)	GHSV (ml min^{-1} $gcat^{-1}$)	Methanol selectivity (%)	Methanol formation rate (g_{MeOH} g_{cat}^{-1} h^{-1})	Methanol formation rate[(a)] (mmol MeOH $mol_M^{-1}s^{-1}$)	Methanol formation rate[(a)] (mmol MeOH $mol_{M1}^{-1}s^{-1}$)	Reference
$Ni_{65}Ga_{35}/SiO_2$	1:3:1 CO_2:H_2:N_2	25	230	1000	54	0.04	0.74 M = Ni+Ga	1.11 M1 = Ni	This work
Ni_5Ga_3/SiO_2	1:3 CO_2:H_2	1	215	211	99[(b)]	0.02	0.06 M = Ni+Ga	0.10 M1 = Ni	1
Ni_5Ga_3/SiO_2 "ex-situ(700 °C)/ in-situ(400 °C)"	1:3 CO_2:H_2	5	250	200	60	0.03	0.11 M = Ni+Ga	0.18 M1 = Ni	2
PdGa@SiO_2	1:3:1 CO_2:H_2:Ar	25	230	500	79	0.07	1.82 M = Pd+Ga	6.09 M1 = Pd	3
Cu-Ga/SiO_2	1:3:1 CO_2:H_2:N_2	25	230	400	90	0.05	0.50 M = Cu+Ga	0.69 M1 = Cu	4
Cu-Zn/SiO_2	1:3:1 CO_2:H_2:N_2	25	230	400	80	0.07	0.64 M = Cu+Zn	0.88 M1 = Cu	5
Cu-SiO_{2P}-225	23:73:4 CO_2:H_2:N_2	30	250	200	44.3	0.13	1.33 M = Cu	1.33 M1 = Cu	6
Cu/ZnO/Al_2O_3	1:3:1 CO_2:H_2:N_2	25	230	1000	48	0.31	0.24 M = Cu+Zn	0.34 M1 = Cu	This work

[1] Note that some of these values were read out from graphs which may have resulted in some inaccuracy.

3 XAS and X-ray total scattering experiments

In situ/ operando XAS and X-ray total scattering experiments were performed at the beamlines BM31 and ID15A of the European Synchrotron Radiation Facility, respectively. The setup in both cases consisted of a custom-made capillary cell reactor which was connected to a manifold of mass flow controllers (Bronkhorst EL-FLOW series, max. 30 ml/min, P_{max} = 35 bar) and a backpressure regulator (Bronkhorst, EL-PRESS series, P_{max} = 35 bar). The catalyst bed consisted of ca. 2-3 mg of catalyst placed between two quartz wool plugs inside a quartz capillary (Hilgenberg, 1 mm OD, 0.02 mm wall thickness). The capillary was heated via a hot air blower from below. The temperature was carefully calibrated between 50 – 800 °C prior to the experiments by placing a thermocouple in a capillary filled with quartz wool. The off-gas was analyzed via a compact gas chromatograph (Global Analyzer Solutions, Compact $GC^{4.0}$) equipped with TCD and FID detectors and a sampling rate of ca. 1/7 min^{-1}. The limits of detection of the GC are approximately < 10 ppm for methanol and CH_4 and < 500 ppm for CO. A typical in situ/operando experiment consisted of in situ activation, pressurizing and CO_2 hydrogenation steps which are detailed in Figure S18 C and D for XAS and total scattering experiments, respectively. The difference between the two sets of experiments is that in the XAS experiments, the catalyst was cooled down to 50°C after in situ activation to collect EXAFS data, whereas in the total scattering experiments the catalyst was only cooled down to 230°C. XAS data were measured in transmission mode, via ion chamber detectors placed before and after the sample (Figure S18 A) and X-ray total scattering measurements were collected via an area detector (Pilatus3 CdTe 2M) placed behind the sample (Figure S18 B).

3.1. Collection of X-ray total scattering data and pair distribution function (PDF) analysis

X-ray total scattering data of $Ni_{65}Ga_{35}/SiO_2$, $Ni_{70}Ga_{30}/SiO_2$, $Ni_{75}Ga_{25}/SiO_2$, and Ni_{100}/SiO_2 were collected continuously at an incident X-ray energy of 90.0 keV (0.138 Å) up to $Q_{max,instr}$ = 30 $Å^{-1}$ at a rate of 1 measurement/2.62 minutes. For $Ni_{65}Ga_{35}/SiO_2$-XAS, total scattering data was collected at 68.5 keV (0.181 Å) up to $Q_{max,instr}$ = 26 $Å^{-1}$ at a rate of 1 measurement/4.65 minutes. Total scattering data of the pristine silica support was measured under in situ activation and reaction conditions and used as background to calculate the d-PDF data of the $Ni_xGa_{(100-x)}/SiO_2$ (see Figure S22). In addition, total scattering data of the CeO_2 NIST reference materials were obtained to determine the experimental resolution parameters Q_{damp} and Q_{broad}.

To obtain the differential PDF (d-PDF) data from the total scattering patterns of $Ni_xGa_{(100-x)}$, we performed background subtraction, data normalization and Fourier transform using the PDFgetX3 software (v 2.2.1).[7,8] The total scattering data was processed within the range Q_{min} = 1.5 $Å^{-1}$ and Q_{max} = 23 $Å^{-1}$ with r_{poly} = 1.0, which is approximately the r-limit for the maximum frequency in the F(Q) correction polynomial.

Modelling of the d-PDF was performed in PDFGui (v 1.0),[9,10] using a random alloy model structure generated from fcc-Ni (Inorganic Crystal Structure Database, ICSD # 8688) in which x % Ni atoms (x as in $Ni_xGa_{(100-x)}$) were substituted by Ga. The fitted parameters included the scale factor, the cubic lattice parameter, an isotropic atomic displacement factor (same for Ni and Ga), the "*delta2*" atomic motion correlation factor, and the coherent particle (crystallite) size. The Q_{damp} and Q_{broad} parameters were set to the values obtained from the fitting of the CeO_2 reference measurements (i.e. Q_{damp}= 0.0180 $Å^{-1}$ and Q_{broad}= 0.0159 $Å^{-1}$). We note here that we also tested modelling the d-PDF using a α'-Ni_3Ga model structure (*Pm-3m* space group, Ni at Wyckoff position 3c with atomic coordinates 0,0.5,0.5 and Ga at Wyckoff position 1a and coordinates 0,0,0, see ICSD #103856) which resulted in fitted parameters equal (within the error) to the ones obtained with the random alloy. The d-PDF

were fitted between 1.7 – 25 Å (respective fit and lattice parameters are shown in Figures 2 B and 2 D) however, boxcar fittings between 2-7, 5-10, 8-13, and 11-16 Å were also performed to evaluate whether the structures in the different r ranges of the d-PDF are comparable.

The Ni:Ga ratios in the alloys (Tables 1 and S5) were obtained using the refined cell parameters obtained from the fitting of the d-PDF data and the slope of Vegard's law. The validity of Vegard's law (linear correlation between the lattice parameter and the Ni:Ga ratio) was verified for fcc $Ni_yGa_{(100-y)}$ alloys by using reported literature values (Table S4, data collected at room temperature, 25°C). After extracting the slope of Vegard's law, the obtained value was applied to the data collected for Ni_{100}/SiO_2 at 230°C. Note that this approach also accounts for potential differences in the cell parameters between bulk Ni and nanostructure Ni (see Figure S29). The $Ga_{alloyed}$ and GaO_x amounts normalized by the Ni contents of the catalysts reported in Table 1 were obtained via the following equations, considering that all Ni in the catalysts is alloyed and that GaO_x (see XAS analysis) is the difference between the total (determined by ICP) and alloyed Ga contents:

$$Ga_{alloyed}\left(mol_{Gaalloyed}mol_{Ni}^{-1}\right) = \left(\frac{n_{Ga,alloy}}{n_{Ni,alloy}}\right) = \left(\frac{n_{Ga,alloy}}{n_{Ni}}\right)$$

$$GaO_x\left(mol_{GaOx}mol_{Ni}^{-1}\right) = \frac{n_{Ga,ICP}}{n_{Ni}} - Ga_{alloyed}$$

where $\left(\frac{n_{Ga,alloy}}{n_{Ni,alloy}}\right)$ is the Ga:Ni ratio in the alloy obtained from the d-PDF analysis, and $\frac{n_{Ga,ICP}}{n_{Ni}}$ is the ratio between the total amounts of Ga and Ni determined by ICP (where we assume $n_{Ni} = n_{Ni,alloy}$).

3.2. XAS measurements

3.2.1. Collection of XAS data

Ni and Ga K-edge XAS scans were collected consecutively, using XANES and EXAFS macros, covering the energy ranges specified below. The data collected during the in situ activation step (heating up from room temperature to 600°C in 1 bar H_2) were collected using the XANES macro, whereas the EXAFS macro was used for isothermal measurements.

XANES macro:

1. Ni XANES, 8250-8550 eV, ΔE = 0.3 eV, 50 seconds/spectrum
2. Ga XANES, 10300-10600 eV, ΔE = 0.3 eV, 50 seconds/spectrum

EXAFS macro:

1. Ni EXAFS, 8200-8970 eV, ΔE = 0.5 eV, 3 minutes/spectrum
2. Ga EXAFS, 10200-11100 eV, ΔE = 0.5 eV, 3 minutes/spectrum

We collected in situ XAS of $Ni_{65}Ga_{35}/SiO_2$-XAS and $Ni_{75}Ga_{25}/SiO_2$-XAS catalysts, whereas activated Ni_{100}/SiO_2 and Ga_{100}/SiO_2 data were measured ex-situ (in air-tight capillaries containing an N_2 atmosphere). This was achieved by transferring the activated material, without exposure to air inside a glovebox.

3.2.2. XAS data analysis

The energy scale at the Ni and Ga K-edges spectra were calibrated by setting the absorption edge positions (set at the maximum of the first derivative of μ(E)) of the reference samples Ni-foil and Zn foil to the known values of 8333.0 eV and 9659.0 eV respectively.

Linear combination fittings of the normalized XANES (μ(E)) were performed using the Athena/Demeter v 0.9.26 software[11] between -20 and +50 eV around the edge position ("E0"), constraining the LCF weights to values between 0 and 1 and their sum to 1.

EXAFS fittings were performed using the Artemis/Demeter v 0.9.26 software.[11] For $Ni_xGa_{(100-x)}$ /SiO_2, the fitted k values were in the range 3 - 11.5 $Å^{-1}$ (dk = 1 $Å^{-1}$) for Ni K-edge EXAFS and 3 – 10 $Å^{-1}$ (dk = 1 $Å^{-1}$) for Ga K-edge EXAFS. The fitted r-range extended from 1 – 3 Å (dr = 0.5 Å). The amplitude reduction factors (S_0^2) of the Ni and Ga K-edges were determined from the fittings of Ni-foil, α'-Ni_3Ga and β-Ga_2O_3 reference materials as $S_{0,Ni}^2$ = 0.82 and $S_{0,Ga}^2$ = 0.92 (average of the values obtained from α'-Ni_3Ga = 0.93 and β-Ga_2O_3 = 0.91). The reference materials were fitted within the following k and r ranges: Ni-foil: k_{min} = 3 $Å^{-1}$, k_{max} = 13 $Å^{-1}$, dk = 1 $Å^{-1}$, r_{min} = 1 Å, r_{max} = 3 Å, dr = 0.5 Å. α'-Ni_3Ga: k_{min} = 3 $Å^{-1}$, k_{max} = 12.5 $Å^{-1}$, dk = 1 $Å^{-1}$, r_{min} = 1.5 Å, r_{max} = 3 Å, dr = 0.5 Å. β-Ga_2O_3: k_{min} = 3 $Å^{-1}$, k_{max} = 14 $Å^{-1}$, dk = 0.5 $Å^{-1}$, r_{min} = 1 Å, r_{max} = 3.4 Å, dr = 0.5 Å.

In the EXAFS fittings we included the first metal-metal spheres, as well as a Ga-O sphere. One limitation of EXAFS is the difficulty in distinguishing Ga and Ni scattering owing to their similar atomic numbers[12] and similar interatomic distances. However, by fitting simultaneously the Ni and Ga K-edge data of the same catalyst (see below) and applying a set of constraints to the fitting parameters, we aimed to distinguished between the overlapping Ni-Ni, Ni-Ga and Ga-Ga spheres (**combined fitting**). Additionally, we performed a fitting in which we did not apply any constraints between the fitting parameters of the Ni and Ga K-edge data and only distinguished between Ni-M and Ga-M spheres (where M = Ni,Ga) (**independent fitting**). We performed EXAFS fittings of the catalysts $Ni_{65}Ga_{35}/SiO_2$-XAS and $Ni_{75}Ga_{25}/SiO_2$-XAS after in situ activation (and after cooling down to 50 °C in 1 bar H_2), of reacted $Ni_{65}Ga_{35}/SiO_2$-XAS after ca. 4h TOS under CO_2 hydrogenation conditions (after cooling down to 50 °C in 1 bar CO_2:N_2:H_2 = 1:1:3), and of ex-situ activated Ni_{100}/SiO_2 (room temperature).

Constraints applied to the fitted radial distances (r), coordination numbers (CN) and Debye Waller factors (σ^2) of the combined fitting:

(i) r(Ni-Ga) = r(Ga-Ni)
(ii) σ^2(Ni-Ga) = σ^2(Ga-Ni)
(iii) CN(Ni-Ga) = α*CN(Ga-Ni), where α = C_{Ga}/C_{Ni} which is the Ga:Ni (atomic) ratio estimated from PDF analysis
(iv) CN(Ga-Ga) = C_{Ga}*[CN(Ni-Ni) + CN(Ni-Ga)]

The constraints listed above assume that Ni and Ga are part of a random fcc-$Ni_yGa_{(100y)}$ alloy [where y:(100-y) is the Ni:Ga ratio in the alloy] containing atomic Ni and Ga fractions of C_{Ni} and C_{Ga}, respectively. A similar approach has been used in the literature to fit EXAFS of ceria-based solid solutions.[13] This approach assumes that Ni and Ga are equally coordinated (CN(Ni-Ni) + CN(Ni-Ga) = CN(Ga-Ni) + CN(Ga-Ga)) and that, on average, a fraction of C_{Ga} atoms in the (Ni, Ga) alloy are occupied by Ga.

Continuous Cauchy Wavelet Transform (CCWT) analysis of the k^2 weighted Ga EXAFS were performed using a freely available MATLAB script by Muñoz, Argoul and Farges.[14] The same k-ranges as reported above for the EXAFS fittings were used and the CCWT was calculated in the range 0.5 Å – 4 Å (Δr = 0.0175 Å). The Cauchy order of the WT was 50.

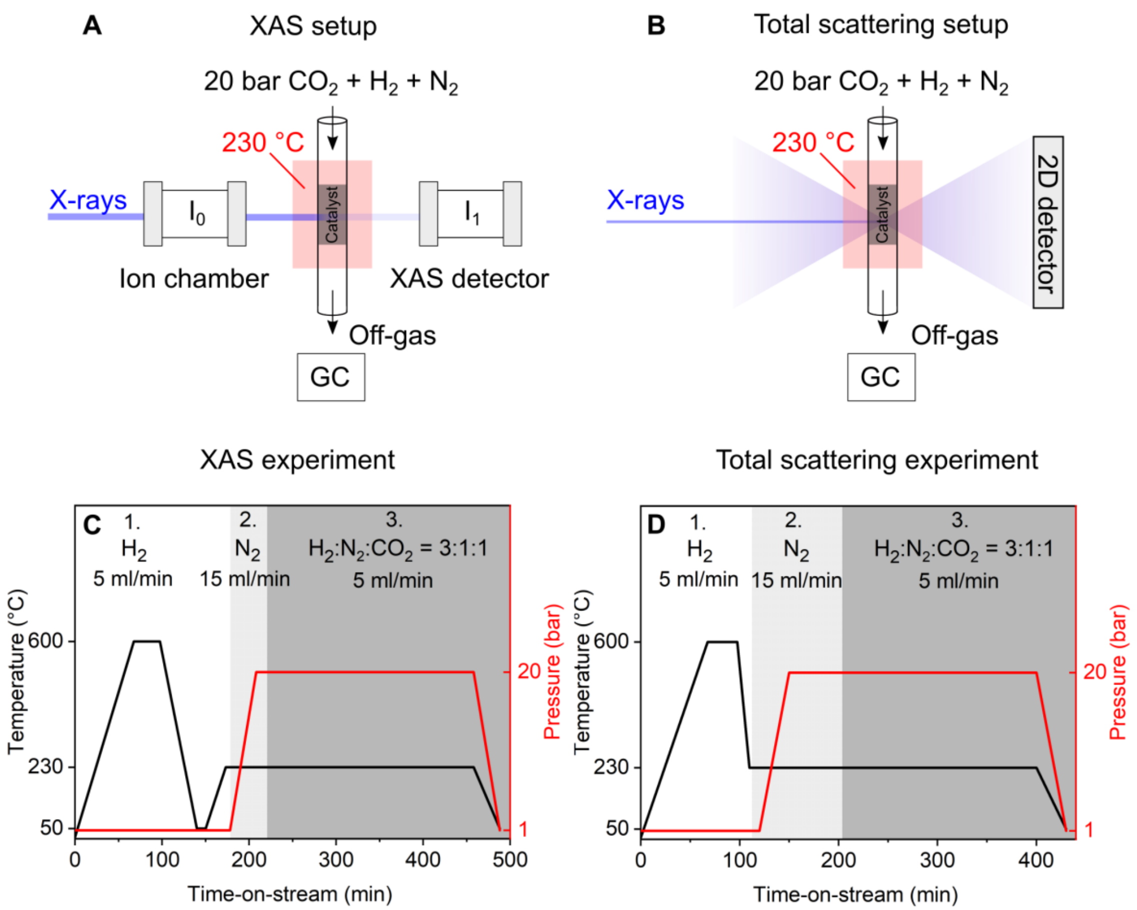

Figure S18. Schematic of the setups for in-situ/operando (A) XAS at BM31 and (B) total scattering experiments at ID15A; (C) and (D) specify the gas and temperature treatment steps during in situ/operando (C) XAS and (D) total scattering experiments where 1. Refers to the activation, 2. To the pressurizing and 3. To the reaction step.

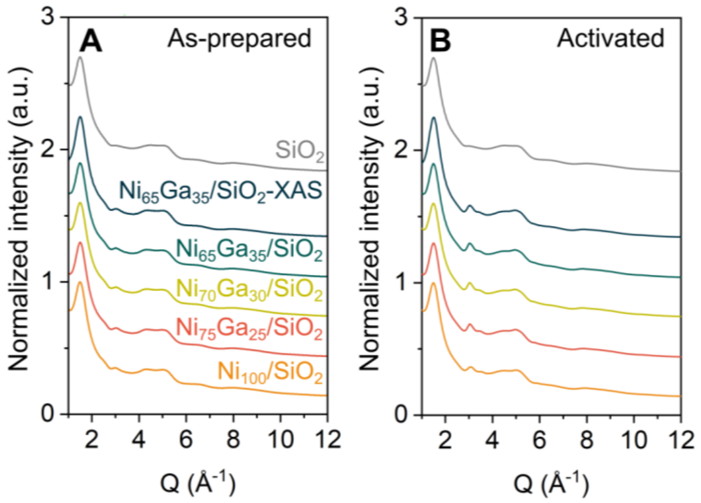

Figure S19. X-ray total scattering patterns of $Ni_xGa_{(100-x)}/SiO_2$ and SiO_2. (A) shows the as-prepared, air-exposed state (data collected at 50°C in 1 atm N_2) and (B) after in situ activation in H_2 (data collected at 230°C in H_2).

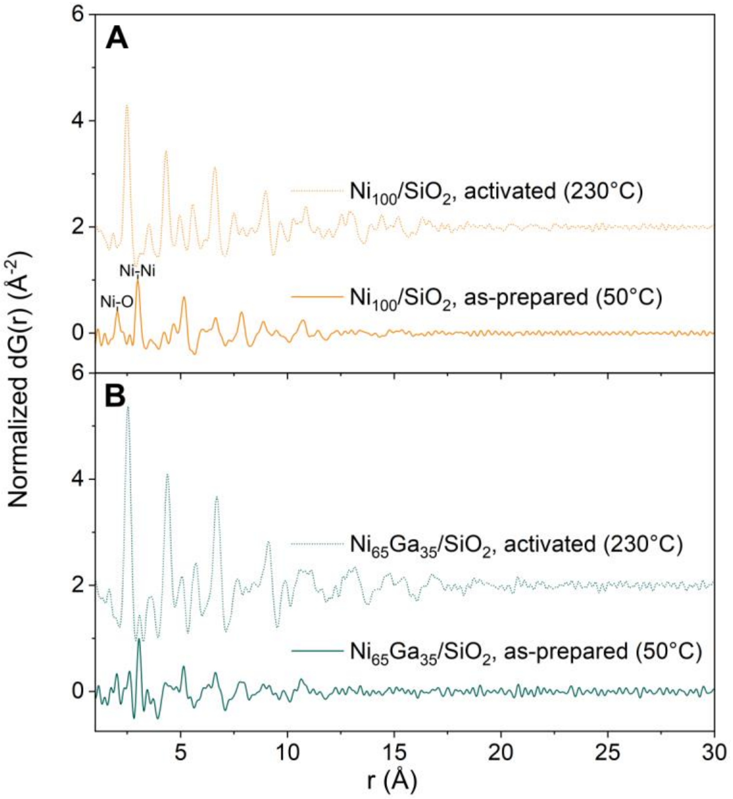

Figure S20. D-PDF of $Ni_{65}Ga_{35}/SiO_2$ and Ni_{100}/SiO_2. Solid lines show the as-prepared, air-exposed state (data collected at 50°C in 1 atm N_2) and (B) after in situ activation in H_2 (data collected at 230°C in H_2).

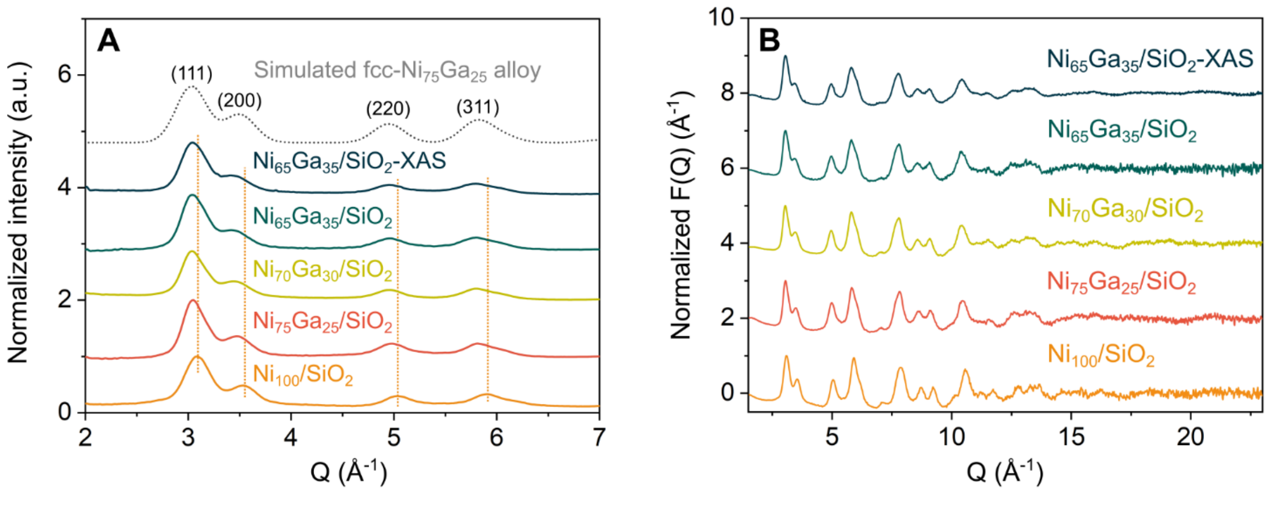

Figure S21. (A) SiO_2 subtracted X-ray total scattering patterns and a simulated fcc-$Ni_{75}Ga_{25}$ alloy pattern shown for reference. The dotted lines mark the positions of the Bragg peaks corresponding to Ni_{100}/SiO_2. (B) shows the respective reduced structure functions F(Q). Data were collected at 230 °C in 1 bar H_2 after in situ activation.

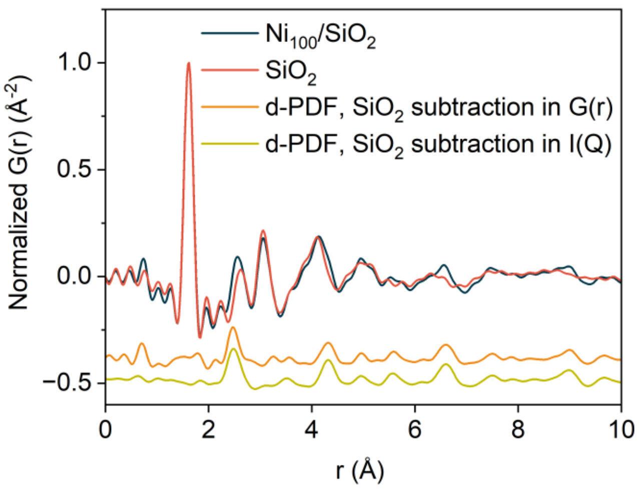

Figure S22. Approach to yield the differential PDF (d-PDF) at the example of Ni_{100}/SiO_2. The d-PDF can be calculated using two approaches, i) **subtraction in G(r)**, i.e. subtraction of the PDF of SiO_2 from the PDF of Ni_{100}, and by ii) **subtraction in I(Q)**: i.e. subtraction of the total scattering pattern of SiO_2 from the total scattering pattern of Ni_{100}, the result of which is then normalized and Fourier transformed to obtain the d-PDF. All of the d-PDFs reported in this work were obtained via the latter approach, as this resulted in d-PDFs with fewer noise ripples in the low r region (see r < 1.2 Å in the d-PDFs).

Table S3. Parameters obtained from fitting the d-PDF of in situ activated $Ni_xGa_{(100-x)}/SiO_2$ (data acquired at 230°C, Figure 2B) to a random fcc Ni-Ga alloy structure. All of the d-PDF were normalized by a factor of 10 and thus only the ratios of errors are significant but not the absolute values as they scale with the d-PDF normalization. ADF_i denotes the atomic displacement factor of i.[(a)]Atomic motion correlation factor denoted as *delta2* in PDFGui was used.[9] Q_{damp} and Q_{broad} were obtained from the fitting of a CeO_2 reference material.

**Material**	**Lattice parameter a = b = c (Å)**	**ADF_{Ni} = ADF_{Ga} ($Å^2$)**	**Atomic motion correlation factor[(a)] ($Å^2$)**	**Coherent particle size (Å)**	**Scale factor (-)**	**Weighted R value (-)**
Ni_{100}/SiO_2	3.529(2)	0.0142(7)	3.3(2)	21.5(5)	0.86(3)	0.1739
$Ni_{75}Ga_{25}/SiO_2$	3.570(2)	0.0160(8)	3.8(2)	21.6(5)	0.90(3)	0.1983
$Ni_{70}Ga_{30}/SiO_2$	3.584(2)	0.0154(7)	3.0(2)	20.8(4)	1.04(3)	0. 2090
$Ni_{65}Ga_{35}/SiO_2$	3.586(2)	0.0180(9)	3.6(2)	20.2(5)	1.02(3)	0.2319
$Ni_{65}Ga_{35}/SiO_2$-XAS	3.58(2)	0.021(8)	3.9(1)	20.1(4)	1.03(2)	0.2284

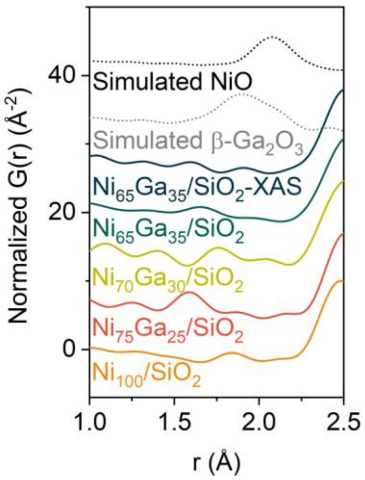

Figure S23. Zoom into the low r region (1-2.5 Å) of the d-PDF of $Ni_xGa_{(100-x)}/SiO_2$ after in situ activation (data collected at 230°C in 1 bar H_2) and simulated PDF of NiO (ICSD # 9866) and β-Ga_2O_3 (ICSD # 34243) for reference.

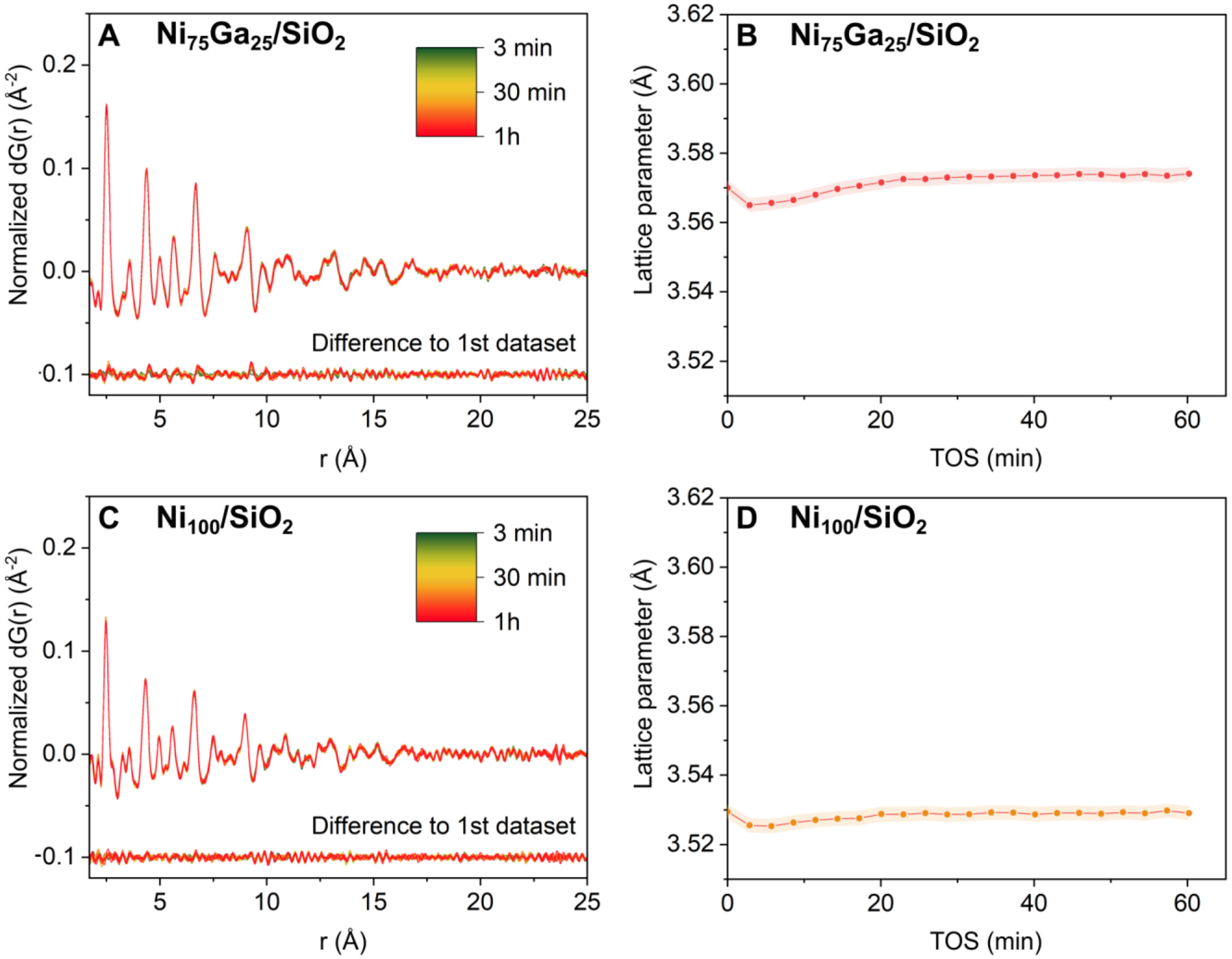

Figure S24. Evolution of the d-PDF of (A, B) $Ni_{75}Ga_{25}/SiO_2$, and (C,D) Ni_{100}/SiO_2 over ca. 1h TOS under CO_2 hydrogenation. Conditions: 20 bar CO_2:N_2:H_2 = 1:1:3, 230°C; (A,C) show d-PDF data and the difference between each scan with respect to the first data, whereas (B,D) plot the refined lattice parameters.

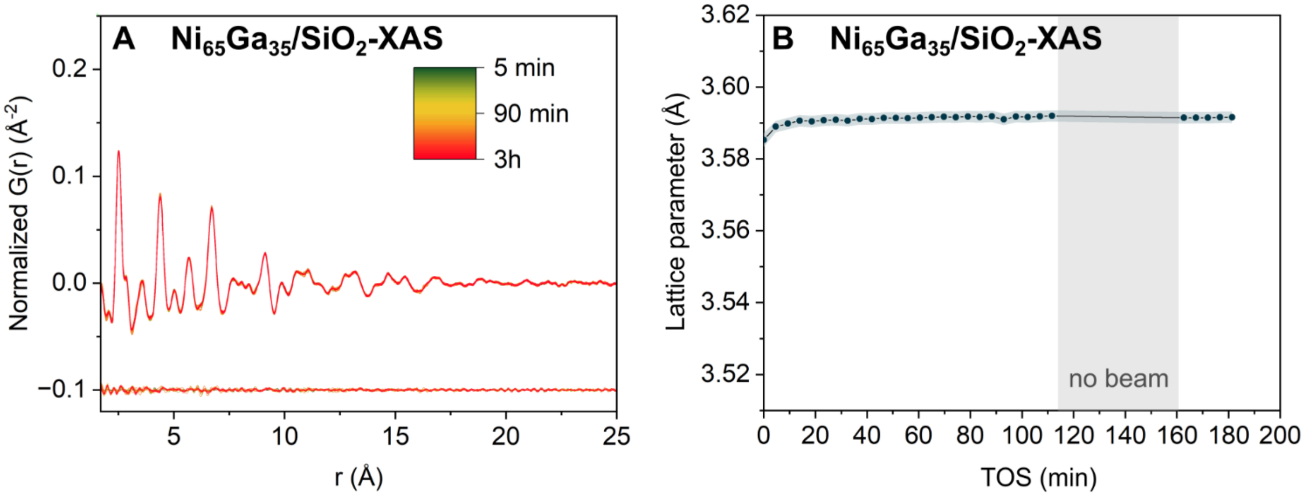

Figure S25. Evolution of (A) the d-PDF and (B) the fitted lattice parameters of $Ni_{65}Ga_{35}/SiO_2$-XAS over ca. 3h under CO_2 hydrogenation conditions. Conditions: 20 bar CO_2:N_2:H_2 = 1:1:3, 230°.

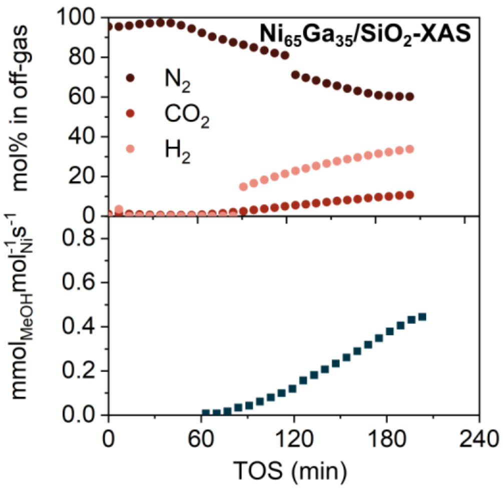

Figure S26. Off-gas composition measured via a GC during the operando X-ray total scattering - CO_2 hydrogenation experiment using $Ni_{65}Ga_{35}/SiO_2$-XAS (PDF data shown in Figure S25). Conditions: 20 bar CO_2:N_2:H_2 = 1:1:3, 230°C.

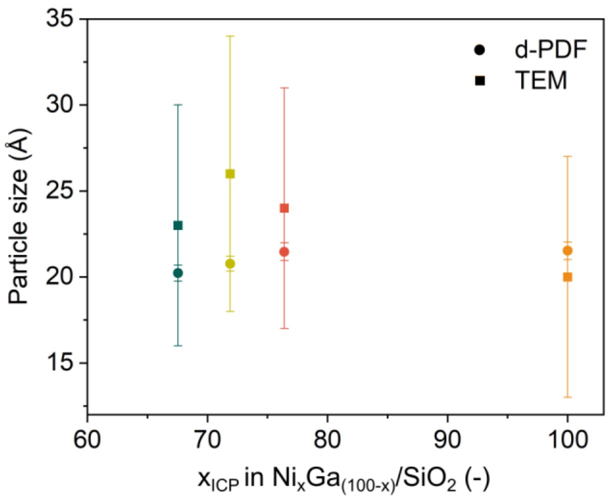

Figure S27. Particle (crystallite) size obtained from the d-PDF fitting of activated $Ni_{65}Ga_{35}/SiO_2$, $Ni_{70}Ga_{30}/SiO_2$, $Ni_{75}Ga_{25}/SiO_2$, and Ni_{100}/SiO_2, compared to the particle size obtained from TEM analysis of the as-prepared materials. The error bars represent the standard deviation.

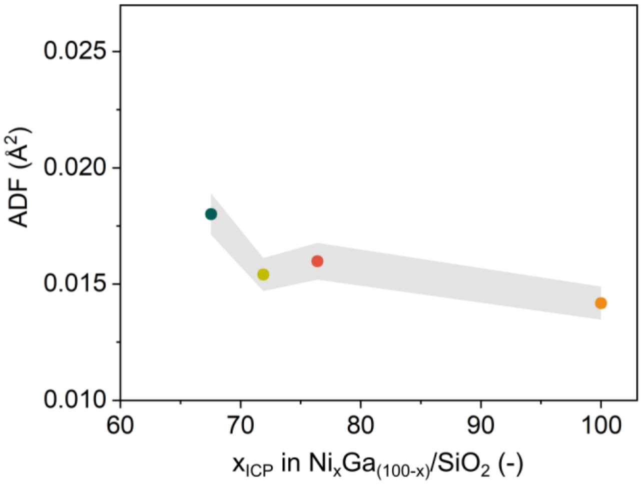

Figure S28. Refined atomic displacement factors (ADF) as a function of x_{ICP} in $Ni_{65}Ga_{35}/SiO_2$, $Ni_{70}Ga_{30}/SiO_2$, $Ni_{75}Ga_{25}/SiO_2$, and Ni_{100}/SiO_2. The shaded line represents the standard deviation obtained from the d-PDF fitting analysis.

Table S4. Literature data of cell parameters for Ni and Ni-Ga fcc alloys used to derive the slope of Vegard's law and available in the ICSD.

Material	Space group	ICSD #	Lattice parameter (Å)	Reference
Ni	*Fm*-3*m*	8688	3.5239	[15]
$Ni_{92}Ga_{08}$		103859	3.543	[16]
$Ni_{92}Ga_{08}$		634846	3.542	[17]
$Ni_{92}Ga_{08}$		634862	3.545	[18]
$Ni_{85}Ga_{15}$		634848	3.557	[19]
$Ni_{70}Ga_{30}$		103862	3.591	[20]

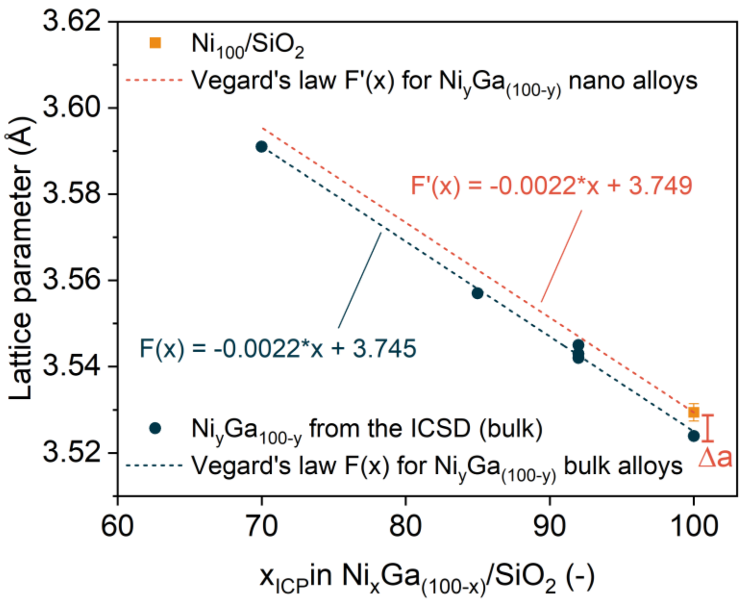

Figure S29. Vegard's law for $Ni_yGa_{(100-y)}$ bulk alloys (blue dashed curve, F(x)) derived from the cell parameters reported in Table S4. Vegard's law for the $Ni_yGa_{(100-y)}$ nano alloys (red dashed curve, F'(x)) is obtained by applying a vertical shift of Δa = 0.004 Å to F(x).

Table S5. Absolute and Ni normalized $Ga_{alloyed}$ and GaO_x contents in the catalysts, as determined by PDF and ICP analyses.

**Catalyst**	**x_{ICP} in $Ni_xGa_{(100-x)}$ (-)**	**Molar ratio of Ni:Ga in the fcc-$Ni_yGa_{(100-y)}$ alloy (-)**	**$Ga_{alloyed}$ (µmol)**	**GaO_x (µmol)**	**$Ga_{alloyed}$ ($mol_{Gaalloyed}$ mol_{Ni}^{-1})**	**GaO_x (mol_{GaOx} mol_{Ni}^{-1})**
$Ni_{65}Ga_{35}/SiO_2$	67.5	74:26	0.20	0.08	0.34	0.14
$Ni_{70}Ga_{30}/SiO_2$	71.9	75:25	0.14	0.02	0.33	0.06
$Ni_{75}Ga_{25}/SiO_2$	76.4	82:18	0.10	0.04	0.23	0.08
Ni_{100}/SiO_2	100	100:0	-	-	-	-

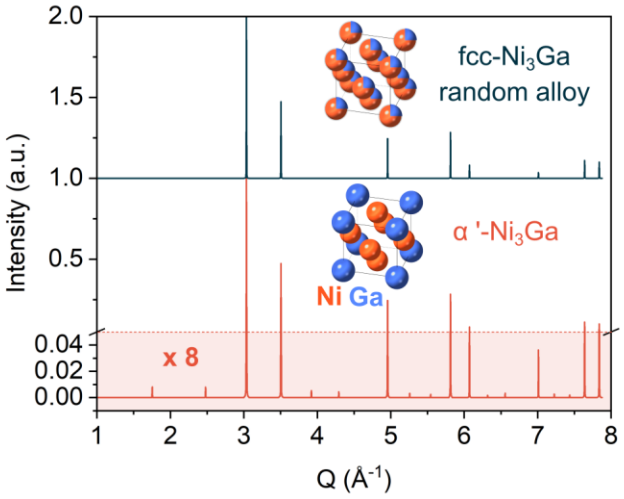

Figure S30. Simulated X-ray diffraction patterns of a fcc-Ni_3Ga random alloy (top) and the intermetallic α'-Ni_3Ga (bottom) (*Pm*-3*m* space group) with their respective unit cells. Below the red dotted line, the intensities are magnified by a factor of 8.

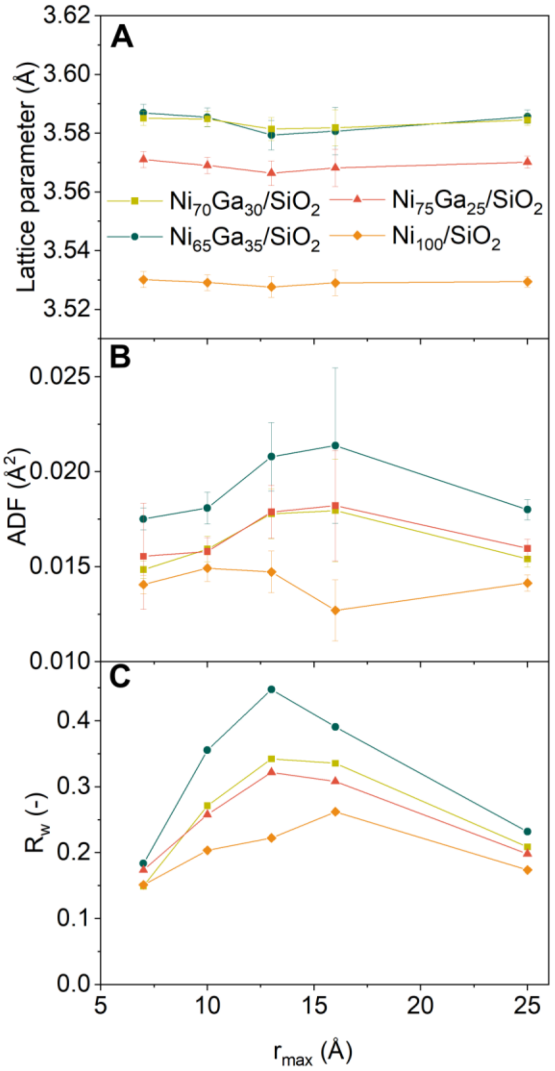

Figure S31. (A) Lattice parameters, (B) atomic displacement factors (ADF), and (C) weighted R values extracted from a boxcar fitting between 2-7, 5-10, 8-13, 11-16 Å, and 1.7-25 Å for, respectively, $Ni_{65}Ga_{35}/SiO_2$, $Ni_{70}Ga_{30}/SiO_2$, $Ni_{75}Ga_{25}/SiO_2$, and Ni_{100}/SiO_2.

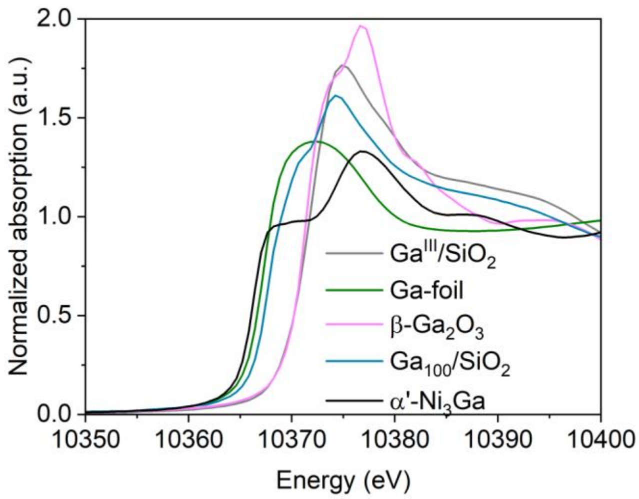

Figure S32. Ga K-edge XANES of the reference materials used for the LCF analysis.

Table S6. Results of the LCF performed on the $Ni_{65}Ga_{35}/SiO_2$-XAS and $Ni_{25}Ga_{75}/SiO_2$-XAS Ga K-edge XANES data, using different references (C1 = α'-Ni_3Ga or Ga-foil, C2 = Ga_{100}/SiO_2, Ga^{III}/SiO_2, or β-Ga_2O_3). The best agreement between data and fit was obtained with C1 α'-Ni_3Ga and C2 = Ga_{100}/SiO_2.

Material	C1	C2	LCF weight fraction C1 (-)	LCF weight fraction C2 (-)	R-factor
$Ni_{65}Ga_{35}/SiO_2$-XAS	α'-Ni_3Ga	Ga_{100}/SiO_2	**0.69(1)**	**0.31(1)**	**0.0026**
		Ga^{III}/SiO_2	0.90(2)	0.10(2)	0.0109
		β-Ga_2O_3	0.91(1)	0.09(1)	0.0112
	Ga-foil	Ga_{100}/SiO_2	0.57(5)	0.43(4)	0.0275
		Ga^{III}/SiO_2	0.76(3)	0.24(2)	0.0232
		β-Ga_2O_3	0.79(2)	0.21(2)	0.0258
$Ni_{25}Ga_{75}/SiO_2$-XAS	α'-Ni_3Ga	Ga_{100}/SiO_2	**0.81(1)**	**0.19(1)**	**0.0023**
		Ga^{III}/SiO_2	0.94(1)	0.06(1)	0.0056
		β-Ga_2O_3	0.95(1)	0.05(1)	0.0059
	Ga-foil	Ga_{100}/SiO_2	0.63(6)	0.37(5)	0.0436
		Ga^{III}/SiO_2	0.77(3)	0.23(2)	0.0357
		β-Ga_2O_3	0.80(2)	0.20(2)	0.0390

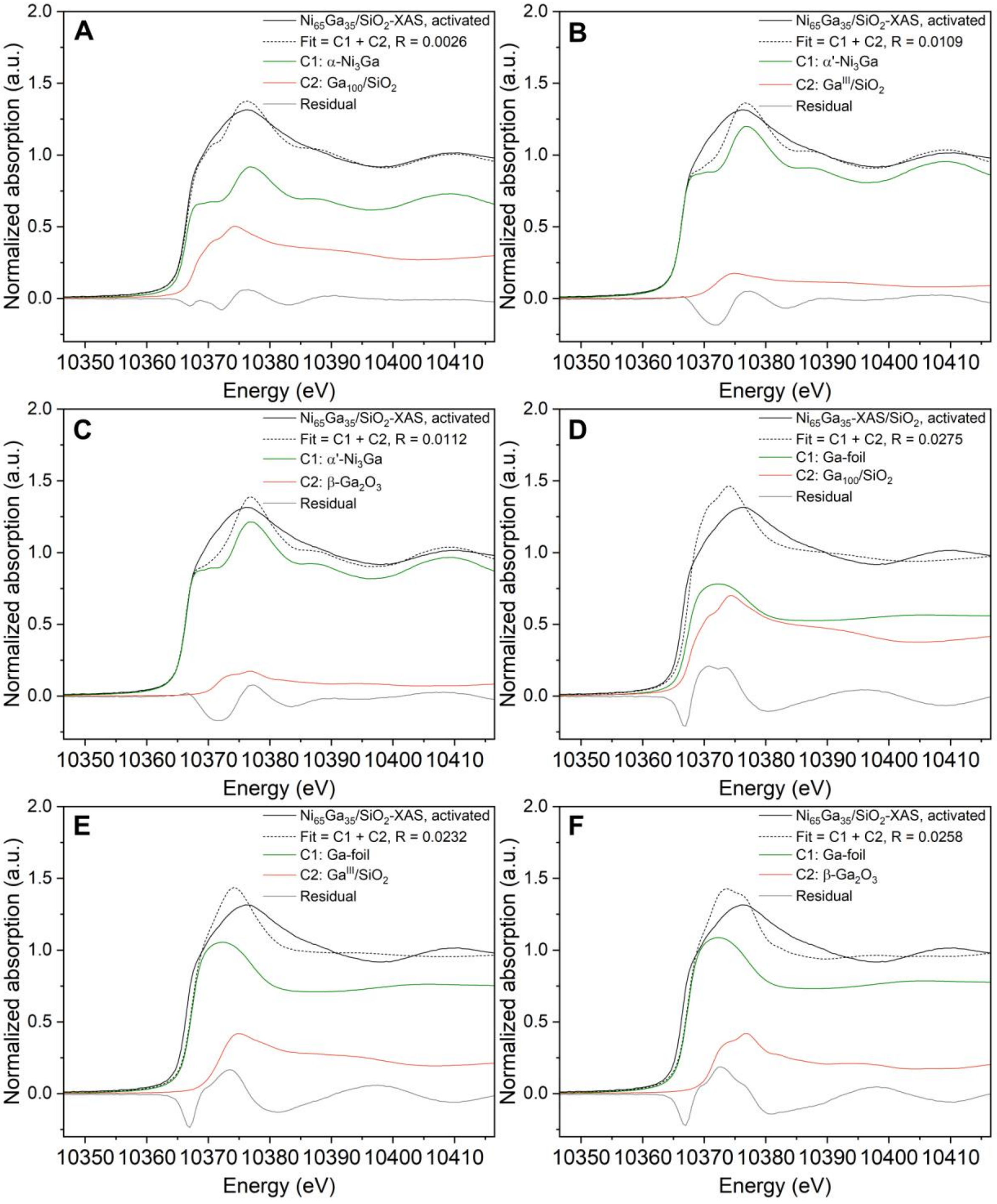

Figure S33. LCF of the Ga K-edge XANES of in situ activated $Ni_{65}Ga_{35}/SiO_2$-XAS to different combinations of reference components, C1 and C2 (C1 = α'-Ni_3Ga or Ga-foil, C2 = Ga_{100}/SiO_2, Ga^{III}/SiO_2, or β-Ga_2O_3). The best agreement is obtained for (A): C1 = α'-Ni_3Ga and C2 = Ga_{100}/SiO_2.

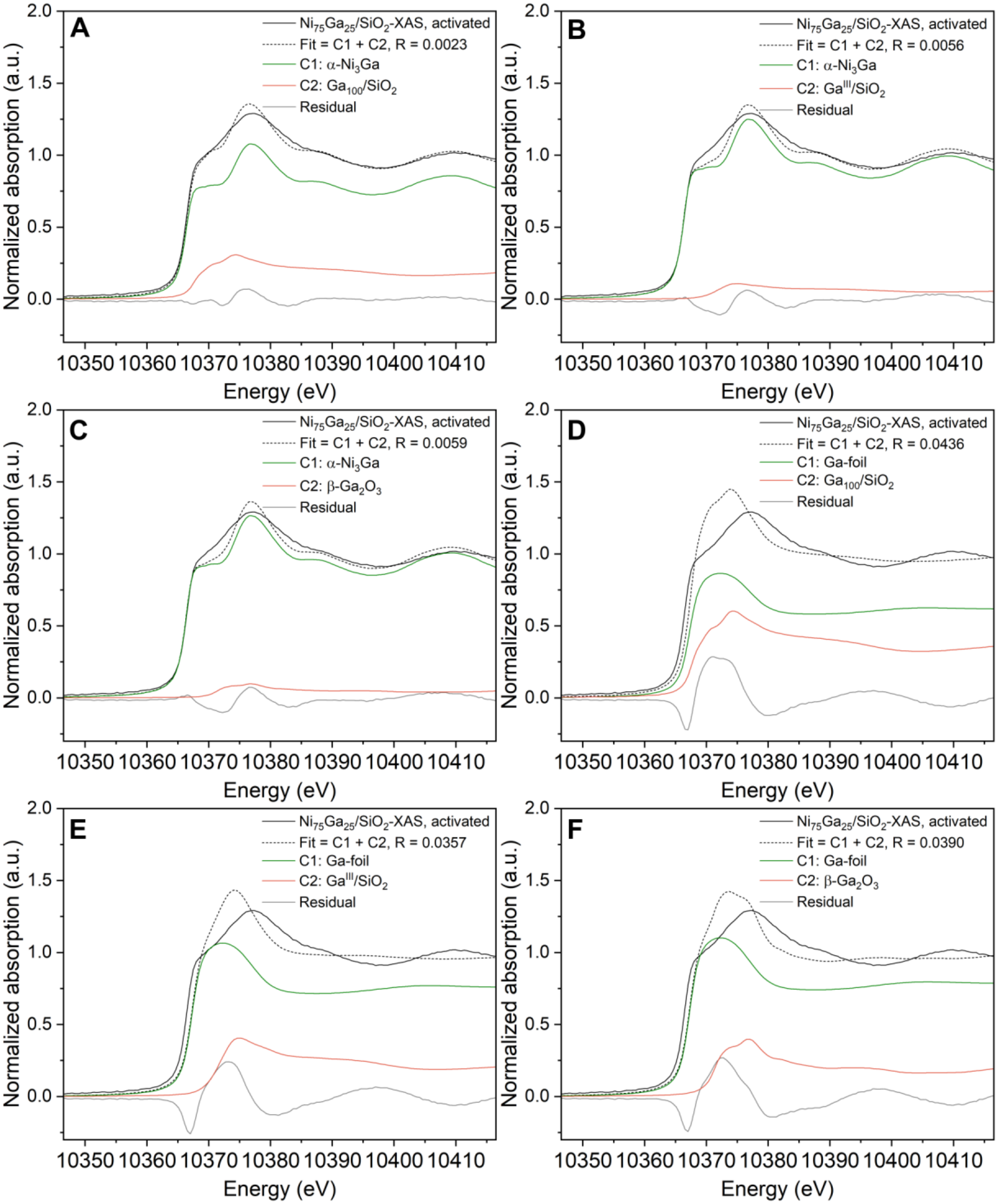

Figure S34. LCF of the Ga K-edge XANES of in situ activated $Ni_{75}Ga_{25}/SiO_2$-XAS to different combinations of reference materials C1 and C2 (C1 = α’-Ni_3Ga or Ga-foil, C2 = Ga_{100}/SiO_2, Ga^{III}/SiO_2, or β-Ga_2O_3). The best agreement is obtained for (A): C1 = α’-Ni_3Ga and C2 = Ga_{100}/SiO_2.

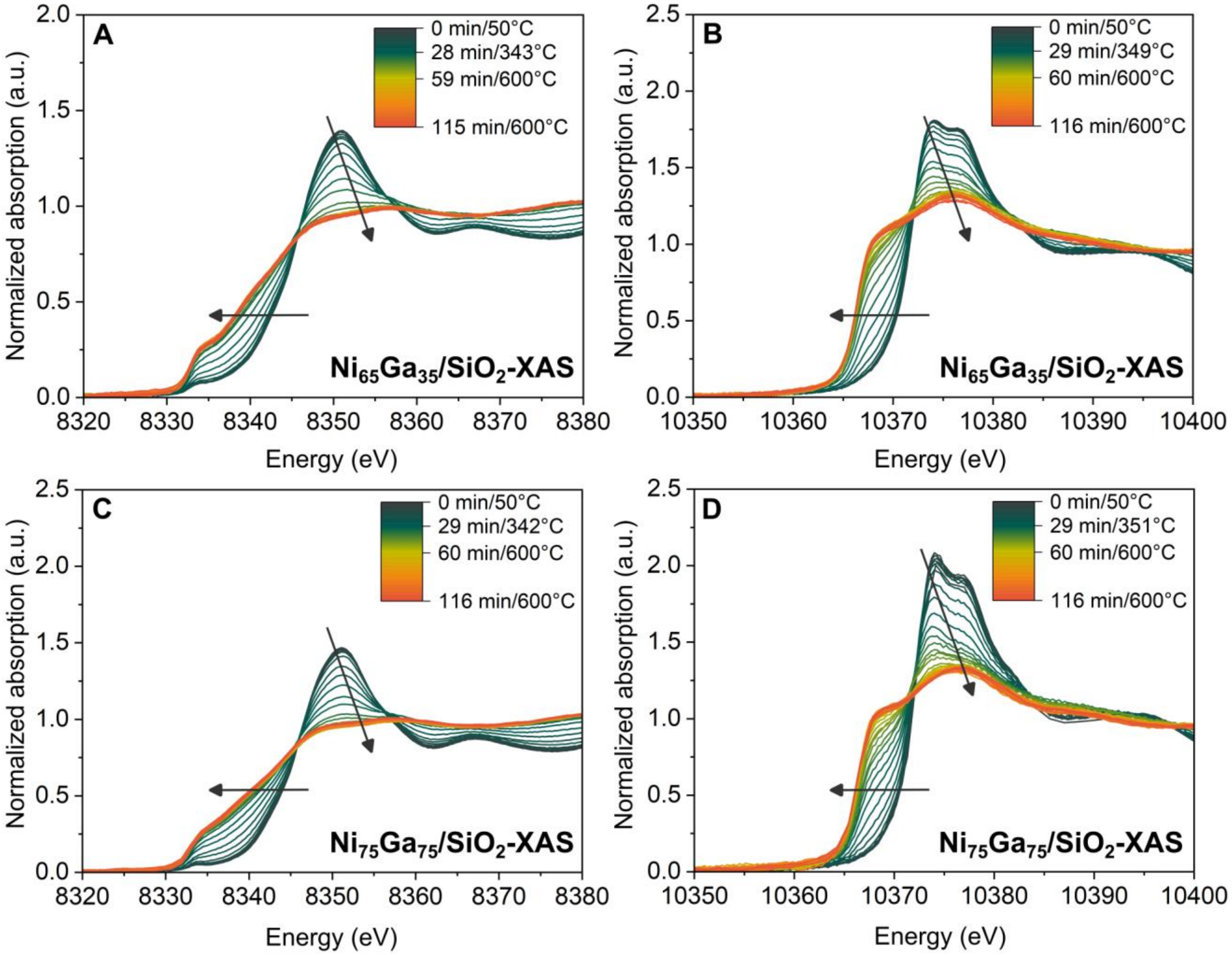

Figure S35. Ni and Ga K-edges XANES of (A and B) $Ni_{65}Ga_{35}/SiO_2$-XAS and (C-D) $Ni_{75}Ga_{25}/SiO_2$-XAS exposed to air during in situ activation (temperature ramp from 50°C to 600°C (10°/min) in 1 bar H_2). The arrows show the direction of the changes.

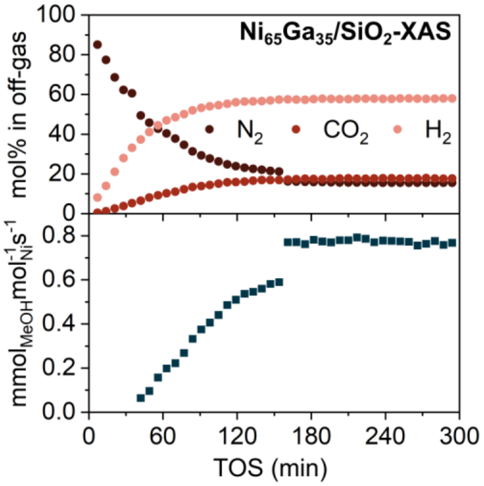

Figure S36. Off-gas composition measured during an operando XAS - CO_2 hydrogenation experiment using $Ni_{65}Ga_{35}/SiO_2$-XAS (XAS data shown in Figure 5C-D). Conditions: 20 bar CO_2:N_2:H_2 = 1:1:3, 230°C.

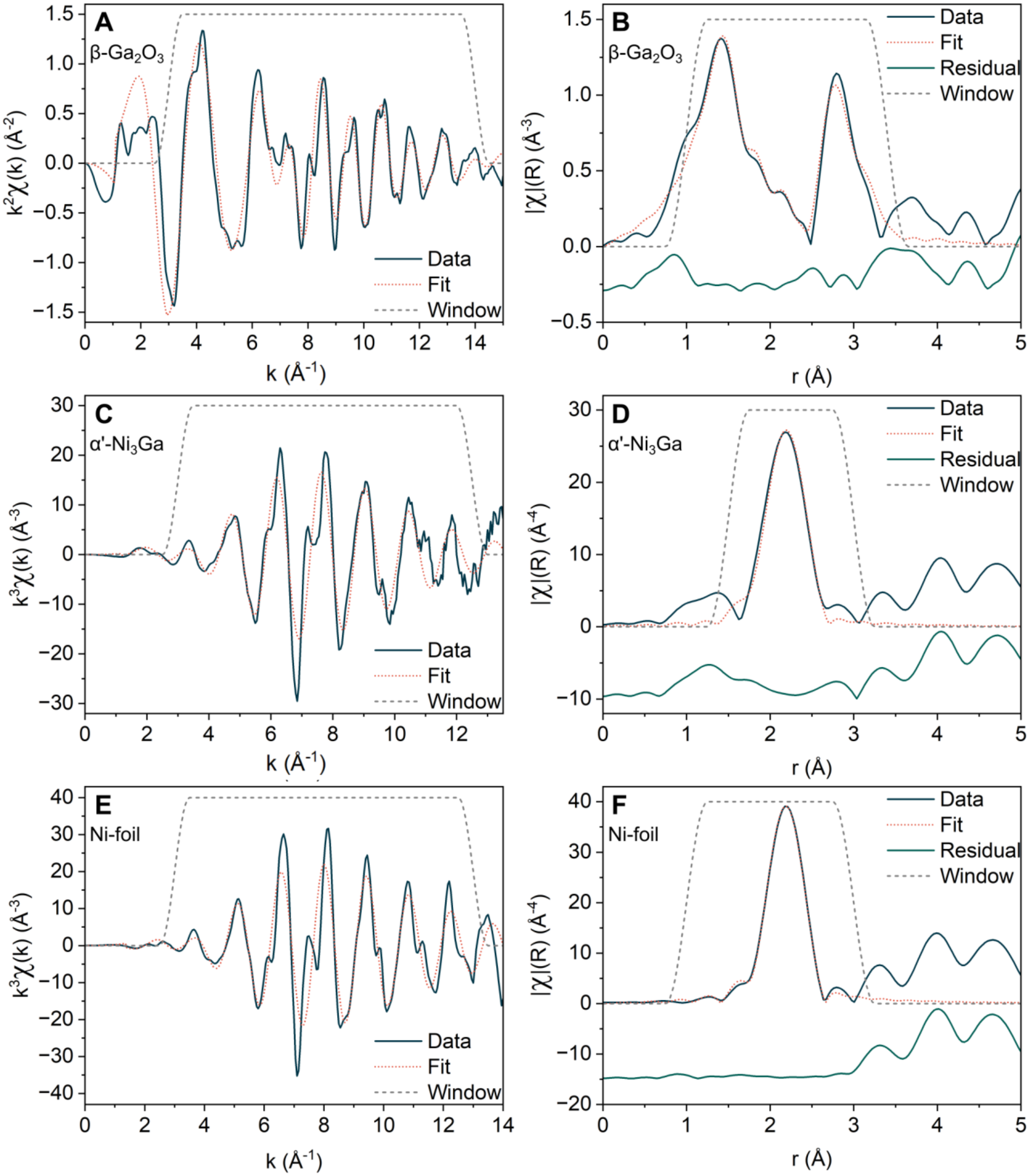

Figure S37. EXAFS experimental data and fittings (k and r space) of the reference materials used to determine S_0^2 at the Ga (β-Ga_2O_3: A-B, α'-Ni_3Ga: C-D) and Ni (E-F) K-edges. Data collected at room temperature in ambient air.

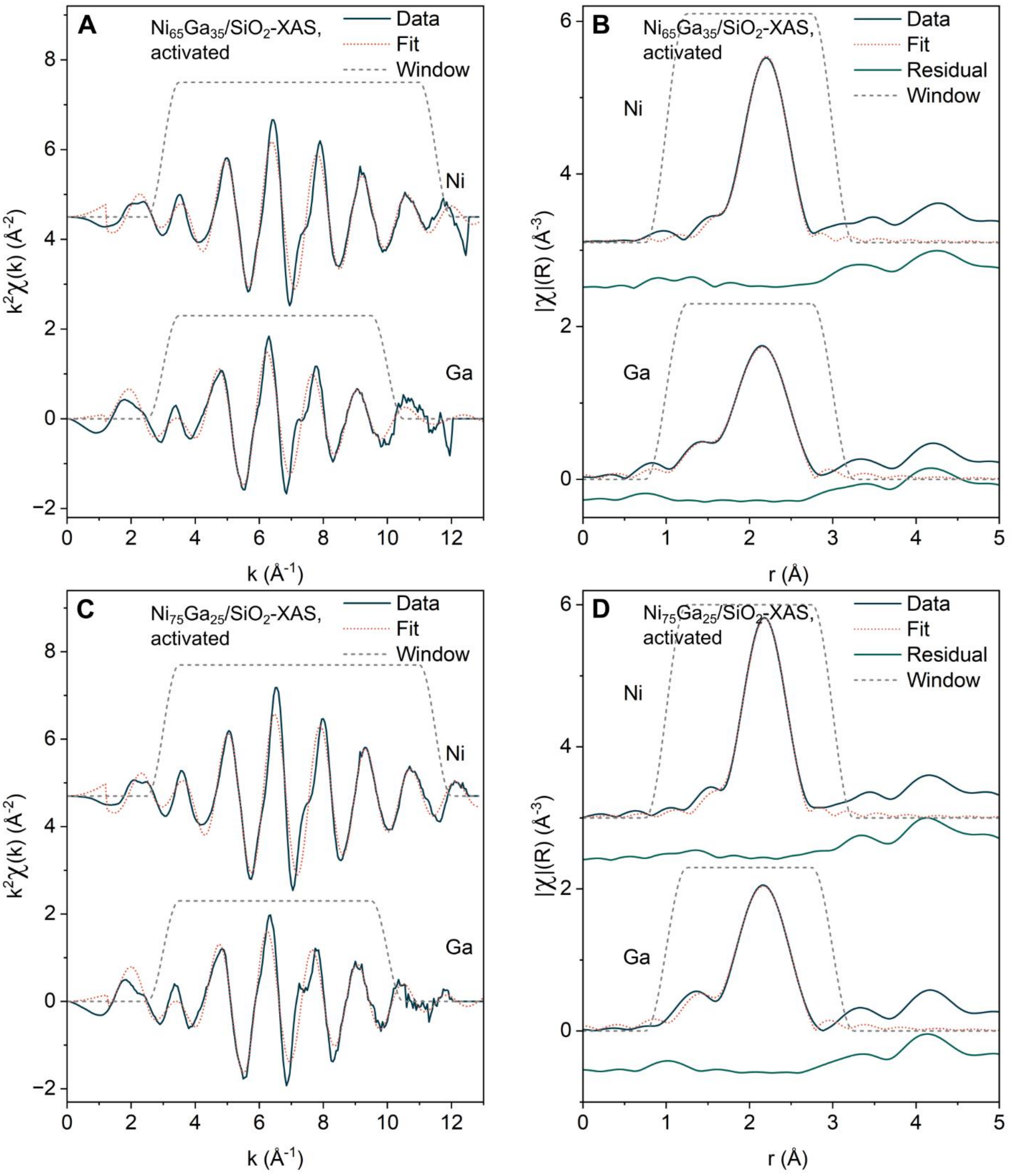

Figure S38. EXAFS experimental data and independent fittings (k and r space) of in situ activated $Ni_{65}Ga_{35}/SiO_2$-XAS and $Ni_{75}Ga_{25}/SiO_2$-XAS (data collected in situ at 50 °C, H_2).

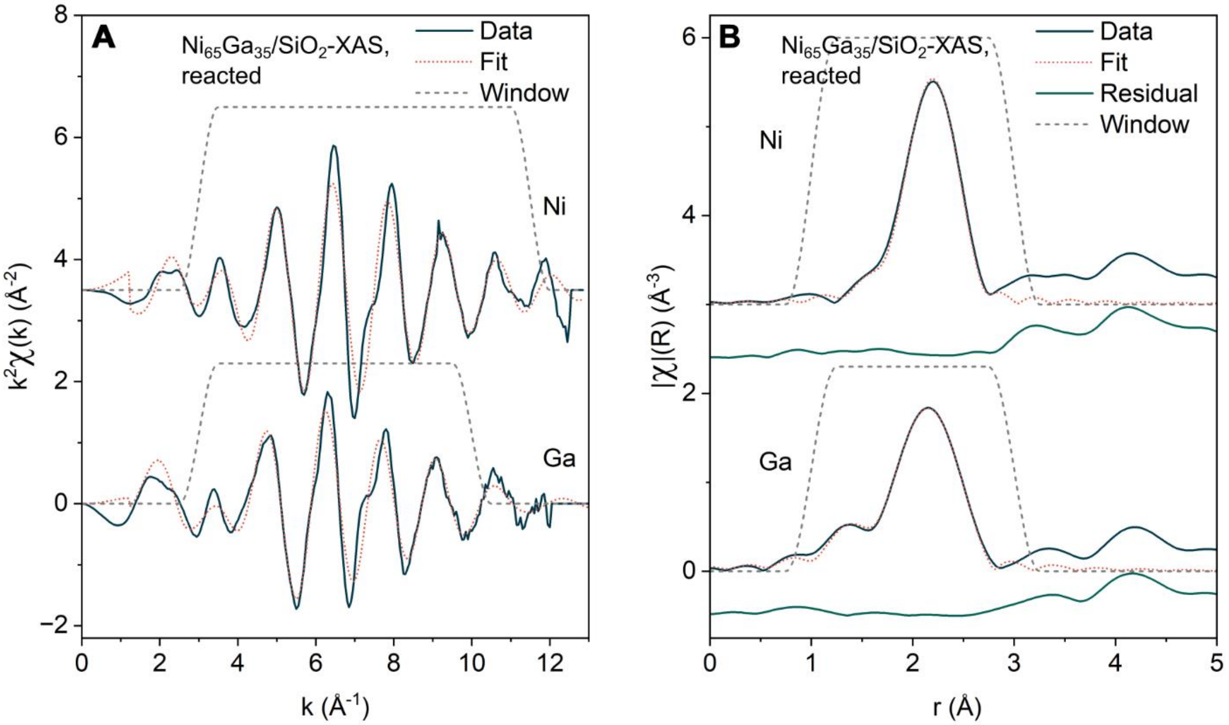

Figure S39. EXAFS experimental data and fittings (k and r space) of $Ni_{65}Ga_{35}/SiO_2$-XAS after ca. 4 h of reaction at 20 bar, $CO_2:H_2:N_2$ = 1:3:1, 230°C (data collected at 50°C, $CO_2:H_2:N_2$ = 1:3:1).

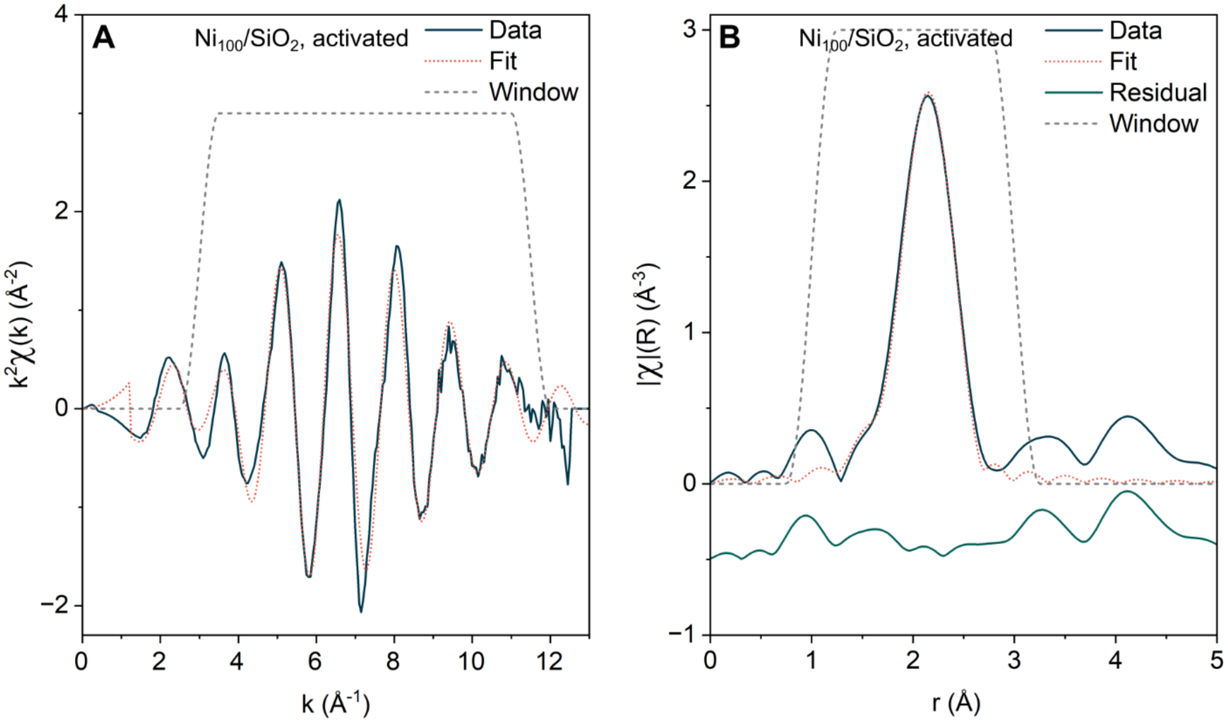

Figure S40. EXAFS experimental data and fittings (k and r space) of in situ activated Ni_{100}/SiO_2, measured ex-situ (data collected at room temperature, N_2 atmosphere).

Table S7. EXAFS fitting results of the reference materials used to determine the amplitude reduction factor S_0^2 at the Ni (Ni-foil) and Ga K-edge (α'-Ni_3Ga and β-Ga_2O_3). The errors on the reported values as estimated by the software Artemis are given in compact (crystallographic) notation, i.e., 2.52(1) corresponds to 2.52±0.01. Parameters without errors were fixed. Abbreviations: Rw: weighted R factor, ΔE_0: the edge energy shift, CN: coordination number, σ^2: Debye-Waller factor, r: interatomic distance. Ga-O_{tet} and Ga-O_{oct} denote Ga which is tetrahedrally and octahedrally coordinated by O.

**Material**	**Rw (-)**	**S_0^2 (-)**	**ΔE_0(eV)**	**Structural parameters**											
				**CN (Ga-O_{tet}) (-)**	**r(Ga-O_{tet}) (Å)**	**σ^2(Ga-O_{tet}) (Å^2)**	**CN(Ga-O_{oct}) (-)**	**r(Ga-O_{oct}) (Å)**	**σ^2(Ga-O_{oct}) (Å^2)**	**CN(Ga-Ga_{tet}) (-)**	**r(Ga-Ga_{tet}) (Å)**	**σ^2(Ga-Ga_{tet}) (Å^2)**	**CN(Ga-Ga_{oct}) (-)**	**r(Ga-Ga_{oct}) (Å)**	**σ^2(Ga-Ga_{oct}) (Å^2)**
β-Ga_2O_3	0.0189	0.91 (1)	4(2)	2	1.85 (3)	0.003 (2)	3	2.00 (8)	0.009 (5)	3.5	3.049 (9)	0.005 (1)	4.5	3.34 (5)	0.010(2)
				**CN(Ga-Ni) (-)**				**r(Ga-Ni) (Å)**				**σ^2(Ga-Ni) (Å^2)**			
α' - Ni_3Ga (Ga K-edge)	0.0107	0.93 (9)	5(1)	12				2.52(1)				0.009(9)			
				**CN(Ni-Ni) (-)**				**r(Ni-Ni) (Å)**				**σ^2(Ni-Ni) (Å^2)**			
Ni-foil	0.0019	0.82 (3)	7.2(4)	12				2.483(8)				0.0061(3)			

Table S8. Independent EXAFS fitting results. Notation of the errors and abbreviations are as in Table S7. (a)The Debye-Waller factor of Ga-O was set to the value obtained from β-Ga_2O_3 for tetrahedrally coordinated Ga-O (σ^2(Ga-O_{tet})). M = Ni/Ga for $Ni_{65}Ga_{35}/SiO_2$ and $Ni_{75}Ga_{25}/SiO_2$, and M = Ni for Ni_{100}/SiO_2

Material	CN (Ga-O) (-)	σ^2 (Ga-O) (Å^2)(a)	r(Ga-O) (Å)	CN (Ni-M) (-)	σ^2(Ni-M) (Å^2)	r(Ni-M) (Å)	CN (Ga-M) (-)	σ^2(Ga-M) (Å^2)	r(Ga-M) (Å)	ΔE_{Ni} (eV)	ΔE_{Ga} (eV)	Rw_{Ni} (-)	Rw_{Ga} (-)
$Ni_{65}Ga_{35}/SiO_2$-XAS, activated	0.7(1)	0.003	1.87(5)	8.8(6)	0.0088(6)	2.529(6)	8.1(8)	0.012(1)	2.52(2)	5.2(6)	6(1)	0.0056	0.0029
$Ni_{65}Ga_{35}/SiO_2$-XAS, reacted	0.7(1)	0.003	1.86(4)	9.2(7)	0.0089(7)	2.52(1)	8.0(8)	0.011(1)	2.52(2)	5.5(8)	6(1)	0.0057	0.0044
$Ni_{75}Ga_{25}/SiO_2$-XAS, activated	0.6(3)	0.003	1.84(2)	9.0(6)	0.0079(6)	2.50(3)	9(2)	0.011(2)	2.52(1)	5.8(7)	7(2)	0.0058	0.0058
Ni_{100}/SiO_2, activated	n.a.	n.a.	n.a.	9(1)	0.009(1)	2.48 (1)	n.a.	n.a.	n.a.	6(1)	n.a.	0.0127	n.a.

Table S9. Combined Ni and Ga K-edge EXAFS fitting results. Notation of the errors and abbreviations are as in Table S7. α is the Ga:Ni ratio obtained from PDF analysis. (a)The Debye-Waller factor of Ga-O was set to the value obtained from β-Ga_2O_3 for tetrahedrally coordinated Ga-O (σ^2(Ga-O_{tet})).

Material	CN(Ga-O) (-)	σ^2 (Ga-O) (Å^2)(a)	r(Ga-O) (Å)	CN(Ni-Ni) (-)	σ^2(Ni-Ni) (Å^2)	r(Ni-Ni) (Å)	CN(Ga-Ni) (-)	σ^2(Ga-Ni) = σ^2(Ni-Ga) (Å^2)	r(Ga-Ni) = r(Ni-Ga) (Å)	CN(Ni-Ga) = α*CN(Ga-Ni) (-)	CN(Ga-Ga)(b) (-)	σ^2(Ga-Ga) (Å^2)	r(Ga-Ga) (Å)	ΔE_{Ni} (eV)	ΔE_{Ga} (eV)	Rw_{tot} (-)
$Ni_{65}Ga_{35}/SiO_2$-XAS, activated	0.7(1)	0.003	1.86 (4)	7.8(8)	0.010(1)	2.544(9)	6(1)	0.007 (3)	2.48 (6)	1.9	2.5	0.005(4)	2.59 (6)	6(1)	5(2)	0.0033
$Ni_{65}Ga_{35}/SiO_2$-XAS, reacted	0.8(1)	0.003	1.85 (2)	8(1)	0.010(1)	2.535(2)	6(1)	0.007 (3)	2.48 (6)	1.9	2.7	0.005(4)	2.59 (6)	6(1)	6(2)	0.0033
$Ni_{75}Ga_{25}/SiO_2$-XAS, activated	0.9(4)	0.003	1.86 (3)	7.7(7)	0.008(1)	2.50 (3)	9(5)	0.01 (1)	2.51 (3)	1.7	1.5	0.005(8)	2.51 (2)	5.9(8)	6(4)	0.0063

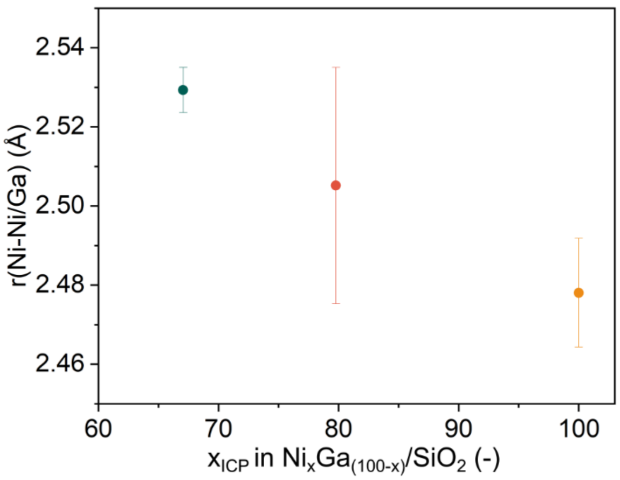

Figure S41. Fitted Ni-Ni/Ga distance as a function of x_{ICP} in the activated $Ni_xGa_{(100-x)}/SiO_2$ (data collected in situ at 50 °C, H_2).

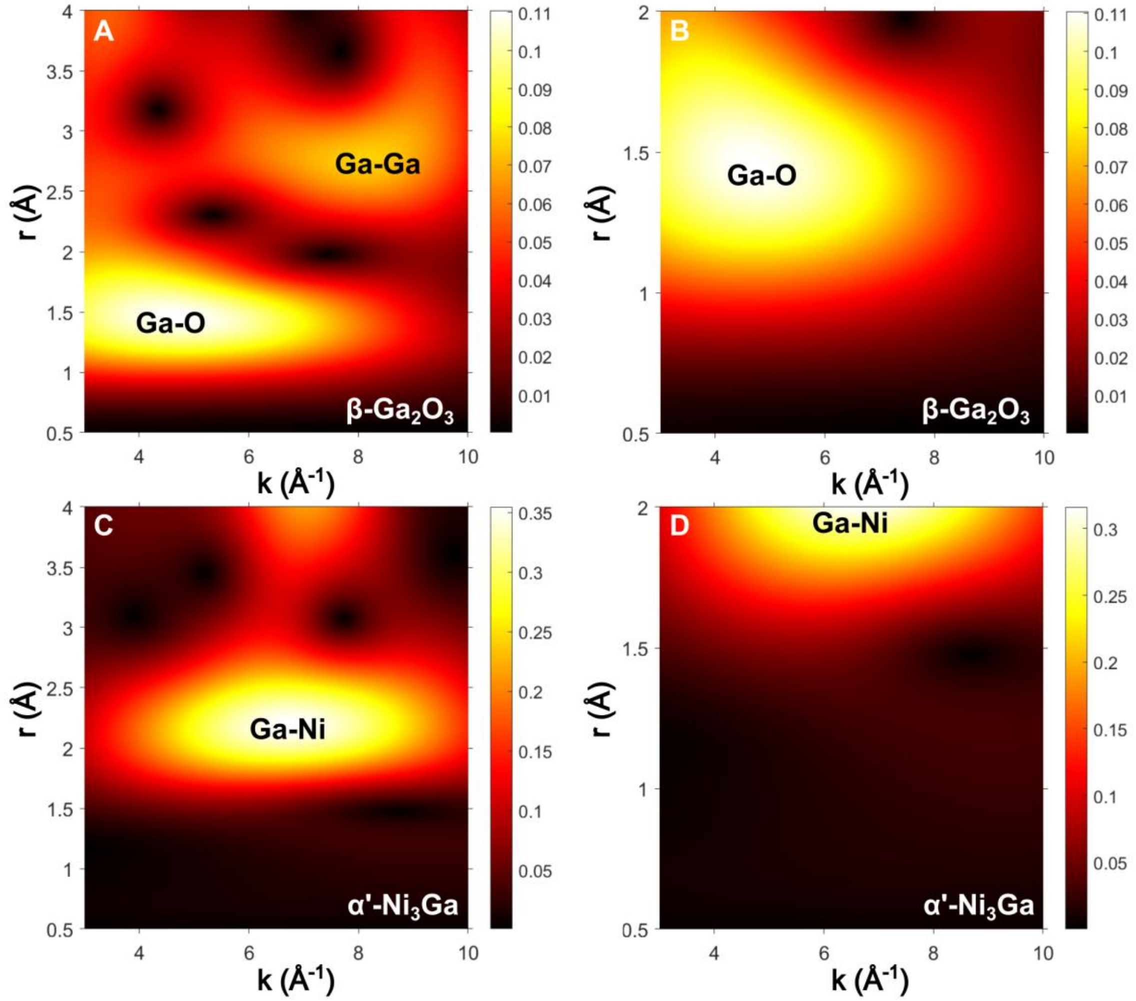

Figure S42. Continuous Cauchy wavelet transform of the k^2 weighted Ga K edge EXAFS of β-Ga_2O_3 (A-B) and α'-Ni_3Ga (C-D). B and D show an inset in the r-region 0.5-2 Å (Ga-O).

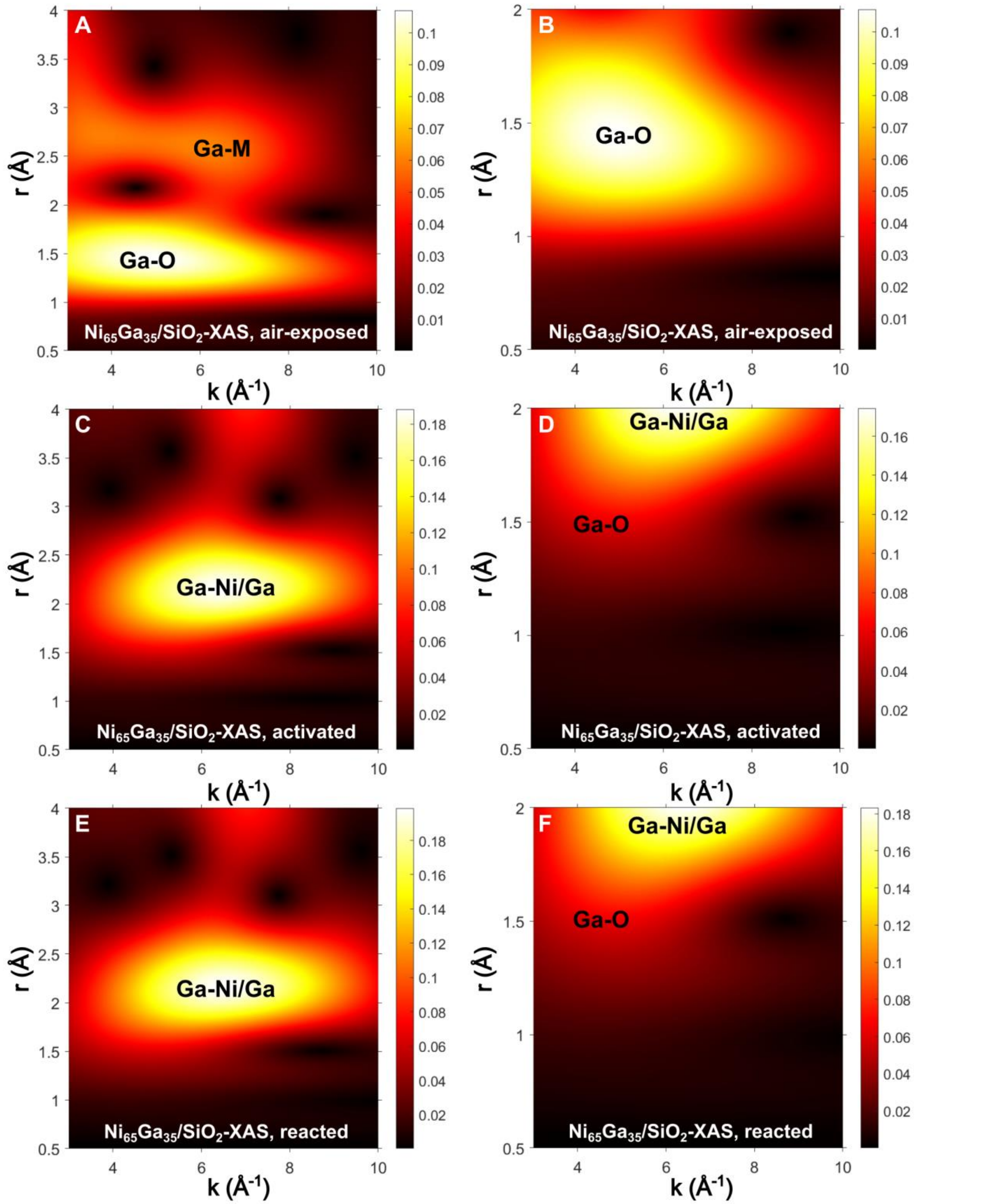

Figure S43. Continuous Cauchy wavelet transform of the k^2 weighted Ga K edge EXAFS of air-exposed (A-B, data collected at 50°C in 1bar H_2), in situ activated (C-D, data collected at 50°C in 1 bar H_2), and reacted (E-F, data collected at 50°C in 1 bar CO_2:H_2:N_2 = 1:3:1) $Ni_{65}Ga_{35}/SiO_2$-XAS. B, D and F show an inset in the r-region 0.5-2 Å (Ga-O). In Ga-M in A), M stands for Ni, Ga and/or Si.

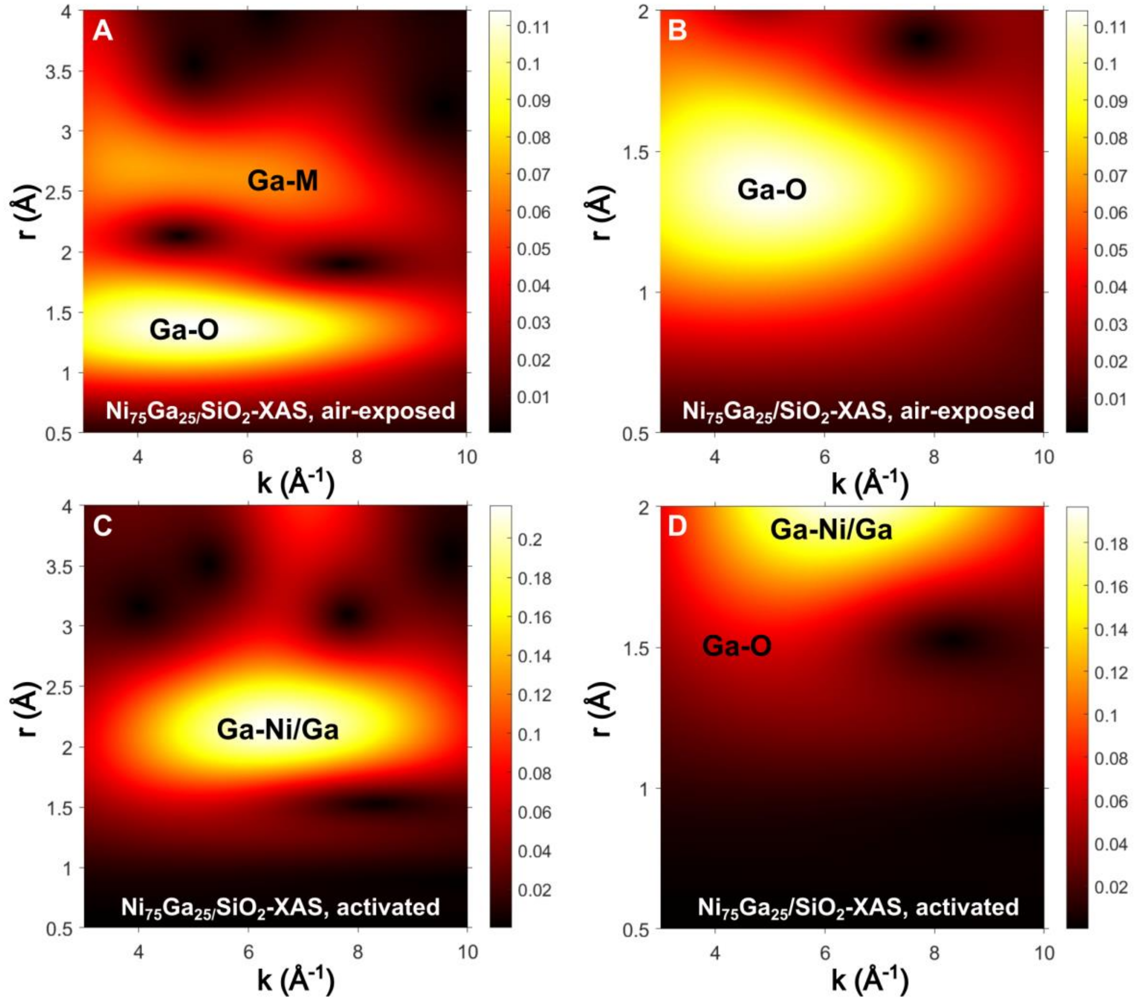

Figure S44. Continuous Cauchy wavelet transform of the k^2 weighted Ga K edge EXAFS of air-exposed (A-B, data collected at 50°C in 1bar H_2), and in situ activated (C-D, data collected at 50°C in 1 bar H_2) $Ni_{75}Ga_{25}/SiO_2$-XAS. B and D show an inset in the r-region 0.5-2 Å (Ga-O). In Ga-M in A), M stands for Ni, Ga and/or Si.

4 Diffuse Reflectance Infrared Fourier Transform Spectroscopy

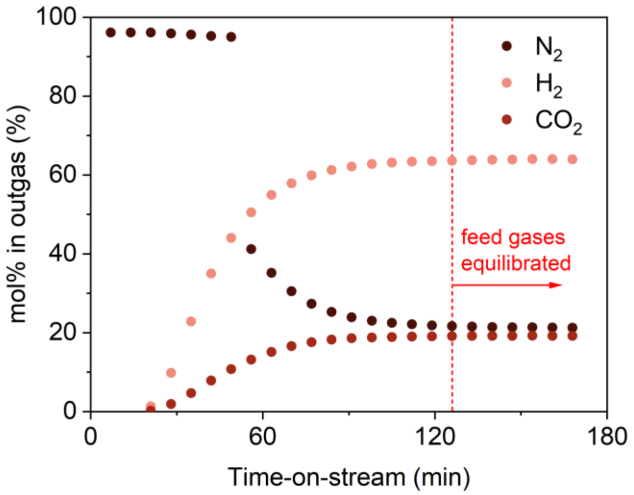

Figure S45. Off-gas composition measured during the operando DRIFTS experiment with an empty reaction cell. At TOS = 0 min the gas feed is switched from 20 bar N_2 (20 ml/min) to 20 bar CO_2:H_2:N_2 = 1:3:1 (20 ml/min). From this data we determined that the equilibration of CO_2, H_2 and N_2 after the gas switch takes ca. 126 minutes.

Table S10. DRIFTS band assignments.

**Wavenumber (cm^{-1})**			**Band assignment**	**Vibrational mode**	**Reference**
**$Ni_{65}Ga_{35}/SiO_2$**	**$Ni_{70}Ga_{30}/SiO_2$**	**$Ni_{75}Ga_{25}/SiO_2$**			
3650 – 3770			CO_2 (g)	Combination, $\nu_1 + \nu_3$	[21]
3500 – 3650			CO_2 (g)	Combination, $2\nu_2 + \nu_3$	[21]
-	-	3016	CH_4 (g)	Fundamental stretching, ν_3	[22]
2898-2900	-	-	b-HCOO on Ga^{3+}	ν(C-H)	[23,24]
2860, 2960	2860, 2962	2860, 2962	-OCH_3 on SiO_2	ν_{as}(C-H)	[25]
2200 – 2450			CO_2 (g)	Fundamental stretching, ν_3	[21]
2129			CO_2 (g)	Combination band	[21]
2094			CO_2 (g)	Combination band	[21]
2077			CO_2 (g)	Combination band	[21]
2050-2054	2060-2061	2060-2063	Adsorbed CO on Ni and gas phase $Ni(CO)_4$	ν(C-O)	[26–28]

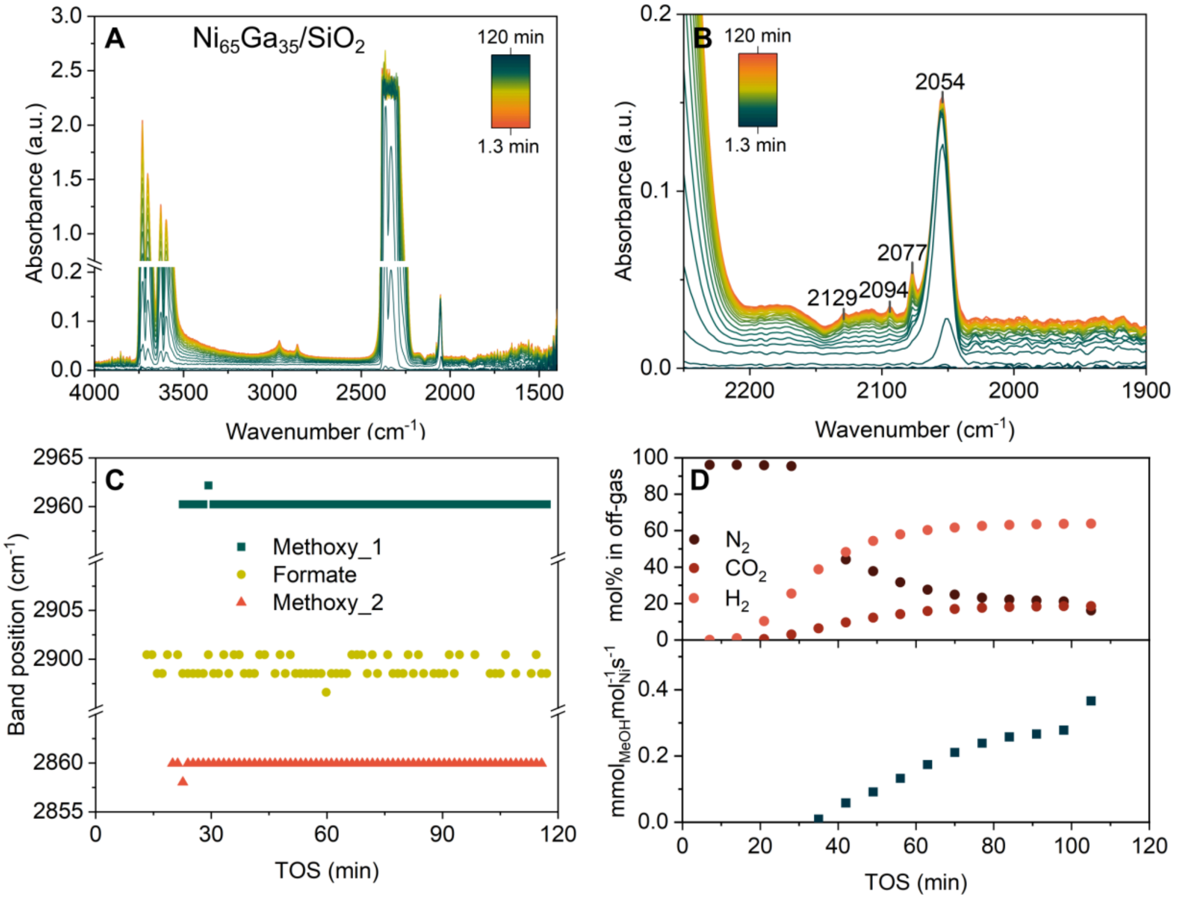

Figure S46. Operando DRIFTS data collected on $Ni_{65}Ga_{35}/SiO_2$ over 2 h TOS. (A) Full spectral range from 1400 cm^{-1} – 4000 cm^{-1}. (B) Spectral range between 1900 cm^{-1} – 2300 cm^{-1}. (C) Band positions as a function of TOS. (D) Off-gas composition measured at the outlet of the DRIFTS cell as a function of TOS. Conditions: 20 bar CO_2:N_2:H_2 = 1:1:3, 230°C.

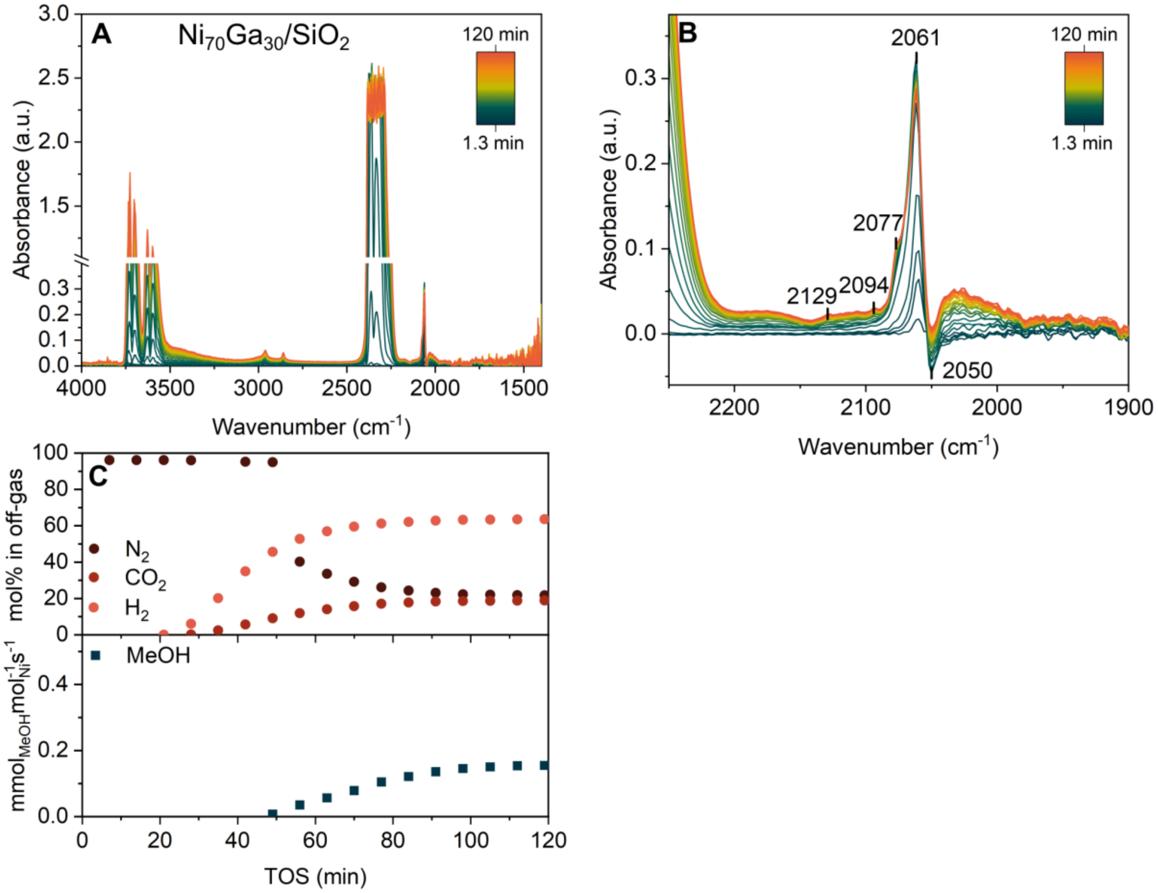

Figure S47. Operando DRIFTS data collected for $Ni_{70}Ga_{30}/SiO_2$ over 2 h TOS. (A) Full spectral range from 1400 cm^{-1} – 4000 cm^{-1}. (B) Spectral range between 1900 cm^{-1} – 2300 cm^{-1}. (C) Off-gas composition measured at the outlet of the DRIFTS cell as a function of TOS. Conditions: 20 bar $CO_2:N_2:H_2$ = 1:1:3, 230°C.

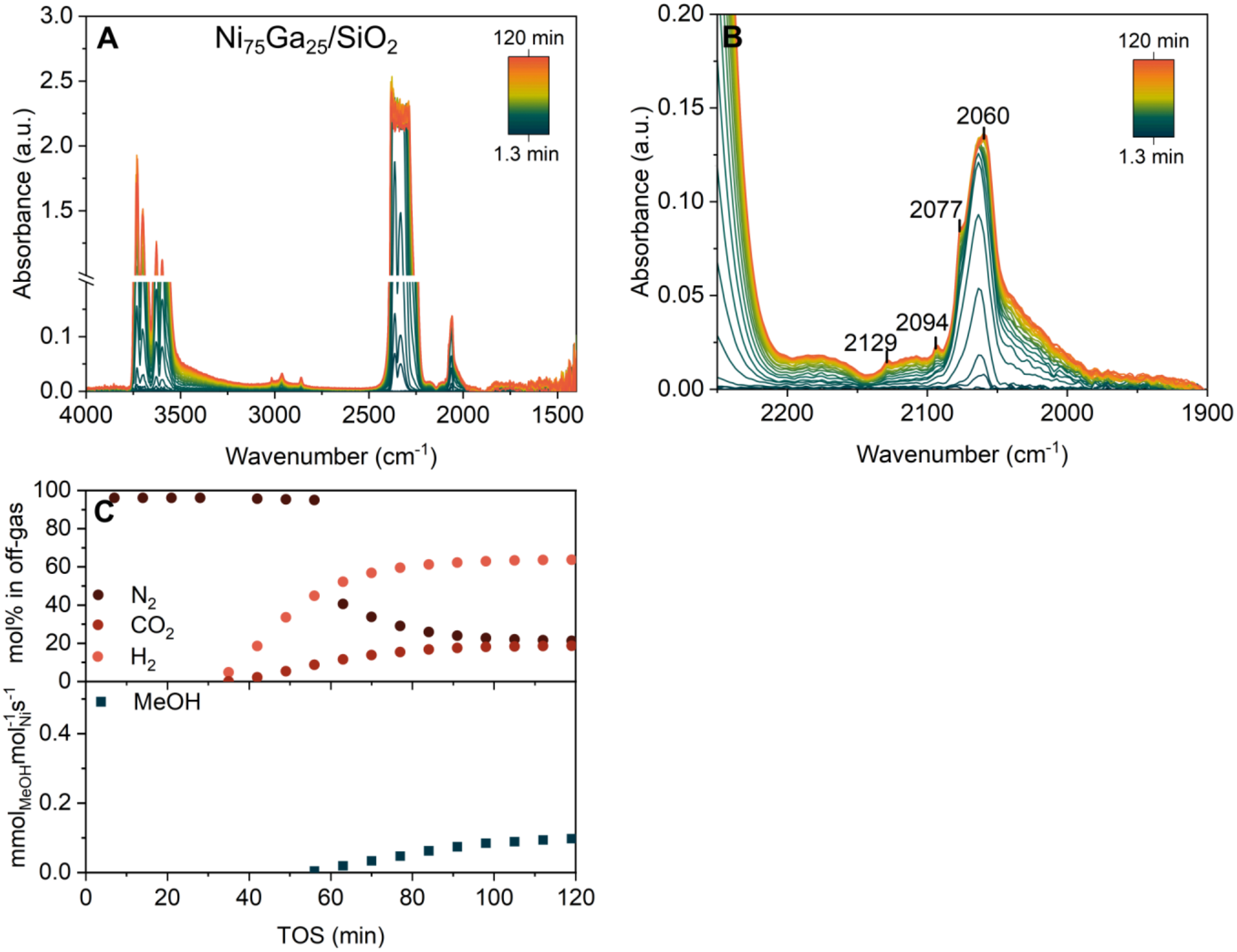

Figure S48. Operando DRIFTS data collected for $Ni_{75}Ga_{25}/SiO_2$ over 2 h TOS. (A) Full spectral range from 1400 cm^{-1} – 4000 cm^{-1}. (B) Spectral range between 1900 cm^{-1} – 2300 cm^{-1}. (D) Off-gas composition measured at the outlet of the DRIFTS cell as a function of TOS. Conditions: 20 bar CO_2:N_2:H_2 = 1:1:3, 230°C.

References

(1) Studt, F.; Sharafutdinov, I.; Abild-Pedersen, F.; Elkjær, C. F.; Hummelshøj, J. S.; Dahl, S.; Chorkendorff, I.; Nørskov, J. K. Discovery of a Ni-Ga Catalyst for Carbon Dioxide Reduction to Methanol. *Nat. Chem.* **2014**, *6* (4), 320–324. https://doi.org/10.1038/nchem.1873.

(2) Gallo, A.; Snider, J. L.; Sokaras, D.; Nordlund, D.; Kroll, T.; Ogasawara, H.; Kovarik, L.; Duyar, M. S.; Jaramillo, T. F. Ni_5Ga_3 Catalysts for CO_2 Reduction to Methanol: Exploring the Role of Ga Surface Oxidation/Reduction on Catalytic Activity. *Appl. Catal., B* **2020**, *267*, 118369. https://doi.org/10.1016/j.apcatb.2019.118369.

(3) Docherty, S. R.; Phongprueksathat, N.; Lam, E.; Noh, G.; Safonova, O. V.; Urakawa, A.; Copéret, C. Silica-Supported PdGa Nanoparticles: Metal Synergy for Highly Active and Selective CO_2-to-CH_3OH Hydrogenation. *JACS Au* **2021**, *1* (4), 450–458.

(4) Lam, E.; Noh, G.; Wing Chan, K.; Larmier, K.; Lebedev, D.; Searles, K.; Wolf, P.; V. Safonova, O.; Copéret, C. Enhanced CH_3OH Selectivity in CO_2 Hydrogenation Using Cu-Based Catalysts Generated via SOMC from Ga III Single-Sites. *Chem. Sci.* **2020**, *11* (29), 7593–7598. https://doi.org/10.1039/D0SC00465K.

(5) Lam, E.; Noh, G.; Larmier, K.; Safonova, O. V.; Copéret, C. CO_2 Hydrogenation on Cu-Catalysts Generated from ZnII Single-Sites: Enhanced CH_3OH Selectivity Compared to Cu/ZnO/Al_2O_3. *J. Catal.* **2021**, *394*, 266–272. https://doi.org/10.1016/j.jcat.2020.04.028.

(6) Jangam, A.; Hongmanorom, P.; Hui Wai, M.; Jeffry Poerjoto, A.; Xi, S.; Borgna, A.; Kawi, S. CO_2 Hydrogenation to Methanol over Partially Reduced Cu-SiO_{2P} Catalysts: The Crucial Role of Hydroxyls for Methanol Selectivity. *ACS Appl. Energy Mater.* **2021**, *4* (11), 12149–12162. https://doi.org/10.1021/acsaem.1c01734.

(7) Juhás, P.; Davis, T.; Farrow, C. L.; Billinge, S. J. L. PDFgetX3: A Rapid and Highly Automatable Program for Processing Powder Diffraction Data into Total Scattering Pair Distribution Functions. *J. Appl. Crystallogr.* **2013**, *46* (2), 560–566. https://doi.org/10.1107/S0021889813005190.
(8) Juhás, P.; Davis, T.; Billinge, S. J. L. PDFgetX3. https://www.diffpy.org/products/pdfgetx.html.
(9) Farrow, C. L.; Juhas, P.; Liu, J. W.; Bryndin, D.; Božin, E. S.; Bloch, J.; Proffen, T.; Billinge, S. J. L. PDFfit2 and PDFgui: Computer Programs for Studying Nanostructure in Crystals. *J. Phys.: Condens. Matter* **2007**, *19* (33), 335219. https://doi.org/10.1088/0953-8984/19/33/335219.
(10) Farrow, C. L.; Juhás, P.; Liu, J. W.; Bryndin, D.; Božin, E. S.; Bloch, J.; Proffen, Th.; Billinge, S. J. L. PDFgui.
(11) Ravel, B.; Newville, M. ATHENA, ARTEMIS, HEPHAESTUS: Data Analysis for X-Ray Absorption Spectroscopy Using IFEFFIT. *J Synchrotron Rad* **2005**, *12* (4), 537–541. https://doi.org/10.1107/S0909049505012719.
(12) Iglesias-Juez, A.; Chiarello, G. L.; Patience, G. S.; Guerrero-Pérez, M. O. Experimental Methods in Chemical Engineering: X-Ray Absorption Spectroscopy—XAS, XANES, EXAFS. *Can. J. Chem. Eng.* **2022**, *100* (1), 3–22. https://doi.org/10.1002/cjce.24291.
(13) Deguchi, H.; Yoshida, H.; Inagaki, T.; Horiuchi, M. EXAFS Study of Doped Ceria Using Multiple Data Set Fit. *Solid State Ionics* **2005**, *176* (23), 1817–1825. https://doi.org/10.1016/j.ssi.2005.04.043.
(14) Muñoz, M.; Argoul, P.; Farges, F. Continuous Cauchy Wavelet Transform Analyses of EXAFS Spectra: A Qualitative Approach. *Am. Mineral.* **2003**, *88* (4), 694–700. https://doi.org/10.2138/am-2003-0423.
(15) Voronin, V. I.; Berger, I. F.; Proskurnina, N. V.; Goschitskii, B. N. Defects in a Lattice of Pure Nickel Subjected to Fast-Neutron Irradiation Followed by Annealings: Neutron-Diffraction Examination. *Phys. Metals Metallogr.* **2016**, *117* (4), 348–354. https://doi.org/10.1134/S0031918X16040141.
(16) Mishima, Y.; Ochiai, S.; Suzuki, T. Lattice Parameters of Ni(γ), Ni_3Al(γ') and Ni_3Ga(γ') Solid Solutions with Additions of Transition and B-Subgroup Elements. *Acta Metall.* **1985**, *33* (6), 1161–1169. https://doi.org/10.1016/0001-6160(85)90211-1.
(17) $Ni_{92}Ga_{08}$. *Bull. Res. Lab. Precis. Mach. Electron. (Tokyo Inst. Technol.)* **1984**, *53*, 15–28.
(18) Buschow, K. H. J.; van Engen, P. G.; Jongebreur, R. Magneto-Optical Properties of Metallic Ferromagnetic Materials. *Journal of Magnetism and Magnetic Materials* **1983**, *38* (1), 1–22. https://doi.org/10.1016/0304-8853(83)90097-5.
(19) Pearson, W. B.; Thompson, L. T. The Lattice Spacings of Nickel Solid Solutions. *Can. J. Phys.* **1957**, *35* (4), 349–357. https://doi.org/10.1139/p57-040.
(20) $Ni_{70}Ga_{30}$. *Zeitschrift für Metallkunde* **1950**, *41*, 480–484.
(21) Fehr, S. M.; Krossing, I. Spectroscopic Signatures of Pressurized Carbon Dioxide in Diffuse Reflectance Infrared Spectroscopy of Heterogeneous Catalysts. *ChemCatChem* **2020**, *12* (9), 2622–2629. https://doi.org/10.1002/cctc.201902038.
(22) Plyler, E. K.; Tidwell, E. D.; Blaine, L. R. Infrared Absorption Spectrum of Methane From 2470 to 3200 Cm^{-1}. *J. Res. Natl. Bur. Stand. A Phys. Chem.* **1960**, *64A* (3), 201–212. https://doi.org/10.6028/jres.064A.021.
(23) Collins, S. E.; Baltanás, M. A.; Bonivardi, A. L. An Infrared Study of the Intermediates of Methanol Synthesis from Carbon Dioxide over Pd/β-Ga_2O_3. *J. Catal.* **2004**, *226* (2), 410–421. https://doi.org/10.1016/j.jcat.2004.06.012.
(24) Collins, S. E.; Briand, L. E.; Gambaro, L. A.; Baltanás, M. A.; Bonivardi, A. L. Adsorption and Decomposition of Methanol on Gallium Oxide Polymorphs. *J. Phys. Chem. C* **2008**, *112* (38), 14988–15000. https://doi.org/10.1021/jp801252d.
(25) Monti, D. M.; Cant, N. W.; Trimm, D. L.; Wainwright, M. S. Hydrogenolysis of Methyl Formate over Copper on Silica: I. Study of Surface Species by in Situ Infrared Spectroscopy. *J. Catal.* **1986**, *100* (1), 17–27. https://doi.org/10.1016/0021-9517(86)90067-9.
(26) Mihaylov, M.; Hadjiivanov, K.; Knözinger, H. Formation of $Ni(CO)_4$ during the Interaction between CO and Silica-Supported Nickel Catalyst: An FTIR Spectroscopic Study. *Catal. Lett.* **2001**, *76* (1), 59–63. https://doi.org/10.1023/A:1016786023456.
(27) Galhardo, T. S.; Braga, A. H.; Arpini, B. H.; Szanyi, J.; Gonçalves, R. V.; Zornio, B. F.; Miranda, C. R.; Rossi, L. M. Optimizing Active Sites for High CO Selectivity during CO_2 Hydrogenation over Supported Nickel Catalysts. *J. Am. Chem. Soc.* **2021**, *143* (11), 4268–4280. https://doi.org/10.1021/jacs.0c12689.
(28) Rasteiro, L. F.; Rossi, M. A. L. S.; Assaf, J. M.; Assaf, E. M. Low-Pressure Hydrogenation of CO_2 to Methanol over Ni-Ga Alloys Synthesized by a Surfactant-Assisted Co-Precipitation Method and a Proposed Mechanism by DRIFTS Analysis. *Catal. Today* **2021**, *381*, 261–271. https://doi.org/10.1016/j.cattod.2020.05.067.
